# DNA methylation-based estimator of telomere length

**DOI:** 10.18632/aging.102173

**Published:** 2019-08-18

**Authors:** Ake T. Lu, Anne Seeboth, Pei-Chien Tsai, Dianjianyi Sun, Austin Quach, Alex P. Reiner, Charles Kooperberg, Luigi Ferrucci, Lifang Hou, Andrea A. Baccarelli, Yun Li, Sarah E. Harris, Janie Corley, Adele Taylor, Ian J. Deary, James D. Stewart, Eric A. Whitsel, Themistocles L. Assimes, Wei Chen, Shengxu Li, Massimo Mangino, Jordana T. Bell, James G. Wilson, Abraham Aviv, Riccardo E. Marioni, Kenneth Raj, Steve Horvath

**Affiliations:** 1 Department of Human Genetics, David Geffen School of Medicine, University of California Los Angeles, Los Angeles, CA 90095, USA; 2 Centre for Genomic and Experimental Medicine, Institute of Genetics & Molecular Medicine, University of Edinburgh, Edinburgh EH4 2XU, UK; 3 Department of Twin Research and Genetic Epidemiology, Kings College London, London SE1 7EH, UK; 4 Department of Biomedical Sciences, Chang Gung University, Taoyuan, Taiwan; 5 Genomic Medicine Research Core Laboratory, Chang Gung Memorial Hospital, Linkou, Taiwan; 6 Department of Epidemiology and Biostatistics, School of Public Health, Peking University Health Science Center, Beijing, China; 7 Department of Epidemiology, Tulane University School of Public Health and Tropical Medicine, New Orleans, LA 70112, USA; 8 Public Health Sciences Division, Fred Hutchinson Cancer Research Center, Seattle, WA 98109, USA; 9 Longitudinal Studies Section, Translational Gerontology Branch, National Institute on Aging, National Institutes of Health, Baltimore, MD 21224, USA; 10 Center for Population Epigenetics, Robert H. Lurie Comprehensive Cancer Center and Department of Preventive Medicine, Northwestern University Feinberg School of Medicine, Chicago, IL 60611, USA; 11 Laboratory of Environmental Epigenetics, Departments of Environmental Health Sciences Epidemiology, Columbia University Mailman School of Public Health, New York, NY 10032, USA; 12 Departments of Genetics, Biostatistics, Computer Science, University of North Carolina, Chapel Hill, NC 27599, USA; 13 Centre for Cognitive Ageing and Cognitive Epidemiology, Department of Psychology, University of Edinburgh, Edinburgh EH8 9JZ, UK; 14 Department of Psychology, University of Edinburgh, Edinburgh EH8 9JZ, UK; 15 Department of Epidemiology, Gillings School of Global Public Health, University of North Carolina, Chapel Hill, NC 27599, USA; 16 Department of Medicine, School of Medicine, University of North Carolina, Chapel Hill, NC 27599, USA; 17 VA Palo Alto Health Care System, Palo Alto, CA 94304, USA; 18 Department of Medicine, Stanford University School of Medicine, Stanford, CA 94305, USA; 19 Children’s Minnesota Research Institute, Children’s Hospitals and Clinics of Minnesota, Minneapolis, MN 55404, USA; 20 Department of Physiology and Biophysics, University of Mississippi Medical Center, Jackson, MS 39216, USA; 21 Center of Development and Aging, New Jersey Medical School, Rutgers State University of New Jersey, Newark, NJ 07103, USA; 22 Radiation Effects Department, Centre for Radiation, Chemical and Environmental Hazards, Public Health England, Chilton, Didcot, Oxfordshire OX11 0RQ, UK; 23 Department of Biostatistics, Fielding School of Public Health, University of California Los Angeles, Los Angeles, CA 90095, USA

**Keywords:** telomere length, DNA methylation, molecular biomarker, aging

## Abstract

Telomere length (TL) is associated with several aging-related diseases. Here, we present a DNA methylation estimator of TL (DNAmTL) based on 140 CpGs. Leukocyte DNAmTL is applicable across the entire age spectrum and is more strongly associated with age than measured leukocyte TL (LTL) (*r ~*-0.75 for DNAmTL versus *r ~* -0.35 for LTL). Leukocyte DNAmTL outperforms LTL in predicting: i) time-to-death (p=2.5E-20), ii) time-to-coronary heart disease (p=6.6E-5), iii) time-to-congestive heart failure (p=3.5E-6), and iv) association with smoking history (p=1.21E-17). These associations are further validated in large scale methylation data (n=10k samples) from the Framingham Heart Study, Women's Health Initiative, Jackson Heart Study, InChianti, Lothian Birth Cohorts, Twins UK, and Bogalusa Heart Study. Leukocyte DNAmTL is also associated with measures of physical fitness/functioning (p=0.029), age-at-menopause (p=0.039), dietary variables (omega 3, fish, vegetable intake), educational attainment (p=3.3E-8) and income (p=3.1E-5). Experiments in cultured somatic cells show that DNAmTL dynamics reflect in part cell replication rather than TL *per se*. DNAmTL is not only an epigenetic biomarker of replicative history of cells, but a useful marker of age-related pathologies that are associated with it.

## Introduction

Telomeres are repetitive nucleotide sequences at the end of chromosomes that shorten with replication of somatic cells. Since the number of cell replication *in vivo* increases with age, telomere length (TL) is negatively correlated with age of proliferating somatic cells. Meta-analysis of 124 cross-sectional studies and 5 longitudinal studies showed that the correlation between leukocyte telomere length (LTL) and age ranges between r=-0.295 and r=-0.338 across adults [1].

TL variation within somatic tissues of the individual is much smaller than that between individuals. Within the individual, TL variation across somatic tissues such as blood, skin, muscle and fat largely reflects their replicative history prior to adulthood, given that the rates of TL shortening in these tissues are similar during adulthood [2]. Shorter LTL is associated with cardiovascular disease, psychological stress, and lifespan [3–10].

Another DNA-based biomarker that changes with age is methylation of cytosine residues of cytosine-phosphate-guanine dinucleotides (CpGs). Machine learning-based analyses of these changes generated algorithms, known as epigenetic clocks that use specific CpG methylation levels to estimate age (i.e., DNAm age) [11–14] and/or physiological age [15–17]. Although both DNAm age and LTL are associated with chronological age, they exhibit only weak correlations with each other after adjusting for age [18–20], suggesting the distinct nature of their underlying mechanisms.

DNA methylation assays are already highly robust and ready for biomarker development [21]. By contrast, despite two decades of population-based telomere research, the measurement of TL remains challenging and can be subject to technical confounding factors including but not limited to different methods of DNA extraction [22–24]. Furthermore, the terminal restriction fragments (TRFs), measured by Southern blotting, the accepted ‘gold standard’ of TL measurements, include not only the canonical region of telomeres but also the potentially variable sub-telomeric region [22,25]. It would be ideal if the robustness inherent in DNA methylation analyses can be extended to TL measurement. Although there are reports of TL-related DNA methylation changes [26], it was unknown whether these reflect actual TL or associated biological features, including health outcomes.

We present here a novel DNAm TL estimator (DNAmTL) based on methylation profiles of 140 CpGs. This epigenetic biomarker was developed by regressing measured LTL on blood methylation data from n=2,256 individuals (training set). We show that DNAmTL correlates negatively with age in different tissues and cell types and outperforms TRF-based LTL in predicting mortality and time-to-heart disease, as well as being associated with smoking history and other age-related conditions.

We also validated the applicability of DNAmTL on a large-scale data set (N=9,345) and uncovered associations between age-adjusted DNAmTL with diet and clinical biomarkers.

Monitoring cultured cells with or without telomerase revealed that DNAmTL records cell replication independently of telomere attrition.

## RESULTS

### Training and validation data from 3 cohorts

In stage 1 of our project, we evaluated data from n=3,334 individuals for whom both LTL and Illumina methylation array data were available. These were from three different studies: Framingham Heart Study offspring cohort (FHS, N=878), Women’s Health Initiative (WHI, N=818) and Jackson Heart Study cohort (JHS, N=1638, Table 1 and Supplementary Note 1). The same laboratory measured LTL by Southern blotting of the terminal restriction fragments [25]. DNA methylation levels were measured in different labs using the Illumina Infinium methylation array platform.

**Table 1:** Overview of training and test data.

						**Telomere length statistics**		
**Data**	**N**	**Female**	**Race**	**Age**	**Array** **Normalization**	**LTL**	**DNAmTL**	**Corr**
***Train***								
WHI BA23	718	100%	EUR (59%) AFR (41%)	66.5 (50.2,80.2)	GenomeStudio	6.9 (5.2,9.1)	6.9 (6.0, 7.8)	0.62
JHS	1538	64%	AFR	56.6 (22.2,93.1)	Noob [68]	7.1 (4.9,10)	7.2 (5.9, 8.1)	0.62
***Test***								
FHS	878	51%	EUR	57.0 (33.0,82.0)	Noob [68]	7 (5.5,8.7)	6.8 (5.4, 8.1)	0.44
WHI BA23	100	100%	EUR (49%) AFR (51%)	65.3 (51.9,79.8)	GenomeStudio	6.9 (5.6,9)	6.9 (6.2, 7.5)	0.41
JHS	100	55%	AFR	53.5 (22.9,80)	Noob [68]	7.2 (5.6,9)	7.1 (6.6, 7.8)	0.50
***Validation analysis***								
FHS^a,b^	2356	54%	EUR	66.4 (40, 92)		7.0 (5.5,8.7)	6.8 (5.4, 8.1)	0.44
WHI^a^ BA23	1389	100%	EUR (41%) AFR (28%) HISP (31%)	65.4 (50.1, 80.2)	Noob [68]	6.9 (5.6,9)	6.9 (6.2, 7.5)	0.41
WHI EMPC	1972	100%	EUR (56%) AFR (28%) HISP(16%)	62.9 (49.5, 82.0)	BMIQ [57]	--	--	--
JHS^a^	209	56%	AFR	59.8 (22.9, 84.6)	Noob [68]	7.2 (5.6,9)	7.1 (6.6, 7.8)	0.50
InChianti^c^	924 (484)	54%	EUR	72 (21, 100)	Noob [68]	--	--	--
Twins UK^d^	794	100%	EUR	57.2 (24.0,81.1)	BMIQ [57]	3.5 (1.7, 6.4)	7.0 (5.6, 7.9)	0.38
LBC 1921^e^	436	60%	EUR	79.1 (77.7, 80.6)	Noob [68]	4.1 (1.9, 5.3)	6.6 (5.7, 7.4)	-0.01
LBC 1936^e^	906	50%	EUR	69.6 (67.6, 71.3)	Noob [68]	4.1 (2.7, 7.1)	6.7 (6.1, 7.5)	0.08
BHS	831	57%	EUR (70%) AFR (30%)	43.8 (28.4, 54.6)	watermelon [69]	6.9 (5.2, 9.5)	7.0 (6.4, 7.6)	0.43

AfricanA=African American; EUR=European; HISP=Hispanics; LTL=leukocyte telomere length.

The distributions of Age, LTL, and DNAmTL are presented in median (range) format.

^a^The validation set consists of two groups of individuals: (1) those individuals with TL measures that were included in test process, (2) those individuals without TL measures.

^b^The age distribution was based on exam 8.

^c^The statistics are based on the number of 924 observations across 484 individuals.

^d^Of the 794 individuals, 779 were available with LTL measures.

^e^All subjects of the Lothian *Birth* Cohorts were born at roughly the same time (within 1.9 to 3.7 years).

We display characteristics of (1) 3334 study participants in the training and test data sets that were used to develop and validate DNAmTL, and (2) 9345 participants (9875 blood samples) from 9 cohorts across 7 studies. The participated studies include Framingham Heart Study (FHS) offspring cohort, Women's Health Initiative (WHI), Jackson Heart Study (JHS), InChianti, Twins UK, Lothian Birth Cohorts (LBC), and Bogalusa Heart Study (BHS). Leukocyte TL measures were based on terminal restriction fraction measurement by Southern blotting Southern blotting in FHS, WHI, JHS, and BHS and were based on quantitative real-time polymerase chain reaction (qPCR) in Twins UK and LBC. The column "Array Normalization" refers to different methods of pre-processing DNA methylation array data.

An overview of the data sets is found in Table 1. These US cohorts were comprised of two ethnic groups: 41% of European ancestry and 59% of African Ancestry. The age of the individuals ranged from 22 to 93 years. The training set used for constructing DNAmTL was comprised of N=2,256 individuals from the WHI and JHS cohorts for whom LTL and DNAm data were assessed from the same blood sample (collected at the same time). Although fewer than 20% of individuals in the training set were of European ancestry, our test data demonstrated that the resulting DNAmTL estimator applied equally well to individuals of European ancestry. We used two test data sets. The first test data set involved N=1,078 individuals comprised of N=100 from the WHI, N=100 from JHS, and N=878 from the FHS cohorts. The second test data set was collected in stage 2 of our analysis: it involved N=9,815 DNA methylation samples from additional cohorts (Bogalusa, Twins UK, Lothian Birth cohorts, InCHIANTI) to evaluate correlations between LTL and numerous age-related conditions and lifestyle factors. We also evaluated DNAmTL in publicly-available data from adipose tissue (N=648 from the Twins UK study [27,28]), liver (N=85) [28,29], and monocytes (n=1264 from the Multi-Ethnic Study of Atherosclerosis) [30]. Finally, we tested DNAmTL in *in vitro* studies to ascertain its applicability to cultured cells and to probe the nature of DNAmTL’s association with TL. Additional details of these studies can be found in Supplementary Notes 1 and 2.

### DNAmTL versus measured TL in blood and adipose tissue

**Figure 1:**
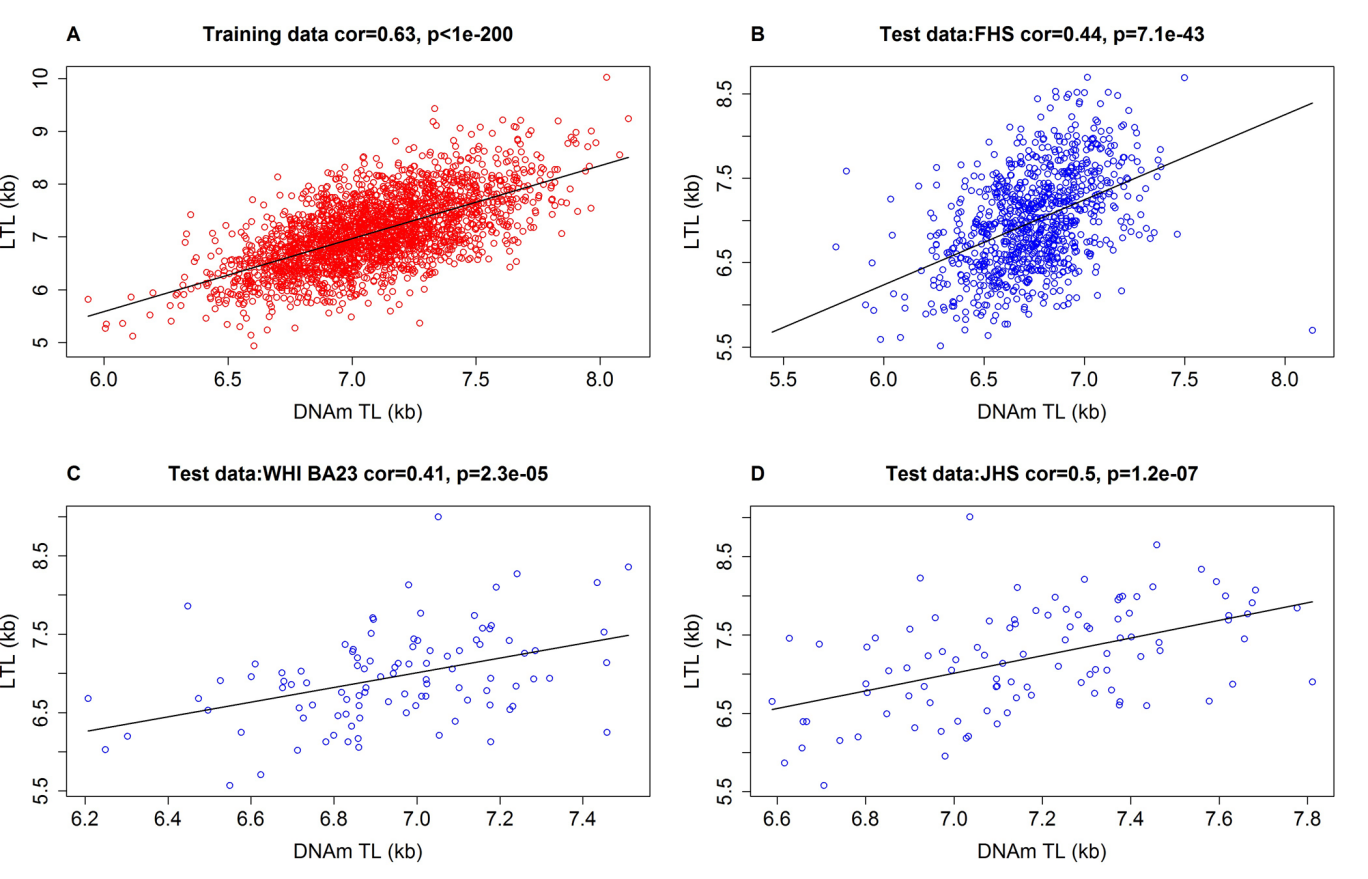
**Measured LTL versus DNAmTL in training and test datasets.** Scatter plots of DNA methylation-based telomere length (DNAmTL, x-axis) versus observed LTL measured by terminal restriction fragmentation (y-axis). DNAmTL and LTL are in units of kilobase (kb). (**A**) Training data. (**B**) Test data from the Framingham Heart Study. (**C**) Test data from the Women's Health Initiative (BA23 sub-study). (**D**) Test data from the Jackson Heart Study. Each panel reports a Pearson correlation coefficient and correlation test p-value. Table 1 reports analogous results for additional cohorts (Bogalusa, Twins UK, etc).

We restricted the analysis to CpGs that are present on both the Illumina Infinium 450K array and the Illumina EPIC methylation array (Methods). Using the training data (n=2,256), we regressed measured LTL (mean TRFs) on blood CpG methylations using an elastic net regression model [31]. This resulted in the automatic selection of 140 CpGs whose methylation levels best-predicted LTL (Supplementary data 1). The linear regression model allows a direct prediction of TL based on DNA methylation levels. The predicted TL value, also referred to as DNAmTL, possesses the same units (kilobase) as that of mean TRF. The correlation coefficient between DNAmTL and LTL in the training data was r=0.63, which was overly optimistic, as subsequent independent validation with test data sets produced lower correlations of r>0.40 (Figure 1). Further, using 12 large validation data sets, we found that the correlation between DNAmTL and LTL ranged from r=0.38 to r=0.5 (last column of Table 1) with the exception of the Lothian Birth cohorts (where it was close to zero). The correlations between DNAmTL and LTL were not confounded by age, as was evident from the high correlations between age-adjusted DNAmTL and age-adjusted LTL (e.g. r=0.34 in FHS and r=0.43 in Bogalusa Herat Study, Supplementary Figures 1A and 2A and E). A stratified analysis showed that the correlation between DNAmTL and LTL were neither confounded by sex (Supplementary Figures 12B and C) nor ethnicity (Supplementary Figure 2D and E). The DNAmTL biomarker was robust against potential effects of pre-processing steps in the DNAm data analysis as can be seen from the diverse normalization methods used by the different cohorts (Table 1). The DNAmTL measurement was also robust across time as can be seen with the FHS cohort where the blood samples for the LTL measurement (FHS exam 6) were collected 9.3 years earlier than those used for the DNAm measurement (FHS exam 8). This time lag biased the correlation toward the null hypothesis, i.e. generated a correlation (r=0.44, Figure 1B) that was overly conservative. A separate analysis of non-blood tissue revealed a higher correlation of r=0.65 between DNAmTL and TRF-based in adipose tissue samples from the Twins UK study (Supplementary Figure 3B).

### DNAmTL across different blood cell types

To test whether DNAmTL differs across blood types, we used sorted blood cells and peripheral blood mononuclear cells (PBMCs) from 6 men (aged between 27 and 32 years old, Methods). We observed a statistically significant difference in median DNAmTL values (p=0.0033, Supplementary Figure 4) even though they were roughly comparable: CD8+ T cells (median=8.25), CD4+ T cells (median=7.64), B cells (median DNAmTL=7.43), PBMCs (median DNAmTL=7.55).

### DNAmTL versus qPCR TL in the Twins UK study

We obtained leukocyte DNAm data from 792 participants (all women) from the Twins UK study whose LTL was measured by Southern blotting (N=346) and/or quantitative polymerase chain reaction (qPCR, N=779) (Supplementary Note 1). The correlation between DNAmTL and qPCR- based LTL (r=0.39, Supplementary Figure 5A) was similar to that of LTL measured by Southern blotting (r=0.40, Supplementary Figure 5B).

### DNAmTL correlates more strongly with age than TL

**Figure 2:**
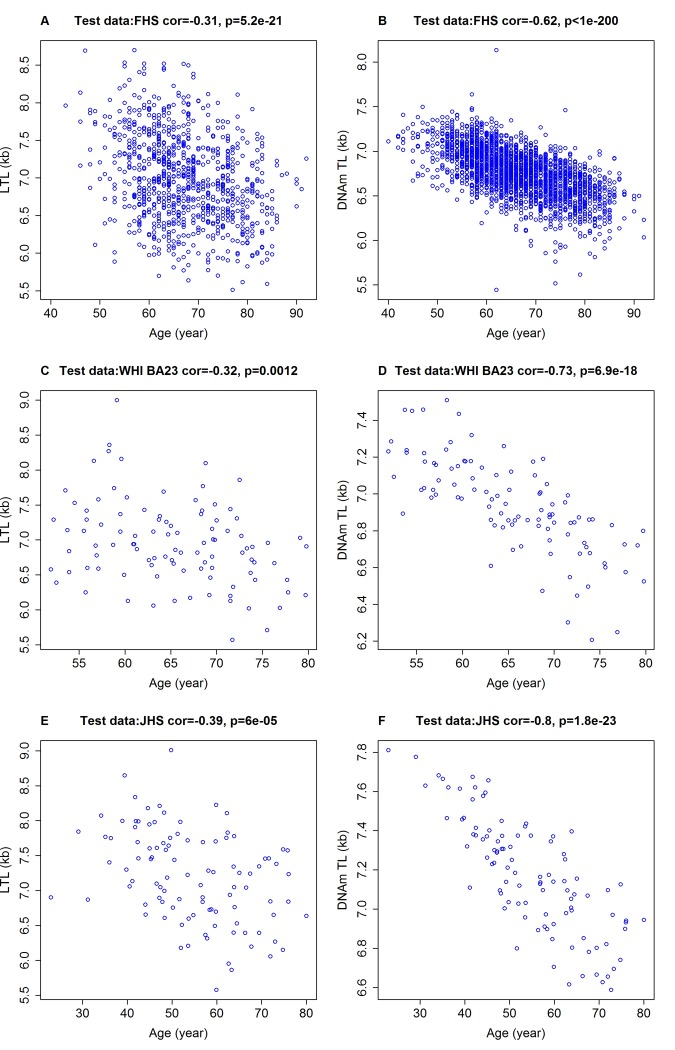
**Chronological age versus measured LTL and DNAmTL.** Chronological age versus measured LTL (panels **A, C, E,** in units of kilobase [kb]) and DNAmTL (panels **B, D, F,** in units of kb). (**A, B**) Test data from the FHS. (**C, D**) Test data from the WHI (N=100), (**E, F**) Test data from the JHS (N=100). Each panel reports a Pearson correlation coefficient and correlation test p-value.

Although DNAmTL was developed based on LTL, it displayed substantially stronger negative correlations with age at the time of blood draw (*r ~* -0.80 to -0.62) than did measured LTL, based on our test datasets (*r ~* -0.40 to -0.30, Figure 2). Multivariate regression models in the test data show that LTL shortened by 0.022 kilobases per year (p=2.3E-27) after adjusting for sex, race/ethnicity and other confounders (Table 2). Analogous multivariate regression models showed that DNAmTL reduced by 0.018 kilobases per year, but this was associated with a far more significant p value (p=6.0E-125) than that of measured LTL (p=2.3E-27). Although the DNAm-based biomarkers were derived from profiles of adults (22-93 years old), the resulting DNAmTL algorithm was equally applicable to profiles from children; even to those who were younger than 13 years of age, where a strong negative correlation of r=-0.81 was observed between DNAmTL of blood and age (Supplementary Figure 6A). Such expected negative correlations with age were also seen with DNAmTL in adipose tissue (r=-0.41. Supplementary Figure 3A), liver (r=-0.71, Supplementary Figure 7A), and in (sorted) monocytes (r=-0.60, Supplementary Figure 8A).

**Table 2:** Multivariate regression analysis of leukocyte telomere length.

**Variable**	**Coefficient (SE)**	**t-statistic**	**P**
***Outcome: actual LTL (mean TRF)***			
Intercept	8.43 (0.201)	41.88	2.43E-227
Age	-0.022 (0.002)	-11.14	2.33E-27
Female	0.132 (0.036)	3.71	2.15E-4
Race: European	0.029 (0.112)	0.26	7.97E-1
smoke: Former	0.132 (0.063)	2.11	3.50E-2
smoke: Never	0.113 (0.062)	1.82	6.95E-2
BMI	-0.007 (0.003)	-2.15	3.16E-2
JHS	0.005 (0.126)	0.04	9.66E-1
WHI BA23	-0.093 (0.084)	-1.10	2.73E-1
***Outcome: DNAmTL***			
Intercept	8.046 (0.069)	116.16	<1.0E-300
Age	-0.018 (0.001)	-27.32	5.97E-125
Female	0.099 (0.012)	8.14	1.14E-15
Race: European	-0.136 (0.039)	-3.52	4.57E-4
smoke: Former	0.08 (0.022)	3.72	2.09E-4
smoke: Never	0.096 (0.021)	4.51	7.11E-6
BMI	-0.002 (0.001)	-2.19	2.91E-2
JHS	0.069 (0.044)	1.59	1.11E-1
WHI BA23	0.049 (0.029)	1.69	9.15E-2

BMI=body mass index; SE=standard error.

In the upper panel, we present results from a multivariate linear regression model analysis of actual LTL (mean TRF, dependent variable) on different covariates (rows) in the test data set (comprised of 1078 individuals). The model was regressed on age, sex, race/ethnicity, smoking status, and study cohort. Race/ethnicity is a dichotomized variable (European versus African Ancestry). Smoking status is a three-category variable: never, former and current smokers (as a reference). Study cohort is a trivariate variable (FHS, WHI BA23 and JHS cohort).

In the lower panel, we present the analogous multivariate model but its dependent variable is DNAmTL.

### Effect of sex and ethnicity

Because age would confound any potential relationship between DNAmTL and age-related traits such as health, it would be useful to derive an age-adjusted estimate of DNAmTL (referred to as DNAmTL*adjAge*). We therefore regressed DNAmTL on age and the resulting raw residual was defined as DNAmTLadjAge. A negative value of DNAmTLadjAge would indicate DNAmTL that is shorter than expected based on age, while a positive value would indicate the opposite. We noted that DNAmTLadjAge is heritable (heritability $h^{2}$=0.46, p=4.5E-11) according to a pedigree-based polygenic model analysis in the FHS cohort (N>2000, Methods).

Women tend to exhibit longer LTL than men of the same age [32]. Similarly, our multivariate regression models revealed that age-adjusted LTL and age-adjusted DNAmTL were indeed longer in females than in males. The p-values for age-adjusted LTL measured in this study (p=2.15E-4, N=1078) and that of a previous study [32] (p=5E-3, N ~ 730) were far less significant than those for age-adjusted DNAmTL (p=1.14E-15, Table 2). Roughly, women showed longer telomere length than men by 0.1 kilobase, given by DNAm or TRF measures (Table 2). We also found longer age-adjusted DNAmTL in female compared to male liver samples (P=0.017, Supplementary Figure 7C). With regards to ethnicity, age-adjusted DNAmTL of PBMCs (Table 2) and monocytes (Supplementary Figure 8B) revealed that US population of African ancestry have a longer LTL than those of European ancestry, consistent with previous observations made with TRF-based measured TL. Once again, the association of age-adjusted DNAmTL (p=1.6E-33, N ~ 1200) with ethnicity was stronger than those seen with age-adjusted LTL measured by TRF (p=1E-4) or quantitative polymerase chain reaction (p=1E-3, N ~ 2450) [33]. Overall, these results demonstrate that DNAmTL exhibits substantially more significant associations with age, sex and ethnicity than measured LTL.

### DNAmTL is often superior to measured LTL in predicting mortality and health outcomes

**Figure 3:**
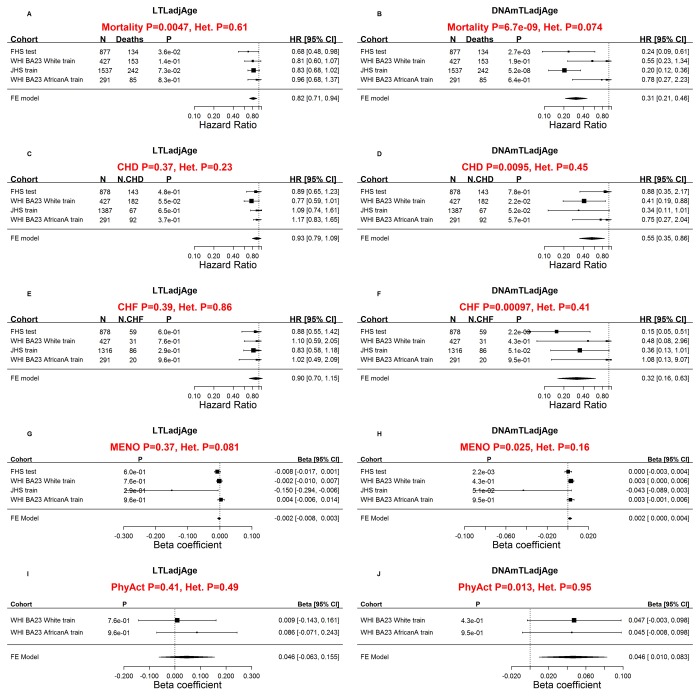
**Comparing measured LTL with DNAmTL with respect to age-related conditions.** Meta-analysis forest plots for relating age-related conditions (rows) to age-adjusted LTL (left panels) and age-adjusted DNAmTL (right panels). Panels in the first row (**A, B**) presents meta-analysis forest plots for Cox regression models of time-to-death. Meta-analysis of Cox regression models for (**C, D**) time-to-coronary heart disease (CHD) and (**E, F**) time-to-congestive heart failure (CHF). Rows in the forest plot correspond to training and test datasets (used for developing DNAmTL) stratified by race/ethnicity Each row presents the summary statistic at a (stratified) study da.taset and reports sample size (N), number of events, P value, hazard ratio and a 95% confidence interval resulting from a Cox regression model. (**G, H**) Meta-analysis for the association with age at menopause. (**I, J**) Meta-analysis for the association with self-reported physical activity status (yes/no). (**G**-**J**) Each row (study data set) presents the summary statistic, P value, beta coefficient and a 95% confidence interval resulting from a linear (mixed) regression model. In general, an insignificant Cochran Q test p-value (denoted by Het. P) is desirable because it suggests that results do not differ significantly across the strata. However, an insignificant Q test p-value could also reflect lack of statistical power.

Next, we compared the performance of DNAmTLadjAge with age-adjusted LTL in predicting time-to-death or time-to-heart disease in the training and test datasets (N=3,334) for which both measures were available (Figure 3). We found that longer DNAmTLadjAge was significantly associated with a lower hazard ratio (HR) for time-to-death, all-cause mortality (HR=0.31 and P=6.7E-9), time-to-coronary heart disease (CHD, HR=0.55 and p=9.5E-3), and time-to-congestive heart failure (CHF, HR=0.32 and p=9.7E-4, Figure 3). In women, later age at menopause was associated with significantly higher values of DNAmTLadjAge (p=0.025). Furthermore, physical activity was also positively associated with DNAmTLadjAge (p=0.013).

By comparison, the results from age-adjusted LTLs (LTLadjAge) were far less significant in predicting lifespan (HR=0.81 and p=4.7E-3 compared to HR=0.31 and p=6.7E-9 for DNAmTL, Figure 3A, B) and were *not* significantly associated with time-to-CHD, time-to-CHF, age at menopause and physical activity (p>0.3, Figure 3).

We want to emphasize that our comparison between DNAmTLadjAge and LTLadjAge involved the same set of individuals for whom both measures were available, i.e. each association test used the same sample size and distribution in age, sex, and ethnicity. These results show that DNAmTLadjAge outperforms LTLadjAge when it comes to predicting important health-related conditions. However, our comparative analysis was subject to a limitation: the measures of LTLadjAge and DNAmTLadjAge in the FHS cohort corresponded to two different blood samples collected at different time points. We addressed this limitation in two ways. First, we repeated the analysis by omitting the FHS data. In the resulting test data (n=100 samples from the WHI and n=100 samples from the JHS), DNAmTLadjAge continued to outperform LTLadjAge (Supplementary Table 1). Second, we compared DNAmTLadjAge with LTL in a host of additional cohorts (Bogalusa Heart Study, Twins UK, Lothian Birth Cohorts) as detailed below.

### Evaluating DNAmTL in large scale validation data

In the second phase of validation, we sought to test these associations with even larger, independent data sets. In total, we analyzed N=9,875 Illumina methylation arrays from blood samples of N=9,345 individuals from 9 cohorts across 7 studies: FHS, WHI BA23, WHI EMPC, JHS, InChianti, Lothian Birth Cohorts of 1921 and 1936 (LBC), UK Twins, and Bogalusa Heart Study (BHS, Table 1, Methods, and Supplementary Note 1). Of the samples, 4,039 individuals were available with measured LTL measurements based on Southern blot or quantitative polymerase chain reaction (qPCR). The data set was comprised of three different ethnic groups: European (77%), African (15%), and Hispanic (8%) ancestries. All but one cohort (BHS) were available for mortality analysis (N=9,044 methylation arrays on 8,514 individuals) with sufficient follow-up period. The mean chronological age at the time of the blood draw was 65.6 years and the mean follow-up time (for all-cause mortality) was 11.8 years (Supplementary Table 2). Once again, DNAmTL was negatively correlated with chronological age in all cohorts with sufficient variation in chronological age (-0.83 ≤r ≤-0.42), in which we excluded the Lothian Birth Cohort studies comprising individuals with similar ages (Supplementary Figure 9).

**Figure 4:**
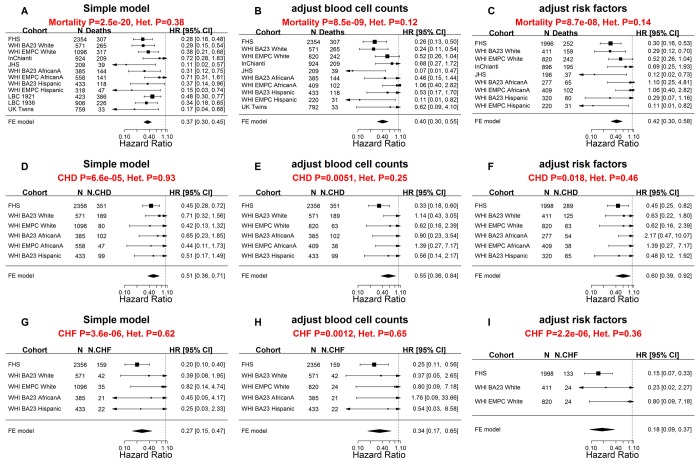
**Meta-analysis forest plots for predicting time-to-death due to all-cause mortality and time-to-cardiovascular disease in independent validation data.** Meta-analysis forest plot for combining Cox regression hazard ratios for time-to-death, time-to-coronary heart disease (CHD), and time-to-congestive heart failure (CHF), based on age-adjusted DNAmTL (DNAmTLadjAge). The sample sizes for the analysis were up to 9,044 methylation arrays (8,541 individuals) across 8 cohorts. Left panels, middle panels, and right panels report meta-analysis results for (1) simple Cox regression models, (2) multivariate Cox models adjusted for blood cell counts, and (3) multivariate Cox model adjusted for traditional risk factors, respectively. Each row reports the hazard ratio associated with DNAmTLadjAge. (1) The simple Cox models (left panels) were adjusted for chronological age, sex and adjusted for intra-pedigree correlation and batch effects as needed. (2) The models in the middle panels involved additional covariates: imputed blood cell counts based on DNA methylation data. (3) The models in the right panels different from those of (1) by additional demographic characteristics, psychosocial behavior, and clinical covariates (Methods). Each panel reports a meta-analysis forest plot for combining hazard ratios associated with time to event. Each row presents the summary statistic at a (stratified) study dataset and reports sample size (N), number of events, hazard ratio and a 95% confidence interval resulting from a Cox regression model. In general, an insignificant Cochran Q test p-value (denoted by Het. P) is desirable.

Further analyses of these data confirmed that higher values of DNAmTLadjAge were indeed associated with longer lifespan (Figure 4). Each kilobase increase of DNAmTLadjAge was associated with a hazard ratio of 0.37 for mortality (p=2.5E-20, Figure 4A), similar to what we observed in the training and test dataset (HR=0.31, Figure 3A). Higher values of DNAmTLadjAge were also associated with longer time-to-CHD (HR=0.51 and p=6.6E-5) and longer time-to-CHF (HR=0.27 and p=3.6E-6, Figure 4 D and G), mirroring yet again the results obtained from the training and test data sets.

Two types of multivariate Cox regression models demonstrated that these associations remained significant even after adjusting for (1) blood cell counts (Figure 4 B, E, H), and (2) classical risk factors (Figure 4 C, F, I) including body mass index, educational level, alcohol intake, smoking pack-years, prior history of diabetes, prior history of cancer, and hypertension status.

We went further and evaluated DNAmTLadjAge in different strata including age (younger/older than 65 years), prevalent clinical conditions at baseline and found that DNAmTLadjAge remained a significant predictor of time-to-death in each of these strata (Supplementary Table 3), e.g. HR=0.26 for individuals aged < 65 years and HR=0.41 for older individuals aged ≥ 65 years. DNAmTLadjAge also remained a significant predictor of time-to-CHF in most strata (Supplementary Table 4) and of time-to-CHD in specific strata such older age, normal BMI, or higher education attainment (Supplementary Table 5).

Our analyses revealed that higher DNAmTLadjAge values were associated with measures of physical fitness/functioning (P=7.6E-3), and disease-free status (p=0.019), while prior history of cancer was associated with lower DNAmTLadjAge values (p=0.053, Supplementary Figure 10). Interestingly, we also found a nominally significant association (p=0.026) between age-at-menopause and DNAmTLadjAge. We found that one year later age-at-menopause was associated 0.001 kilobase longer LTL (Supplementary Figure 10A). Our cross-sectional analyses, however, do not allow determination of cause-and-effect relationships, but we note that age-at-menopause is also associated with epigenetic aging [34].

### Life-style factors and clinical biomarkers

**Figure 5:**
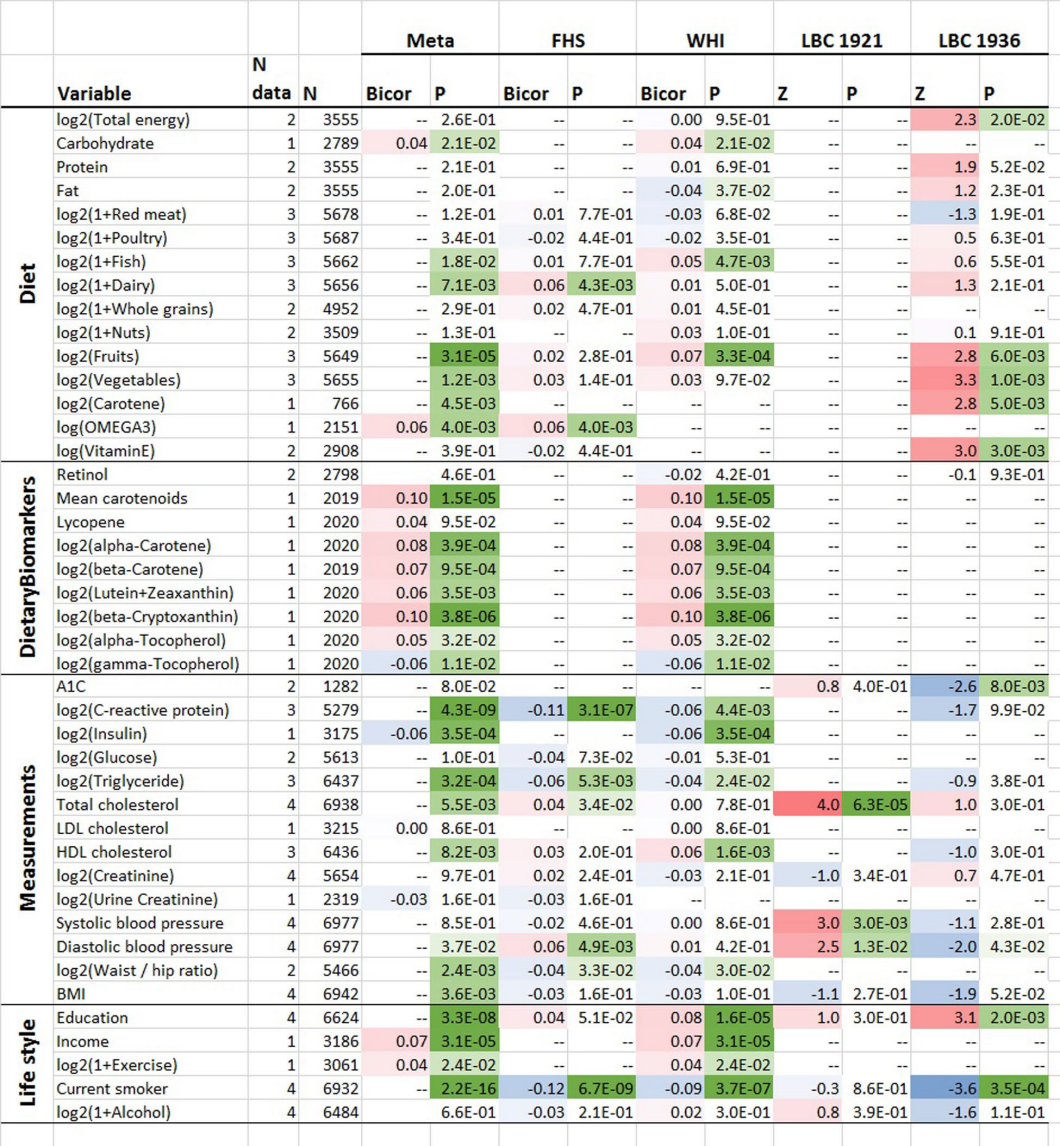
**Cross sectional associations between age-adjusted DNAmTL versus lifestyle/dietary variables.** Association analysis between age-adjusted DNAmTL (DNAmTLadjAge) and 43 variables including 15 self-reported diet, 9 dietary biomarkers, 14 variables related to metabolic traits and central adiposity, and 5 life style factors, based on the meta-analysis across the FHS WHI, LBC 1921 and LBC 1936 cohort. Robust correlation coefficients (biweight midcorrelation) analysis were performed on the FHS and WHI cohort while generalized linear regression analysis adjusted for sex was performed on the LBC 1921 and 1936 cohort, respectively. For each variable, we display number of datasets, number of total subjects, the robust correlation results from the meta-analysis, FHS, and the WHI cohort and the Z statistics for the LBC respectively. The meta-analysis was based on Stouffer’s method for the majority of the variables or fixed effect models. The 2-color scale (blue to red) color-codes bicor correlation coefficients in the range [-1, 1] or Z statistics. The green color scale (light to dark) applied to unadjusted P values. Cell entry "--" denotes not available. The correlation analysis results stratified by sex using the FHS cohort are listed in Supplementary Figure 11 and stratified by ethnic group using the WHI cohort are listed in Supplementary Figure 12, respectively.

To assess the effect of life-style factors and diet on DNAmTLadjAge in blood, we meta-analyzed large data sets from the FHS and WHI cohort (N up to 6,977, Methods) including their associations with clinical measurements. Age-adjusted DNAmTL was positively correlated with plasma-based estimates of mean carotenoid levels (robust correlation r=0.10, unadjusted p=1.5E-5), beta-Cryptoxanthin (r=0.10 and p=3.8E-6) and high-density lipoprotein (HDL, r=0.04 and p=1.3E-3) (Figure 5, Supplementary Figures 11 and 12). The positive correlation between DNAmTLadjAge and self-reported measures of carotene intake (p=4.0E-3, N=766 from the Lothian Birth Cohort from the year 1936) was consistent with these findings. Positive associations with DNAmTLadjAge were also observed for self-reported measures of fruit (p=3.1E-5), vegetable (p=1.2E-3), dairy (p=7.1E-3), fish (P=0.018), and carbohydrate consumption (p=0.02). Positive correlations were also evident between DNAmTLadjAge and socio-economic factors such as level of educational attainment (p=3.3E-8) and income (p=3.1E-5). These associations held for each sex separately in the FHS cohort (Supplementary Figure 11).

There were also features that correlated negatively with DNAmTL. Smoking was strongly associated with lower DNAmTL values in leukocytes (p=2.3E-16) and in adipose tissue (P=0.036, Supplementary Figure 3D). C-reactive protein (p=4.3E-9), triglyceride levels (p=3.2E-4) and insulin levels (p=3.5E-4) were negatively correlated with DNAmTLadjAge in both FHS and WHI cohorts. There were also negative correlations of DNAmTLadjAge (in leukocytes) with waist-to-hip ratio (p=2.4E-3), body-mass index (BMI, p=3.6E-3) and physical exercise (p=0.02).

We caution the reader that our p-values are not adjusted for multiple comparisons.

### DNAmTL of leukocytes exhibits stronger association with smoking than does measured LTL

Next, we used 4,039 subjects from our seven validation cohorts to interrogate the impact of smoking on telomere shortening in leukocytes (Methods). A detailed smoking history (smoking pack-years) was known for roughly half of these individuals (N=2216). The smoking variable was based on pack-years when available otherwise based on never versus ever smoking. We adjusted the smoking variable for potential confounders (age, sex and ethnicity) of the relationship with LTL. Our large-scale meta-analysis showed that DNAmTL greatly outperformed measured LTL (Stouffer’s meta p=1.2E-17 versus meta p=0.029) with regards to association with smoking (Table 3). According to fixed effect models analysis (Methods), we found DNAmTL was shortened by 0.022±0.0002 kilobases per smoking pack-year (P=5.9E-07) while TL was not significantly lengthened by 1.3E-04±5.0E-04 kilobases per smoking pack-year (P=0.79). In an analogous analysis, we found that smokers had significantly shorter DNAmTL by 0.063±0.009 kilobases associated with a very robust P value (3.2E-13). The smokers also exhibited shorter TL (0.01±0.003 kilobases) but with far less significant P value (7.5E-04). Focusing on individual cohorts corroborated these findings, e.g. the association of smoking with DNAmTL (p=1.70E-11) was significantly greater than the association of smoking with LTL (p=6.77E-2, Table 3) in the BHS cohort. We also carried out a sensitivity analysis that compared age-adjusted LTL between current smokers and former/never smokers in the BHS and subgroups defined by sex and ethnicity. Our sensitivity analysis confirmed that smoking was indeed significantly associated with shorter age-adjusted DNAmTL in each subgroup (1.95E-8 ≤ p ≤ 4.39E-3, Supplementary Figure 13). Conversely, no significant associations were observed between LTL and smoking in these subgroups (0.12 ≤ p ≤ 0.87, Supplementary Figure 14).

**Table 3:** Smoking impacting on DNA methylation-based telomere length.

			**DNAmTL**			**LTL**	
**Cohort**	**N**	**Variable**	**T**	**P**		**T**	**P**
FHS^1^	878	Pack years	-2.08	3.81E-2		0.54	0.59
WHI BA23^1^	97	Pack years	0.45	6.53E-1		1.30	0.2
JHS^1^	100	Smoker	-3.00	3.38E-3		-1.87	6.40E-2
LBC 1921^2^	404	Pack years	-3.53	4.59E-4		-0.92	0.36
LBC 1936^2^	796	Pack years	-3.55	4.04E-4		0.55	0.58
UK Twin^2^	792	Smoker	-2.79	5.23E-3		-3.23	1.23E-3
BHS^1^	831	Smoker	-6.83	1.70E-11		-1.83	6.77E-2
**ALL**	**4039**	**Smoking impact**	**-8.55**	**1.21E-17**		**-2.19**	**0.029**

The Smoker variable was coded as a binary variable for never versus ever smokers, where estimate was referred to ever smokers.

^1^Telomere length was based on terminal restriction fraction measurement by Southern blotting.

^2^Telomere length was measured using quantitative real-time polymerase chain reaction (qPCR).

The table summarized the meta-analysis for the smoking impact on plasma DNA methylation based telomere length (DNAm TL) versus the impact on qPCR or southern blot-based leukocyte TL. The association tests were performed at each study cohort respectively then combined by the Stouffer’s method weighted by square root of sample size. The smoking variable was based on a binary variable for former/current smokers versus never or pack years provided the variable was available.

### Omega 3 intake is associated with longer DNAmTL

We were interested in investigating the relationship between DNAmTL and omega-3 polyunsaturated fatty acid (PUFA) supplementation in a large observational cohort study. We found omega-3 supplement intake to correlate positively with age-adjusted DNAmTL (bicor r=0.088 and p=4.4E-5), and this correlation was far more significant than that exhibited by age-adjusted LTL (bicor r=0.085 and p=0.016, Supplementary Figure 15). For DNAmTLadjAge, the effect of omega 3 supplementation was more pronounced in males (r=0.08, p=0.012) than in females (r=0.047, p=0.11). A multivariate linear mixed effects model analysis resulted in suggestive evidence (p=0.09) that omega-3 intake is associated with longer DNAmTLadjAge even after adjusting for sex, BMI, educational levels, and smoking pack year.

### DNAmTL relates to imputed blood cell composition

LTL is known to correlate with the abundance of naïve CD8+ T cells and other cell types [8]. Similarly, we found DNAmTL to be significantly correlated with several quantitative measures of leukocytes that were imputed using DNAm data (Methods) such as naïve CD8+ T cells (r=0.42, p=2.2E-151) and exhausted CD8+ T cells (r=-0.36, p=3.0E-102; Supplementary Figure 16 and Supplementary Data 2). A multivariate regression model revealed that 25% of the variation of age-adjusted DNAmTL and 4.6% of the variation in age-adjusted LTL could be attributed to imputed leukocyte cell composition in the FHS test data. Overall, DNAmTL exhibited substantially stronger correlations with imputed leukocyte cell composition than did LTL (Supplementary Figure 16 and Supplementary Data 2).

### DNAmTL CpGs tend to be located near telomeres and enriched with *cis*-mQTL

To test if the 140 CpGs of DNAmTL model were enriched at regions near telomeres, we performed hypergeometric tests based on the regions threshold up to 3 Mb (Methods). Our analysis showed that the 140 CpGs had moderate enrichment (hypergeometric p=8.1E-3, Supplementary Table 6) within sub-telomeric regions. Sensitivity analysis based on different thresholds validated the result (p=5.9E-3 ≤ P ≤ 0.039, Supplementary Table 6). Furthermore, we tested whether the 140 CpG sites overlapped with 52,916 *cis* methylation quantitative trait loci (*cis*-mQTL [35], Methods). Strikingly, 51 out of the 140 CpGs markers were known *cis*-mQTL (hypergeometric p=2.6E-15, Supplementary Data 1) which is consistent with the high heritability observed for DNAmTL (additive h^2^=0.46). Several of the 51 SNPs (from *cis*-mQTL) were implicated in complex traits according to GWAS data bases (Methods), e.g. rs2147904 in 1p34.2 (P ≥ 1.0E-14), rs305082 in 16q24.1 (P ≥8.0E-131) and rs945631 in 1p22.1 (P=2.0E-8) are known to be associated with granulocyte composition in blood. Further, rs945631 is also implicated in circulating phospholipid trans fatty acids at a suggestive P value=3.0E-06 (Supplementary Data 3). Bipolar disorder (P ≥ 2.0E-09) was identified when we inspected the SNPs in linkage disequilibrium with the 51 markers (Methods and Supplementary Data 3).

### DNAmTL and LTL exhibits different patterns of SNP associations

Alternatively, we briefly examined SNP association of DNAmTL across 10 loci implicated in measured LTL with genome-wide significance [36–38] (Methods). We previously found that SNPs in the *TERT* locus to be associated with epigenetic aging rates [19]. Inspecting the overlap regions between the 10 loci and the 140 CpGs (in DNAmTL, Supplementary Data 1), cg00580497 is located nearby the right arm of the *TERT* locus (within 500kb) and cg02282640 is located nearby the right arm of another highlighted gene *MPHOSPH6* [38]. However, using the FHS cohort, we did not find these SNPs or those at other loci to exhibit any significant associations (P<0.05) with DNAmLTLadjAge (Supplementary Table 7).

### Functional annotation of CpGs implicated in DNAmTL

We analyzed the genomic locations of the 140 CpGs underlying the DNAmTL using the GREAT software tool which assigns potential biological meaning to a set of genomic locations (here CpGs) by analyzing the annotations of nearby genes. Ten gene sets were identified as significant at a stringent Bonferroni corrected significance level (p<0.05) including “Cadherin, N-terminal” that mediate cell adhesion, polarization and migration (nominal p=1.0E-7) and SODD/TNFR1 signaling pathway (nominal p=5.2E-5, Supplementary Data 4). When focusing only on the subset of 72 CpGs that have negative coefficients in the DNAmTL model (increasingly de-methylated with telomere shortening), we found 10 statistically significant gene sets at a Bonferroni corrected p<0.05 including calcium-dependent adhesion and “Cadherin, N-terminal” (p=2.8E-10). When focusing only on the 68 CpGs with positive coefficients in the DNAmTL model we identified 4 gene sets at a Bonferroni corrected p<0.05 including the vascular endothelial growth factor (VEGF) signaling pathway (p=7.4E-5). The expression of these gene sets remains to be validated, and for moment their identities do not immediately proffer obvious clues as to how they may be linked with telomere attrition, making their involvement all the more intriguing.

### DNAmTL is often inferior to epigenetic clocks

Numerous lines of evidence suggest that telomere attrition and epigenetic aging are distinct cellular features that are well-associated with the aging process. As such both TL and epigenetic clocks are potential estimators of biological age [9]. To ascertain how related they are to each other, we calculated, pairwise correlations between DNAmTLadjAge and four “age-independent” measures of epigenetic age acceleration derived from (1) the pan-tissue epigenetic clock by Horvath (2013) [12] (2) the blood-based clock by Hannum et al. (2013) [11], (3) the DNAm PhenoAge estimator by Levine et al. (2018) [15], and (4) the DNAm GrimAge estimator by Lu et al. (2019) [17]. Using N=2356 samples from the FHS, we found that DNAmTLadjAge exhibited moderate negative correlations with the four epigenetic age acceleration measures (-0.44 ≤r ≤-0.20, Supplementary Figure 17). We then compared the performance between DNAmTL and epigenetic clocks in predicting health outcomes. Using the validation data (N=6,850), we found that DNAmTLadjAge rivals measures of age acceleration based on Hannum’s [11] or Horvath’s clock [12]. but was clearly inferior to age-adjusted DNAm PhenoAge [15] or DNAm GrimAge [17]. In particular, we demonstrated that AgeAccelGrim (based on DNAm GrimAge) greatly outperformed DNAmTLadjAge with regards to predicting time-to-death (Cox P=5.8E-71 for AgeAccelGrim versus p=4.1E-15 for DNAmTLadjAge), time-to-CHD (p=2.5E-23 for AgeAccelGrim versus p=6.6E-5), and time-to-CHF (p=1.8E-35 versus p=3.6E-6, Supplementary Figure 18).

### DNAmTL applied to *in vitro* cultured cells

**Figure 6:**
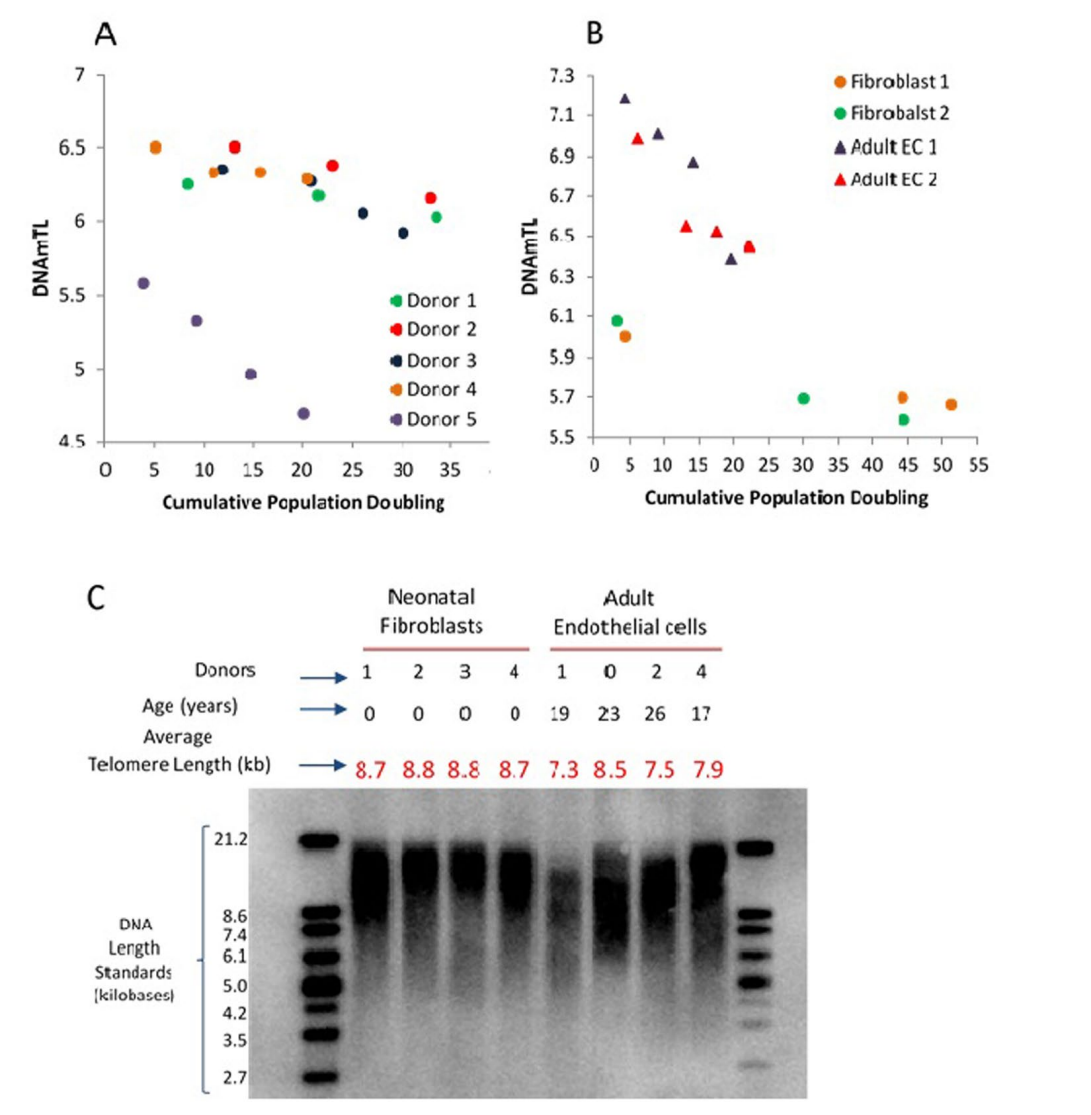
**Application of DNAmTL on *in vitro* keratinocytes, neonatal fibroblasts and adult coronary artery endothelial cells.** Panel (**A**) depicts decreasing DNAmTL values of keratinocytes from five heathy donors as a function of cumulative population doubling (y-axis, in units in kilobase). Panel (**B**) show that DNAmTL values of neonatal fibroblasts are smaller than those of adult coronary artery endothelial cells (EC). Both cell types exhibit decreasing DNAmTL in function of cumulative population doubling (x-axis). Panel (**C**) is the average telomere length measurement of neonatal fibroblasts and adult endothelial cells, which revealed that the telomeres of the former are longer than those of the latter.

**Figure 7:**
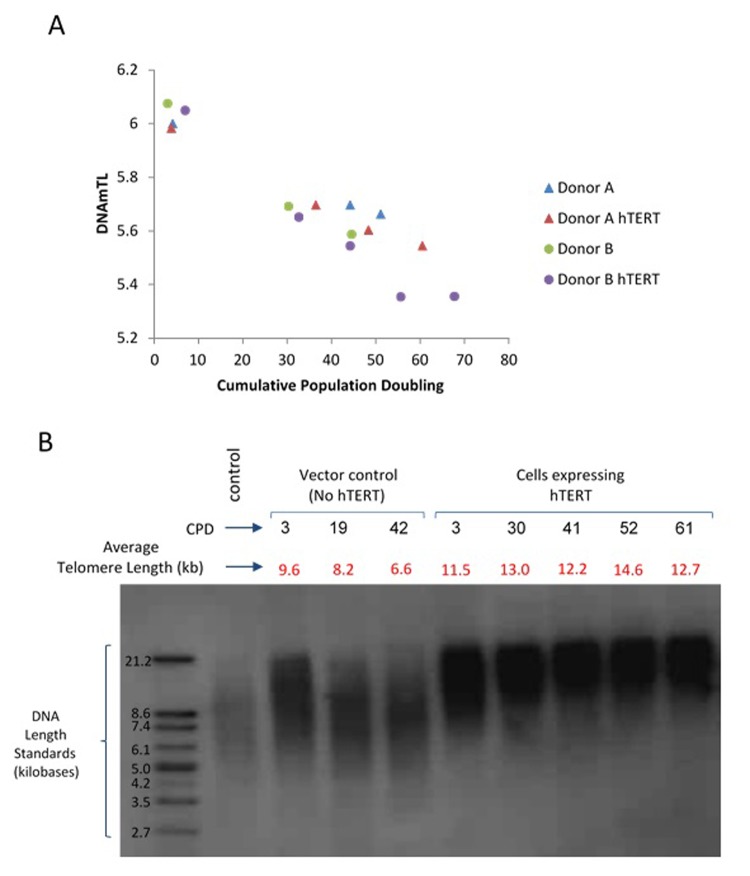
**Application of DNAmTL on hTERT-transduced primary human fibroblasts.** Panel (**A**) DNAmTL of primary neonatal fibroblasts without (Donor A and B) or with hTERT (Donor A hTERT and Donor B hTERT) transduction demonstrated linear and continued decrease in value regardless of hTERT status. This contrasts with average telomere length measurement by TRF southern blotting (Panel **B**), which revealed substantial increase in telomere length of primary neonatal fibroblasts that were transduced with hTERT-expression vector.

All the above analyses have shown that associations of several human traits and health outcomes with DNAmTL were much stronger than with LTL. What then might be the biological meaning of DNAmTL? We examined this question by monitoring DNAmTL in cultured somatic cells without and with expressed telomerase activity. Primary keratinocytes isolated from healthy human skin were grown with serial passaging upon confluence, where cell numbers were obtained, and their population doublings determined. DNA methylation profiles of cells after different population doublings were measured using Illumina EPIC array. DNAmTL was correlated with population doubling in both telomerase- negative and telomerase-positive cells, which displayed no telomere shortening (Figure 6 and Figure 7). Collectively, these findings suggest that DNAmTL records cell replication rather than TL. In the following, we provide more details. Primary keratinocytes isolated from healthy human skin were grown with serial passaging upon confluence, where cell numbers were obtained, and their population doublings determined. DNA methylation profiles of cells after different population doublings were measured using Illumina EPIC array. As would be expected, DNAmTL of keratinocytes from five heathy donors reduced in function of cumulative population doubling (Figure 6A). Accordingly, while the DNAmTL values of neonatal donors 1 to 4 were mutually comparable, that of donor 5, which are keratinocytes from a 65 years-old donor was markedly smaller, which is in keeping with established telomere biology. However, when neonatal fibroblasts and adult coronary endothelial cells (EC) were compared, the DNAmTL values of adult EC were greater than those of neonatal fibroblasts **(**Figure 6B**)**. Possible tissue-specific influence on telomere length was ruled out by TRF southern blot analyses which revealed that telomeres of neonatal fibroblasts were indeed longer than those of adult endothelial cells **(**Figure 6C**)**. This challenges the notion that DNAmTL is a surrogate for telomere length in non-blood tissue. If it were, then DNAmTL of primary cells that are transduced with hTERT should be augmented since this enzyme replicates and stabilizes telomeres. The results in Figure 7A show that this was not the case, as DNAmTL values of primary human fibroblasts continued to decrease even though they became immortalized, and TRF Southern blotting **(**Figure 7B**)** confirmed that their telomeres were indeed significantly increased and stabilized after the introduction of hTERT. Collectively, these studies show that DNAmTL is not the same as telomere length (especially when it comes to in vitro studies), hence indicating that it is instead, a DNA methylation-based surrogate for biological outcomes that are linked to telomere length in blood samples from adults.

## DISCUSSION

Considerable technical challenges are inherent in the process of measuring telomere length [39-41]. Our study was motivated by the recent insight that machine-learning methods (such as elastic net) can be used to develop remarkably robust DNAm based estimators of chronological age and mortality risk [12,14,15,17]. This robustness is due as much to the mathematical prowess of machine learning, as to the nature of the biomolecule that is measured, namely methylated DNA. Previously reported telomere-associated changes of methylation at sub-telomeric regions [26] raised the possibility that these changes may be predictive of telomere length.

Our overall goals were a) to test whether an optimal pattern of DNAm associated with TL, i.e., DNAmTL, captures associations with features of human aging-related traits and behaviors, and b) to gain a better understanding of the biological meaning of such associations. Using independent test data, we show that DNAmTL exhibits moderately strong correlations with measured TL (based on Southern blotting or qPCR) in blood and adipose tissue of individuals of different racial groups.

Since leukocyte DNAmTL is based on accurate measurements of methylated CpG, it is possible that its associations with LTL-related traits might actually be more robust than mean LTL. Indeed, we found that DNAmTL outperformed LTL (based on Southern blotting or qPCR) in detecting association with age, sex, ethnicity, lifestyle factors (diet, smoking, education, body mass index), and several clinical biomarkers (lipid levels, insulin). In addition, DNAmTL had a better predictive power than LTL for time-to-death and time-to coronary heart disease or heart failure. DNAmTL was also associated with physical functioning, age-at-menopause and diet (vegetable consumption, omega 3 intake) in the following direction: longer DNAmTL was associated with good health behavior and practices.

In addition to accurate measurement of methylated CpG, there might be another explanation for these associations and their biological meaning. Our studies in cultured cells with or without telomerase indicate that DNAmTL changes in function of cell replication independently of telomere attrition. Hence DNAmTL might be a read-out of cellular proliferation. This would be consistent with the better ability of leukocyte DNAmTL than LTL to capture associations with human traits and exposures that likely increase the turnover of hematopoietic stem/progenitor cells (HSPCs), which is the main determinant of LTL shortening [42–44]. Given the wide inter-individual LTL variation at birth [45], at any age, LTL reflects not only the accruing number of HSPC replication but also LTL at birth. Moreover, HSPC do have some telomerase activity [46], which is likely variable across individuals; therefore, HSPC TL shortening per replication might differ across individuals. The joint effect of inter-individual variations in LTL at birth and the amount of LTL shortening per HSPC replication thereafter might confound the ability of LTL to serve as an index of HSPC replication. This, however, does not apply to DNAmTL, as it is independent of telomerase activity.

Our results demonstrate that DNAmTL provides an attractive alternative to measured average TL when it comes to predicting health outcomes. The superiority of DNAmTL over measured LTL when it comes to determining the effects of modifiable behavior (e.g., smoking, BMI), is clearly an important feature that make DNAmTL a useful tool in seeking behavioral interventions that support healthy aging. The view that DNAmTL captures biologically relevant variation is also supported by our study of blood cell counts where DNAmTLadjAge is more strongly related to widely used biomarkers of immune-senescence (naive and exhausted cytotoxic T cells) than is measured LTL.

Although the training data were based on leukocyte DNA methylation profiles, we show that DNAmTL is applicable to other tissues as well (e.g. liver, adipose, and sorted monocytes). The general applicability of DNAmTL is important, if it were to be a powerful and robust tool. Similar extrapolation of DNAmTL is seen with its applicability to the entire age-span, despite the fact that the training data were based on adults (22-94 years old).

Having outlined the strengths of the analyses, we wish to acknowledge several limitations and how they were mitigated. First, the training and validation data used in the development of DNAmTL differed in terms of the underlying ethnic composition. However, subsequent analyses demonstrated that DNAmTL applies to all groups – indicating yet again that DNAmTL has successfully captured the underlying biological principle associated with LTL. Second, the DNA methylation data and LTL measurements in the Framingham test data were collected at different time points as described above. Nevertheless, the conclusions derived from those particular assessments were confirmed with analyses of a subset where both LTL measurements and DNAmTL were carried out with the same DNA samples. Importantly, we validated the significant correlation between DNAmTL and LTL in additional cohorts (Table 1). Third, our training data focused only on blood samples. We have demonstrated, however, that DNAmTL also applies to adipose and liver tissue, and we have also *in vitro* evidence that it also applies to keratinocytes and fibroblasts. It is to be noted that the unit of DNAmTL is retained as kilobases even though our in vitro studies demonstrate that its impressive correlation with TL notwithstanding, DNAmTL does not estimate actual telomere length. The preservation of the kilobase unit is purely on the basis that DNAmTL was trained using telomeres that are measured in these units.

While one of the 140 CpGs underlying DNAmTL is located near the TERT locus, it remains to be seen how these CpGs relate to telomere biology. The moderate enrichment of the DNAmTL CpGs within regions proximal to telomeres is consistent with previous reports of TL-associated changes in sub-telomeric methylation levels [26]. Our *cis* mQTL study revealed 51 neighboring SNPs. Several of these SNPs are related to blood cell composition (e.g. granulocytes) and one SNP was found to be associated with bipolar disorder, which might be linked to LTL [47,48].

We initiated this investigation, leveraging experience gained from our work with epigenetic clocks [12,14,15,17], which we have used in past research to demonstrate the difference between telomere attrition and epigenetic aging [49]. Our present findings support this conclusion. The epigenetic clocks and DNAmTL do not share CpGs and the respective genes proximal to their CpGs also do not seem to overlap in function. For example, the 140 CpGs underlying DNAmTL tend to be located near cadherin and cell signaling genes while other functional categories were implicated by epigenetic clocks. We find that DNAmTL is associated with the four epigenetic clocks in the expected way, which is that low values of DNAmTLadjAge correspond to high epigenetic age acceleration. Comparative analysis of 3 large cohorts revealed that DNAmTL is in general inferior to epigenetic clocks (especially DNAm GrimAge [17]) in predicting lifespan and other age-related traits. This is unsurprising as DNAm GrimAge was trained using lifespan data. The DNAmTL is nonetheless, an important epigenetic biomarker because it might provide a mechanistic link between cell replication and aging-related diseases and environmental exposures. Notwithstanding the clear difference between telomere-associated aging and epigenetic aging, there is a moderate level of association between DNAmTL and epigenetic clocks, which is to be expected as they (directly or indirectly) relate to age-related DNAm changes, distinctiveness aside [50].

The successful transition of telomere-associated aging into a methylation-based assay allows one to use a single platform (e.g. the Illumina methylation array) to measure two distinct mechanisms of aging - epigenetic aging and telomere-associated aging.

Like epigenetic clocks, we expect that DNAmTL will become a useful biomarker in human interventional studies. A proof-of-concept study is provided by our preliminary analysis of omega-3 polyunsaturated fatty acid (PUFAs) supplementation. Several large-scale studies failed to detect convincing association between omega-3 PUFA supplementation and risk of cardiac death, sudden death, myocardial infarction, stroke, or all-cause mortality [51–53]. However, we find omega-3 intake to be positively correlated with DNAmTLadjAge (robust r=0.088 and P=4.4E-5) and with age-adjusted TRF-based LTL (robust r=0.085 and P=0.016, Supplementary Figure 15). Future randomized controlled trials should aim to validate these associations. Overall, we expect that DNAmTL will become an attractive molecular biomarker of aging due to its greater sensitivity to age related conditions than measured TL, its ease of use and robustness.

## MATERIALS AND METHODS

### Epidemiological cohorts

To establish DNAm-based telomere length in leukocytes we used N=2256 individuals from the WHI BA23 and JHS cohort in the training process and N=1078 individuals from the FHS cohort, WHI [54,55], JHS [56] cohort in the test process, as listed in Table 1. More details of the study cohorts are described in Supplementary Note 1.

Our validation analyses involved N=9,785 Illumina Infinium 450k or EPIC 850k arrays measuring blood methylation levels in N=9,345 individuals from seven independent cohorts across nine studies: the FHS dataset (N=2,356), WHI BA23 (N=1,389), WHI EMPC (N=1972), JHS (N=209), InChianti (N=924 from 1 to 2 longitudinal measures on 484 individuals), Lothian Birth Cohort of 1921 (N=404) and 1946 (N=906), Twins UK (N=794) and Bogalusa Heart Study (N=831, Table 1, Supplementary Table 1, and Supplementary Note 1). All statistical analyses were adjusted for the correlation structure due to pedigree effects or repeated measurements as described below.

### LTL measurements in training and test datasets

The same lab generated the LTL data across the 3 cohorts [25]. LTL was measured using Southern blots of the terminal restriction fragment length. After extraction, DNA was inspected for integrity, digested, resolved by gel electrophoresis, transferred to a membrane, hybridized with labeled probes and exposed to X-ray film using chemiluminescence, as previously described in [25]. The inter-assay coefficient of variation for blinded pair sets was 2.0% for the WHI, 1.4% for the JHS and 2.4% for the FHS [25].

### Average telomere length measurement of *in vitro* cultured cells

DNA from primary human fibroblasts were extracted according to the protocol provided by Zymo Research (USA) using the mini-prep kit (Cat No: D4004). 1.5 micrograms of DNA were digested with Hinf I and RsaI prior to being resolved through a 0.8% agarose gel. DNA was transferred to Hybond N+ nylon membrane by Southern transfer, after which it was baked for 20 minutes 120^o^C. The subsequent steps of this process are as described in the protocol provided by TeloTAGGG (Cat No:12209136001, Sigma Aldrich, USA). Chemiluminescence signal was captured using a Kodak Gel Documentation apparatus and quantified using Kodak quantification software. Average telomere length was ascertained using the formula described within the protocol provided.

### Transduction of primary human fibroblasts with hTERT

Primary human fibroblasts were isolated from neonatal foreskin and transduced with hTERT using recombinant retroviruses according to methods previously described [49].

### Estimation of surrogate DNAm based telomere length in leukocytes

We developed an estimate of LTL based only on DNA methylation levels. The estimate was established using the elastic net regression model implemented in the R package *glmnet* [51]. The elastic net regression model corresponds to a choice of 0.5 for the alpha parameter in the *glmnet* function. Ten-fold cross validation was performed in the (WHI and JHS) training data to specify the underlying tuning parameter λ. The final model was based on *lambda.1se*, i.e., the λ value that led to the minimum cross validated error within one standard error.

### DNAmTL applied to different blood cell types

To evaluate how DNAmTL (and epigenetic clocks such as DNAmAge [12], and DNAmAgeSkinBlood [14]) differ across different blood cell types, we analyzed DNA methylation data from sorted blood cells from 6 men aged from 27 and 32 years old. DNA methylation profiles were generated from peripheral blood mononuclear cells (PBMC) and single cell types: CD4+T, CD8+T, and B cells were measured using the Illumina Infinium 450k platform.

### DNA methylation data

The DNA methylation profiling was based on the Illumina Infinium HumanMethylation450K BeadChip in the FHS and WHI cohort and was based on the Illumina Infinium EPIC 850K BeadChip in the JHS cohort. To ensure future use with EPIC arrays, we focused on the subset of 450,161 CpGs that were present on both platforms. We kept the original normalization methods to ensure consistency with previous publications. The WHI BA23 were normalized using the background correction method implemented in the software *GenomeStudio*. WHI EMPC were normalized based on BMIQ [57] for beta-mixture quantile normalization. The JHS data were normalized using the "noob" normalization method implemented in the *minfi* R package [58,59].

### Statistical models used in validation analysis

Our validation analysis involved several regression models. Cox regression for various censored outcomes such as time to death (all-cause mortality), time-to-CHD, time-to-CHF, and time to any cancer. Multivariate linear regression for our DNAm based measures (independent variable) associated with and number of age-related conditions (dependent variable) and physical function score, respectively. Linear regression was used to relate age at menopause (independent variable) with DNAmTL. Logistic regression analysis for binary outcomes allowing us to estimate odds ratios for the binary variables such as cancer status, hypertension, type 2 diabetes, and disease-free status. The multimorbidity index was defined as the number of age-related conditions including arthritis, cataract, cancer, CHD, CHF, emphysema, glaucoma, lipid condition, osteoporosis, type 2 diabetes (see Supplementary Note 1). In our validation analysis, we used the age-adjusted variable, DNAmTLadjAge, which is not correlated with chronological age. All regression models included the following covariates: chronological age, sex, and batch effect as needed. To avoid bias due to familial correlations from pedigrees in the FHS cohort or intra-subject correlations resulting from repeated measurements, we used the following techniques. For censored time variables, we used robust standard errors, the Huber sandwich estimator, implemented in the R function "*coxph*". We used linear mixed models with a random intercept term, implemented in the R function *lme*. We used generalized estimation equation models (GEE), implemented in the *gee* function, for our logistic regression models. Additional covariates related to demographic characteristics, psychosocial behaviors and clinical covariates were adjusted in multivariate Cox models analysis. Those additional covariates include BMI, educational attainment (category), alcohol consumption (gram/day), self-reported smoking pack-years, three medical covariates: status of (any) cancer, hypertension and type 2 diabetes at baseline. BMI was categorized into 3 groups: 18.5 -24.9 (normal), b) 25 to 29.9 (overweight), and c) >=30 (obese). The categories associated with educational attainment were a) less than high school, b) high school degree, c) some college, and d) college degree and above. Smoking pack-years and educational variables were not available in the JHS cohort. Smoking status (never, former and current) was used in the analysis of the JHS cohort. Our stratified analysis was conducted in strata defined by age (<65 versus ≥ 65 years), BMI, education, prior history of hypertension, type 2 diabetes or cancer. All models used in the stratified analysis adjusted for age, sex, and (possibly) batch effect.

### Meta-analysis

We mainly used fixed effects meta-analysis models weighted by inverse variance to combine the results across validation study sets into a single estimate. Toward this end, we used the *metafor* R function. Alternatively, we used Stouffer’s meta-analysis method (weighted by the square root of sample size) to combine results for variables whose scale/definition differed across study sets, e.g. multimorbidity (number of age-related conditions), disease-free status and physical function scores.

### Cox models that include blood cell counts

We also fit multivariate Cox regression models that adjusted for imputed blood cell counts in addition to chronological age, batch, and pedigree structure, for predicting time-to-death and time-to-CHD. The blood cell counts were imputed based on DNA methylation levels as described elsewhere [60,61]. To avoid multi-collinearities between blood cell counts, we only included the following seven blood cell counts into the multivariate model: naïve CD8+T, exhausted cytotoxic CD8+ T cells, plasma blasts, CD4+T, natural killer cells, monocytes and granulocytes.

### Heritability analysis

In general, DNAm based biomarkers are highly heritable [19,62,63]. To evaluate whether DNAmTLadjAge is heritable as well, we estimated the narrow sense heritability $h^{2}$ using the polygenic models defined in SOLAR and its R interface solarius [64]. Heritability is defined as the total proportion of phenotypic variance attributable to genetic variation in the polygenic model. The quantitative trait DNAmTL was adjusted for both age and sex. The robust polygenic model (with the option of a t-distribution) was used to estimate heritability. The heritability estimate correspondents to the variance component associated with the kinship coefficient. We used all individuals from the FHS cohort for whom DNA methylation data were available (irrespective of the availability of the observed LTL measure).

### SNP associations of DNAmTL

We performed 14 SNP associations across 10 distinct susceptibility loci associated with LTL reported from three large-scale studies: (I) meta-analysis association of LTL in chromosome 5 *TERT* only (N=53,724) [36], (II) a genome-wide meta-analysis of LTL (N=37,684) [37], or (III) a genome-wide meta-analysis of LTL (N=26,089) [38]. SNP associations were performed for DNAmTLadjAge and LTLadjAge respectively, using the individuals of the FHS cohort available for both measures (N=811). The association analysis was based on linear mixed models whose random covariance matrix was determined by kinship coefficients of the pedigree structure, adjusted for sex and three principal components as fixed effects. We conducted the association tests using the function "relmatLmer" from the R library "lme4qt".

### LTL measures versus blood cell composition

The imputed blood cell abundance measures were related to TRF based and DNAm based LTL measures, using the training datasets from the WHI BA23 and the JHS cohort and the test dataset from the FHS cohort, involving n=3,134 individuals. The following imputed blood cell counts were analyzed: B cell, naïve CD4+ T, CD4+ T, naïve CD8+ T, CD8+ T, exhausted cytotoxic CD8+ T cells (defined as CD8 positive CD28 negative CD45R negative), plasma blasts, natural killer cells, monocytes, and granulocytes. The blood cell composition imputation of the naive T cells, exhausted T cells, and plasma blasts was based on the Horvath method [65]. The remaining cell types were imputed using the Houseman method [61]. More details are described in Supplementary Methods. To avoid confounded by age, we used the age-adjusted DNAmTL (DNAmTLadjAge) variable for analysis. The correlation results were combined across studies via the same fixed effects meta-analysis model.

### GREAT analysis

We applied the GREAT analysis software tool to three sets of CpGs: (1) all the 140 CpGs underlying DNAmTL model, (2) the 72 CpGs with negative coefficients in the model, and (3) the other 68 CpGs with positive coefficients in the model. CpGs in non-coding regions typically lack annotation with respect to biological functions. GREAT assigns biological meaning to a set of non-coding genomic regions (implicated by the CpGs) by analyzing the annotations of the nearby genes. Toward this end, the GREAT software performs both a binomial test (over genomic regions) and a hypergeometric test over genes when using a whole genome background. We performed the enrichment based on default settings (Proximal: 5.0 kb upstream, 1.0 kb downstream, plus Distal: up to 1,000 kb) for gene sets associated with GO terms, MSigDB, PANTHER, KEGG and InterPro pathway. To avoid large numbers of multiple comparisons, we restricted the analysis to the gene sets with between 5 and 3,000 genes. We report nominal P values and two adjustments for multiple comparisons: Bonferroni correction and the Benjamini-Hochberg false discovery rate.

### Diet and lifestyle factors

We performed a robust correlation analysis (biweight midcorrelation, bicor) or generalized linear regression analysis between DNAmTLadjAge and 43 variables including 15 self-reported dietary variables, 9 dietary biomarkers, 14 variables related to metabolic related traits and central adiposity, and 5 life style factors, using the FHS, WHI, LBC 1921 and/or LBC 1936 cohort (N up to 6977), as listed in the first two columns in Figure 5. In the FHS cohort, we conducted the robust correlation analysis in males and females, respectively. Next we combined the results via fixed effect models weighted by inverse variance. In the FHS cohort, we used linear mixed effects models to account for pedigree structure. In the WHI cohort, we conducted the robust correlation analysis in each ethnic group separately. Next we combined the results via fixed effects meta-analysis models. In the LBC cohorts, we performed generalized linear regression analysis adjusted for sex for the 1921 and 1936 follow-up, respectively. Most of the final results were combined based on the Stouffer’s method weighted by the square root of sample size across the study cohorts. A few variables that were only available in both FHS and WHI cohort and presented in the same scaling of effect sizes were combined based on the fixed effect models weighted by inverse variance. In the FHS cohort, the diet and the clinical variables were based on the datasets archived in dbGAP pht002350.v4.p10 and pht000742.v5.p10, respectively. Both datasets were collected at exam 8, aligned with the blood drawn for DNA methylation profiles. In the WHI cohort, blood biomarkers were measured from fasting plasma collected at baseline. Food groups and nutrients are inclusive, including all types and all preparation methods, e.g. folic acid includes synthetic and natural, and dairy includes cheese and all types of milk. The individual variables are explained in [66].

### Interrogating smoking impact on telomere length in blood

We used the 4,039 subjects across seven studies from our validation cohorts to compare the smoking association with (1) DNAm TL and with (2) measured LTL. Multivariate linear regression analysis of telomere length on smoking was performed on each study, adjusted for chronological age and adjusted sex, ethnicity and pedigree effects as needed. Smoking information was based on pack years when available (N=2216) otherwise based on a binary variable (never smokers versus ever smokers). The Stouffer’s method weighted by square root of sample size was performed to combine the results across all studies. Fixed effect models weighted by inverse variance were applied to the studies with pack years information and the other studies only with the binary smoking variable, respectively.

### Enrichment analysis of CpGs in DNAmTL near telomeres

We performed hypergeometric analysis for evaluating the overlap between the 140 CpGs comprising our DNAm TL model and the CpGs nearby telomere regions from 3 mega base (Mb) at each chromosome tail. In the hypergeometric test, the margin for the total number of CpGs across whole genome was based on 453093 CpGs present in both 450k and Epic arrays. We also performed sensitivity analysis based on a variety of regions thresholded at 2, 4, 6 and 8 Mb respectively.

Enrichment analysis of CpGs in DNAmTL annotated with DNA methylation quantitative trait locus (mQTL), conducted in McRae et al. [35]. The hypergeometric test was used to evaluate the overlap of the 140 CpGs with 52915 cis-mQTL. The mQTL CpGs were identified using a stringent criterion described in the original reference: a cis-mQTL was identified either in the Brisbane Systems Genetics Study or in the Lothian Birth Cohorts at P < 1.0E-11 and was replicated in the other cohort at P < 1.0E-6.

The resulting 51 SNPs underlying cis-mQTL loci were cross referenced to GWAS Catalog tool (version v1.02 database, see URL). We not only report SNPs that met genome-wide (P < 5.0E-8) significance levels but also suggestive significance (P< 5.0E-6). Similarly, we characterized the 51 SNP using the HaploReg (version 4.1) tool [67] (see URL) which allows one to visualize SNPs in linkage disequilibrium (LD r^2^ ≥ 0.8 based on European ancestry).

### In vitro cultured cell procedure

#### Isolation and culture of primary cells

Primary human neonatal fibroblasts were isolated from circumcised foreskins. Informed consent was obtained prior to collection of human skin samples with approval from the Oxford Research Ethics Committee; reference 10/H0605/1. The tissue was cut into small pieces and digested overnight at 4 °C with 0.5 mg/ml Liberase DH in CnT-07 keratinocyte medium (CellnTech) supplemented with penicillin/streptomycin (Sigma) and gentamycin/amphotericin (Life Tech). Following digestion, the epidermis was peeled off from the tissue pieces. The de-epidermized tissue pieces were placed faced down on plastic cell culture plates and allowed to partially dry before addition of DMEM supplemented with 10% FBS and antibiotics. After several days incubation in a 37 °C, 5% CO_2_ humidified environment, fibroblasts can be seen to migrate out from the tissue pieces and when their growth reached confluence, they were trypsinized, counted and seeded into fresh plates for experiments. Adult human coronary artery endothelial cells (HCAEC) were purchased from Cell Applications (USA) and cultured in MesoEndo Cell Growth Medium (Sigma) at 37 °C humidified incubator with 5% CO_2_.

#### Neonatal foreskin fibroblasts

100,000 cells were seeded into a 10cm plate and cultured as described above. Upon confluence the cells were harvested with trypsin digestion followed by neutralization with soybean trypsin inhibitor. The number of cells was ascertained and 100,000 was taken and seeded into a fresh plate. The remaining cells were used for DNA extraction. Population doubling was calculated with the following formula: [log(number of cells harvested) – log(number of cells seeded)] x 3.32. Cumulative population doubling was obtained by addition of population doubling of each passage.

#### Adult human coronary artery endothelial cells

500,000 cells were seeded into a fibronectin-coated 75cm^2^ flask and cultured as described. The procedure of passing the cells, counting and ascertaining population doubling is similar to those described for neonatal foreskin fibroblasts above.

#### Retroviral-mediated transduction of cells with hTERT vectors

Retroviral vectors bearing wildtype hTERT (Addgene, cat. 1774), was transfected into Phoenix A cells using the calcium chloride method according to the manufacturer’s instructions (Profection Cat No: E1200 Promega). The next day, media were removed from the transfectants and replaced with DMEM supplemented with 10% foetal calf serum. The following day, the media containing recombinant retroviruses were collected, filtered through 0.45micron filter and mixed with polybrene (Sigma) up to 9ug/ml and used to feed the cells intended for infection. The next day, fresh media containing puromycin (1ug/ml) was given to the cells. After 3-4 days when all the control cells in the uninfected plates were dead, the surviving infectants were grown and used for experiments as described above.

#### DNA extraction in the in vitro experiments

DNA was extracted from cells using the Zymo Quick DNA mini-prep plus kit (D4069) according to the manufacturer’s instructions and DNA methylation levels were measured on Illumina 850 EPIC arrays according to the manufacturer’s instructions.

### URLs

GWASCatalog, https://www.ebi.ac.uk/gwas/search?query=28346442

HaploReg, http://www.broadinstitute.org/mammals/haploreg/haploreg.php

## Supplementary Material

Supplementary Note 1

Supplementary Note 2

Supplementary Methods

Supplementary Figures

Supplementary Tables

Supplementary References

Supplementary Data 1

Supplementary Data 2

Supplementary Data 3

Supplementary Data 4
