# Supplementary material for: DNA methylation-based estimator of telomere length
Source: Aging (Albany NY). 2019 Aug 18;11(16):5895–923. doi: 10.18632/aging.102173 (PMC6738410; doi:10.18632/aging.102173)
Supplement: Supplementary Note 1 [file aging-11-102173-s008.pdf]

SUPPLEMENTARY NOTE 1

Description of datasets in training, test and validation analysis

Our study involved (I) 3334 study participants in the training and test data sets that were used to develop and validate DNAmTL, and (II) 9345 participants (9875 blood samples) from 9 cohorts across 7 studies. The participated studies include (1) Framingham Heart Study (FHS) offspring cohort, (2) Women's Health Initiative (WHI), (3) Jackson Heart Study (JHS), (4) InChianti, (5) Twins UK, (6) Lothian Birth Cohorts (LBC), and (7) Bogalusa Heart Study (BHS). Here we briefly describe each study.

Framingham Heart Study Cohort

The FHS cohort [1] is a large-scale longitudinal study started in 1948, initially investigating the common factors of characteristics that contribute to cardio-vascular disease (CVD), https://www.framinghamheartstudy.org/index.php. The study at first enrolled participants living in the town of Framingham, Massachusetts, who were free of overt symptoms of CVD, heart attack or stroke at enrollment. In 1971, the study started FHS Offspring Cohort to enroll a second generation of the original participants' adult children and their spouses (n= 5124) for conducting similar examinations [2]. Participants from the FHS Offspring Cohort were eligible for our study if they attended both the seventh and eighth examination cycles and consented to having their molecular data used for study. We used the 2,356 participants from the group of Health/Medical/Biomedical (IRB, MDS) consent and available for both Immunoassay array DNA methylation array data. The FHS data are available in dbGaP (accession number: phs000363.v16.p10 and phs000724.v2.p9).

We computed the total of number age-related conditions based on dyslipidemia, hypertension, cardiovascular disease (including coronary heart disease [CHD] or congestive heart failure [CHF]), type 2 diabetes, cancer and arthritis. Time to CHD or time to CHF was truncated at zero if it occurred before exam 8. Deaths among the FHS participants that occurred prior to January 1, 2013 were ascertained using multiple strategies, including routine contact with participants for health history updates, surveillance at the local hospital and in obituaries of the local newspaper, and queries to the National Death Index. Death certificates, hospital and nursing home records prior to death, and autopsy reports were requested. When cause of death was undeterminable, the next of kin were interviewed. The date and cause of death were reviewed by an endpoint panel of 3 investigators.

DNA methylation quantification

Peripheral blood samples were collected at the 8th examination. Genomic DNA was extracted from buffy coat using the Gentra Puregene DNA extraction kit (Qiagen) and bisulfite converted using EZ DNA Methylation kit (Zymo Research Corporation). DNA methylation quantification was conducted in two laboratory batches using the Illumina Infinium HumanMethylation450 array (Illumina). Methylation beta values were generated using the Bioconductor *minfi* package with Noob background correction[3].

Women's Health Initiative

The WHI is a national study that enrolled postmenopausal women aged 50-79 years into the clinical trials (CT) or observational study (OS) cohorts between 1993 and 1998 [4, 5]. We included 4,079 WHI participants with available phenotype and DNA methylation array data: 2,107 women from "*Broad Agency Award 23*" (WHI BA23) and 1,972 women from "*Epigenetic Mechanisms of PM-Mediated CVD Risk*" (WHI EMPC). WHI BA23 focuses on identifying miRNA and genomic biomarkers of coronary heart disease (CHD), integrating the biomarkers into diagnostic and prognostic predictors of CHD and other related phenotypes, and other objectives can be found in https://www.whi.org/researchers/data/WHIStudies/StudySites/BA23/Pages/home.aspx. WHI EMPC is a study of epigenetic mechanisms underlying associations between ambient particulate matter (PM) air pollution and cardiovascular disease [6]. WHI EMPC and BA23 span three WHI sub-cohorts including GARNET, WHIMS and SHARe. 936 EMPC participants were not in any of the WHI GWAS (either GARNET, WHIMS, SHARe, MOPMAP, HIPFX, or GECCO). The largest overlap was with SHARE & GARNET. There was almost no overlap with WHIMS & MOPMAP.

The total number of age-related conditions was based on Alzheimer's disease, amyotrophic lateral sclerosis, arthritis, cancer, cataract, CVD, glaucoma, emphysema, hypertension, and osteoporosis.

DNA methylation quantification for BA23

In brief, bisulfite conversion using the Zymo EZ DNA Methylation Kit (Zymo Research, Orange, CA, USA) as well as subsequent hybridization of the Human-Methylation450k Bead Chip (Illumina, San Diego, CA), and scanning (iScan, Illumina) were performed according to the manufacturers protocols by applying standard settings. GenomeStudio was used for background correction. DNA methylation levels (β

values) were determined by calculating the ratio of intensities between methylated (signal A) and un-methylated (signal B) sites. Specifically, the β value was calculated from the intensity of the methylated (M corresponding to signal A) and un-methylated (U corresponding to signal B) sites, as the ratio of fluorescent signals $\beta = Max(M,0)/[Max(M,0)+Max(U,0)+100]$. Thus, β values range from 0 (completely un-methylated) to 1 (completely methylated).

DNA methylation quantification for WHI EMPC
Illumina Infinium HumanMethylation450 BeadChip data from the Northwestern University Genomics Core Facility for WHI EMPC participants sampled in stages 1a, 1b, and 2 were quality controlled, normalized and batch adjusted. Beta-mixture quantile normalization was implemented using BMIQ [7] and empirical Bayes methods of batch adjustment for stage and plate were implemented in ComBat [8].

Lifestyle factors and dietary assessment in the Women's Health Initiative (WHI)
WHI participants completed self-administered questionnaires at baseline which provided personal information on a wide range of topics, including sociodemographic information (age, education, race, income), and current health behaviors (recreational physical activity, tobacco and alcohol exposure, and diet). Participants also visited clinics at baseline where certified Clinical Center staff collected blood specimens and measured anthropometrics (weight, height, hip and waist circumferences) and blood pressures (systolic, diastolic). Body mass index and waist to hip ratio were calculated from these measurements.

Dietary intake was assessed at baseline using the WHI Food Frequency Questionnaire [9]. Briefly, participants were asked to report on dietary habits in the past three months, including intake, frequency, and portion sizes of foods or food groups, along with questions concerning topics such as food preparation practices and types of added fats. Nutrient intake levels were then estimated from these responses. For current drinker, we use the threshold of more than one serving equivalent (14g) within the last 28 days.

Jackson Heart Study

The JHS is a large, population-based observational study evaluating the etiology of cardiovascular, renal, and respiratory diseases among African Americans residing in the three counties (Hinds, Madison, and Rankin) that make up the Jackson, Mississippi metropolitan area [10] Data and biologic materials have been collected from 5306 participants, including a nested family cohort of 1,498 members of 264 families. The age at enrollment for the unrelated cohort was 35-84 years; the family cohort included related individuals >21 years old. Participants provided extensive medical and social history, had an array of physical and biochemical measurements and diagnostic procedures, and provided genomic DNA during a baseline examination (2000-2004) and two follow-up examinations (2005-2008 and 2009-2012). The study population is characterized by a high prevalence of diabetes, hypertension, obesity, and related disorders. Annual follow-up interviews and cohort surveillance are ongoing. In our analysis, we used the visits at baseline from 1747 individuals as part of project JHS ancillary study ASN0104, available with both phenotype and DNA methylation array data. Total numbers of age-related conditions were based on hypertension, type 2 diabetes, kidney dysfunction based on ever dialysis, and CVD.

DNA methylation quantification
Peripheral blood samples were collected at the baseline. Methylation beta values were generated using the Bioconductor *minfi* package with Noob background correction [3].

Invecchiare in Chianti, aging in the Chianti area (InChianti)

The InChianti (Invecchiare in Chianti, aging in the Chianti area) cohort is a representative population-based study of older persons enrolling individuals aged 20 years and older from two areas in the Chianti region of Tuscany, Italy, http://inchiantistudy.net/wp/ . One major goal of the study is to translate epidemiological research into geriatric clinical tools, ultimately advancing clinical applications in older persons. Of the cohort, 924 observations from 484 individuals with both phenotype information and DNA methylation data were including in our studies. The observations were collected from baseline in 1998 and the third follow-up visit in 2007. All participants provided written informed consent to participate in this study. The study complied with the Declaration of Helsinki. The Italian National Institute of Research and Care on Aging Institutional Review Board approved the study protocol. We computed the total number of age-related conditions based on cancer, hypertension, myocardial infarction, Parkinson's disease, stroke and type 2 diabetes.

DNA methylation quantification
Genomic DNA was extracted from buffy coat samples using an AutoGen Flex and quantified on a Nanodrop1000 spectrophotometer prior to bisulfite conversion. Genomic DNA was bisulfite converted using Zymo EZ-96 DNA Methylation Kit (Zymo

Research Corp., Irvine, CA) as per the manufacturer's protocol. CpG methylation status of 485,577 CpG sites was determined using the Illumina Infinium HumanMethylation450 BeadChip (Illumina Inc., San Diego, CA) as per the manufacturer's protocol and as previously described [11]. Initial data analysis was performed using GenomeStudio 2011.1 (Model M Version 1.9.0, Illumina Inc.). Threshold call rate for inclusion of samples was 95%. Quality control of sample handling included comparison of clinically reported sex versus sex of the same samples determined by analysis of methylation levels of CpG sites on the X chromosome[11]. Methylation beta values were generated using the Bioconductor *minfi* package with Noob background correction [3].

The Lothian Birth Cohorts (LBC)

The Lothian Birth Cohorts of 1921 (LBC21) and 1936 (LBC36) are ongoing longitudinal studies of ageing in individuals born in 1921 and 1936. At age 11, these individuals completed the Moray House Test of general intelligence as part of the Scottish Mental Surveys of 1932 and 1947, respectively. Decades later, individuals living in Edinburgh and the surrounding areas were contacted and invited to participate in the Lothian Birth Cohort (LBC) studies with the aim to study cognitive ageing. 550 individuals of those born in 1921 were recruited between 1999 and 2001 at mean age ~79. Of those born in 1936, 1091 individuals were recruited between 2004 and 2007 at mean age ~70. Since then, extensive phenotypic data including physical, cognitive, psychosocial and lifestyle measures was collected roughly every 3 years in 5 waves of testing. In addition, genetic and epigenetic profiling is available in both LBC21 and LBC36. More detail on recruitment and testing has been published elsewhere [12, 13]. Ethical approval for the first wave of Lothian Birth Cohort studies was obtained from the Multi-Centre Research Ethics Committee for Scotland (MREC/01/0/56) and the Lothian Research Ethics committee (LREC/1998/4/183; LREC/2003/2/29). All participants provided written informed consent.

Here, DNA-methylation data as well as a range of phenotypic measures obtained in wave 1 of testing in LBC21 and LBC36 are used.

Mortality

Information on the mortality status of participants in LBC1921 and LBC1936 was obtained by data linkage with the National Health Service Central Register, provided by National Records of Scotland. Of the 436 participants in LBC1921, 37 were alive at time of last censor. In LBC1936, 680 participants were alive at time of last censor.

Leukocyte Telomere length (LTL)

LTL in wave 1 of the LBC1921 and LBC1936 was measured using a quantitative real-time polymerase chain reaction (PCR) assay [14-16]. DNA was extracted from whole blood at the Wellcome Trust Clinical Research Facility Genetics Core (Western General Hospital, Edinburgh) using standard procedures. PCRs were performed using a 7900HT Fast Real Time PCR machine with 384-well plate capacity (Applied Biosystems; Pleasonton, CA, USA). LTL was measured as the ratio of telomeric template to glyceraldehyde 3-phosphate dehydrogenase. Cell lines of known absolute telomere length were included within each plate as internal control DNA samples to correct for plate-to-plate variation and measurements were performed in quadruplicate with the mean being used.

DNA methylation quantification

DNA-methylation was measured at 485,512 sites using the Illumina 450k methylation array. Raw DNA methylation IDAT files were read into R using the *minfi* package [3]. Data were normalised using the Noob method [17], implemented by the preprocessessNoob function in *minfi* which incorporates a background subtraction method and dye-bias normalization. Briefly, the background subtraction method estimates background noise from out-of-band probes and remove it for each distinct sample, whereas the dye-bias normalisation uses a subset of control probes to estimate the dye bias.

Lifestyle/Dietary Measures

Participants provided information on education and health behaviours, such as smoking and alcohol consumption, during a sociodemographic and medical history interview. Blood pressure, height and weight (from which Body mass Index (BMI) was calculated) were measured by trained research nurses during a clinical assessment. Blood samples collected at this assessment were used to measure A1C, CRP, triglycerides, cholesterol and creatinine levels. In addition, dietary data in LBC1936 were obtained using the Scottish Collaborative Group Food frequency questionnaire version 7.0 (http://www.foodfrequency.org.uk) [18]. An estimation of dairy consumption was obtained by combining items related to milk, cream cheese, pudding and ice cream. Food and nutrient intakes were calculated by the University of Aberdeen using the latest information in the UK food composition tables.

UK Twins

UK Twins is the biggest UK registry of volunteer twins, http://www.twinsuk.ac.uk/, started in 1992 to study the genetic and environmental aetiology associated with

age-related diseases and health aging [19]. The study comprises ~ 12,000 monozygotic and dizygotic twins across the UK with ages between sixteen and ninety eight, predominantly female twins Individuals in our study were requested free of sever diseases (e.g. cancer) and available for both measured telomere length and DNA methylation, leaving 794 female individuals (no twin pairs) remained in analysis. Leukocyte telomere length measures were based on quantitative polymerase chain reaction (qPCR, N=779), or Southern blotting method (N=346).

DNA methylation quantification

DNA samples were extracted from whole blood using DNeasy kit (Qiagen, Inc) and bisulfite converted using EZ DNA methylation kit (Zymo Research Corporation). DNA methylation levels were detected using the Illumina Infinium HumanMethylation450 array (Illumina) and the methylation betas were generated using the R package *minfi* with background correction. Raw beta levels were first applied the beta mixture quantile dilation (*BMIQ*) method to correct for the technical issues. Probe exclusion criteria including probes mapped to multiple locations to the reference sequence, and probes where more than 1% of subjects had detection p-value > 0.05. Individuals with over 5% missing probes, with mismatched sex, and with mismatched genotypes were excluded.

The Bogalusa Heart Study

The BHS started in 1972, and have recruited multiple waves of participants from childhood, adolescent and adulthood in the biracial community (65% white, 35% African American) of Bogalusa, Louisiana [20]. The longitudinal cardiovascular risk factor phenotype and genotype data of the BHS cohort are available via application through the NHLBI Biologic Specimen and Data Repository Information Coordinating Center website (https://biolincc.nhlbi.nih.gov/studies/bhs).

LTL measurements were performed by obtaining the mean length of the terminal restriction fragments, based on Southern blotting method. In brief, DNA was hybridized to a digoxigenin 3'-end labeled 5'-(CCCTAA)3 telomeric probe after overnight DNA digestion with 10 U Hinf I and 10 U Rsa I restriction enzymes as previously described [21]. Digitized autoradiograms of LTL measurement were analyzed for each sample resolved in duplicate on different gels, and the coefficient of variation for the duplicate samples was 1.4% [21].

DNA methylation

Genomic DNA was isolated from whole blood samples in the BHS using the FlexiGene DNA kit (Qiagen). The Infinium HumanMethylation450K BeadChip (Illumina, San Diego, CA) was used for whole-genome DNAm analysis. Samples were processed at the Microarray Core Facility Lab, University of Texas Southwestern Medical Center, Dallas, TX, USA. For each subject, 750ng genomic DNA was bisulfite converted using the 96 well EZ DNAm kit (Zymo Research, Irvine, CA) according to manufacturer's instructions. The efficiency of the bisulfite conversion was confirmed by in-built controls on the 450K array. The methylation profile of each participant was measured by processing 4µl of bisulfite-converted DNA, at a concentration of 50ng/µl, on a 450K array. The bisulfite converted DNA was amplified, fragmented and hybridized to the array following the protocol. We scanned the arrays by using an Illumina iScan scanner, and then the raw methylation data was extracted using Illumina's Genome Studio Methylation Module. The BHS used the data-driven separate normalization (dasen) from the wateRmelon R package [22]. The probe exclusion criteria for filtering samples and probes: 1) samples having 1% of CpG sites with a detection p-value greater than 0.05; 2) probes having 5% of samples with a detection p-value greater than 0.05; 3) probes with beadcount less than 3 in 5% of the samples.
