# Supplementary material for: DNA methylation-based estimator of telomere length
Source: Aging (Albany NY). 2019 Aug 18;11(16):5895–923. doi: 10.18632/aging.102173 (PMC6738410; doi:10.18632/aging.102173)
Supplement: Supplementary Note 2 [file aging-11-102173-s006.pdf]

SUPPLEMENTARY NOTE 2

Other datasets

UK Twins Dataset with adipose tissue

Subcutaneous adipose tissue data come from Grundberg et al. study [23]. The Illumina 450K data come from the Multiple Tissue Human Expression Resource (MuTHER) and were generated from adipose tissue data from 648 twins (Array Express ID E-MTAB1866). Additional details can be found in Grundberg et al. [23] and Horvath et al study [23]. As noted, this dataset was only served to assess the application of our DNAm TL on non-blood tissues.

Children Dataset

Blood tissue data come from our earlier study [25]. Genomic DNA was extracted and purified using the RecoverEase DNA Isolation Kit (Agilent Technologies, La Jolla, CA, USA). Bisulfite conversion using the Zymo EZ DNA Methylation Kit (ZymoResearch Organge, CA, USA) as well as sub-sequent hybridization of the Illumina 450K Bead Chip. The Illumina 450K dataset has been deposited in Gene Expression Omnibus (GSE64495).We only used the children younger than 13 years of age (N=24) in our analysis.

Liver Dataset

Liver data come from Gene Expression Omnibus (GEO) series GSE48325 and were described in Ahrens et al. [26]. Bisulphite converted DNA from these samples were hybridized to the Illumina Infinium 450K Human Methylation Beadchip.

MESA Dataset

The dataset consists of purified monocyte samples from the April 2010-February 2012 examination of 1,264 randomly selected MESA participants (55-94 years old, Caucasian (47%), African American (21%) and Hispanic (32%), female 51%) from four MESA field centers (Baltimore, MD; Forsyth County, NC; New York, NY; and St. Paul, MN). The study protocol was approved by the Institutional Review Boards at Johns Hopkins Medical Institutions, University of Minnesota, Columbia University Medical Center, and Wake Forest University Health Sciences. All participants signed informed consent. In depth details about the purification process can be found in the original publication [27]. Briefly, mononuclear cells were isolated from peripheral blood that was collected in sodium heparin tubes. Monocytes (>90% purity by FACS) were isolated using anti-CD14 monoclonal antibody coated beads. DNA methylation data was profiled using the Illumina HumanMethylation450 BeadChip and Illumina HumanHT-12 v4 Expression BeadChip platforms with standard Illumina protocols. As noted, the dataset was only used to study the correlation between chronological and DNAm LTL applied to monocytes.
