# Supplementary material for: DNA methylation-based estimator of telomere length
Source: Aging (Albany NY). 2019 Aug 18;11(16):5895–923. doi: 10.18632/aging.102173 (PMC6738410; doi:10.18632/aging.102173)
Supplement: Supplementary Methods [file aging-11-102173-s005.docx]

**SUPPLE****MENTARY METHOD**

**Estimation of blood cell counts based on DNAm levels**

We estimated blood cell counts using two different software tools. First, Houseman's estimation method [28] was used to estimate the proportions of CD8+ T cells, CD4+ T, natural killer, B cells, and granulocytes (mainly neutrophils). Second, the Horvath blood cell estimation method, implemented in the advanced analysis option of the epigenetic clock software [29, 30], was used to estimate the percentage of exhausted CD8+ T cells (defined as CD28-CD45RA-), the number (count) of naïve CD8+ T cells (defined as CD45RA+CCR7+) and plasma blasts cells. We and others have shown that the estimated blood cell counts have moderately high correlations with corresponding flow cytometric measures [28, 31].
