# Supplementary material for: DNA methylation-based estimator of telomere length
Source: Aging (Albany NY). 2019 Aug 18;11(16):5895–923. doi: 10.18632/aging.102173 (PMC6738410; doi:10.18632/aging.102173)
Supplement: Supplementary Figures [file aging-11-102173-s010.pdf]

SUPPLEMENTARY FIGURES

Supplementary Figure 1. Correlation analysis of age-adjusted DNAmTL versus age-adjusted LTL in FHS.

We present the scatter plots of DNA methylation based age-adjusted leukocyte telomere length (DNAmTLadjAge, in units of kilobase, y-axis) versus age-adjusted Southern blotting based leukocyte telomere length (LTLadjAge) (x-axis, in units of year), using Framingham Heart Study (FHS) Offspring cohort. The analysis was applied to all individuals (panel **A**) and stratified by gender (panels B&C), respectively.

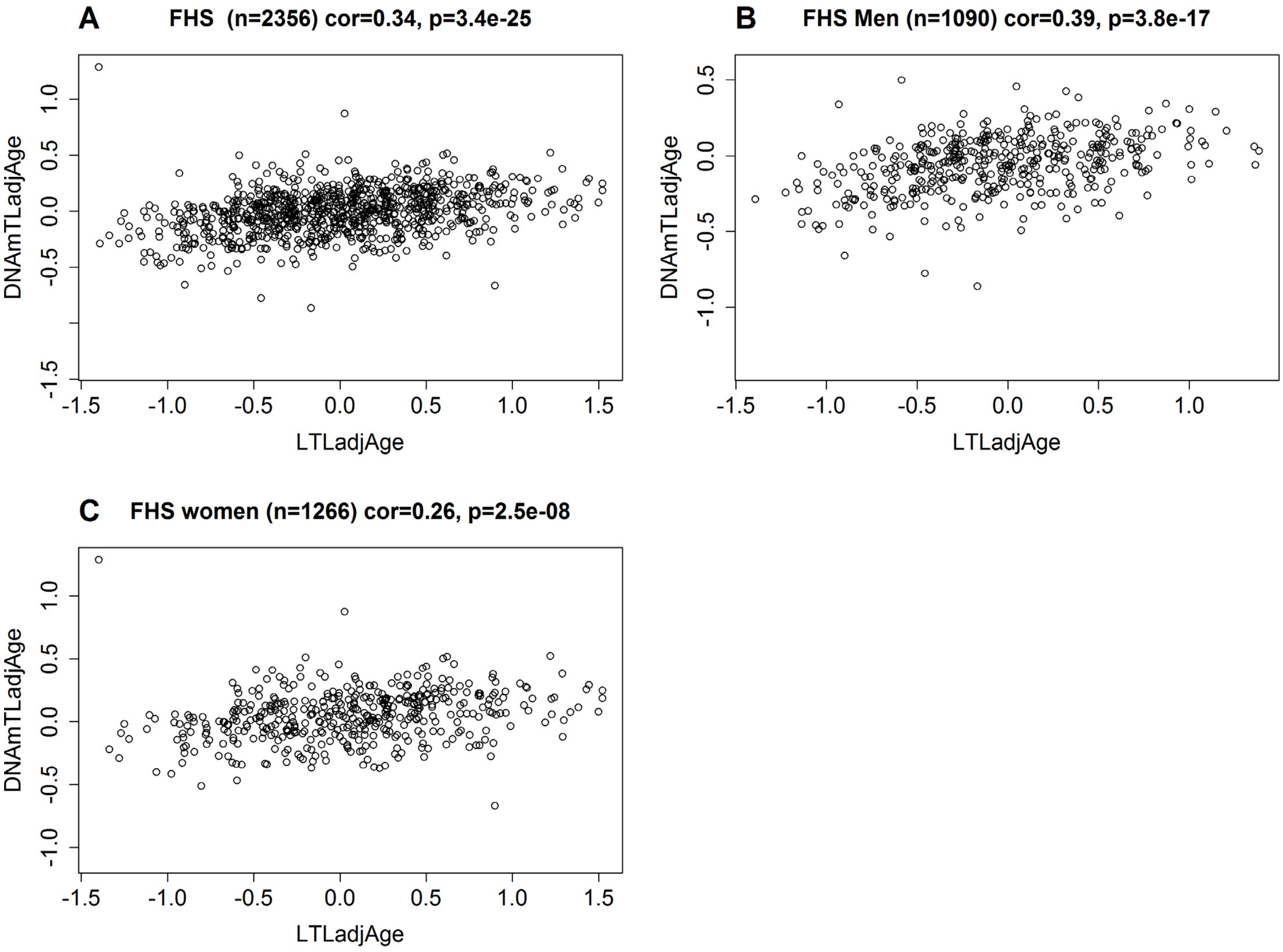

Supplementary Figure 2. Correlation analysis of age-adjusted DNAmTL versus age-adjusted LTL in BHS cohort.

We present the scatter plots of DNA methylation based leukocyte telomere length (DNAmTL, in units of kilobase, y-axis) versus Southern blotting based leukocyte telomere length (LTL) (x-axis, in units of year), using Bogalusa Herat Study (BHS) cohort. The analysis was applied to all individuals (panel **A**), stratified by gender (panels B&C), and stratified by ethic group (panels D&E), respectively.

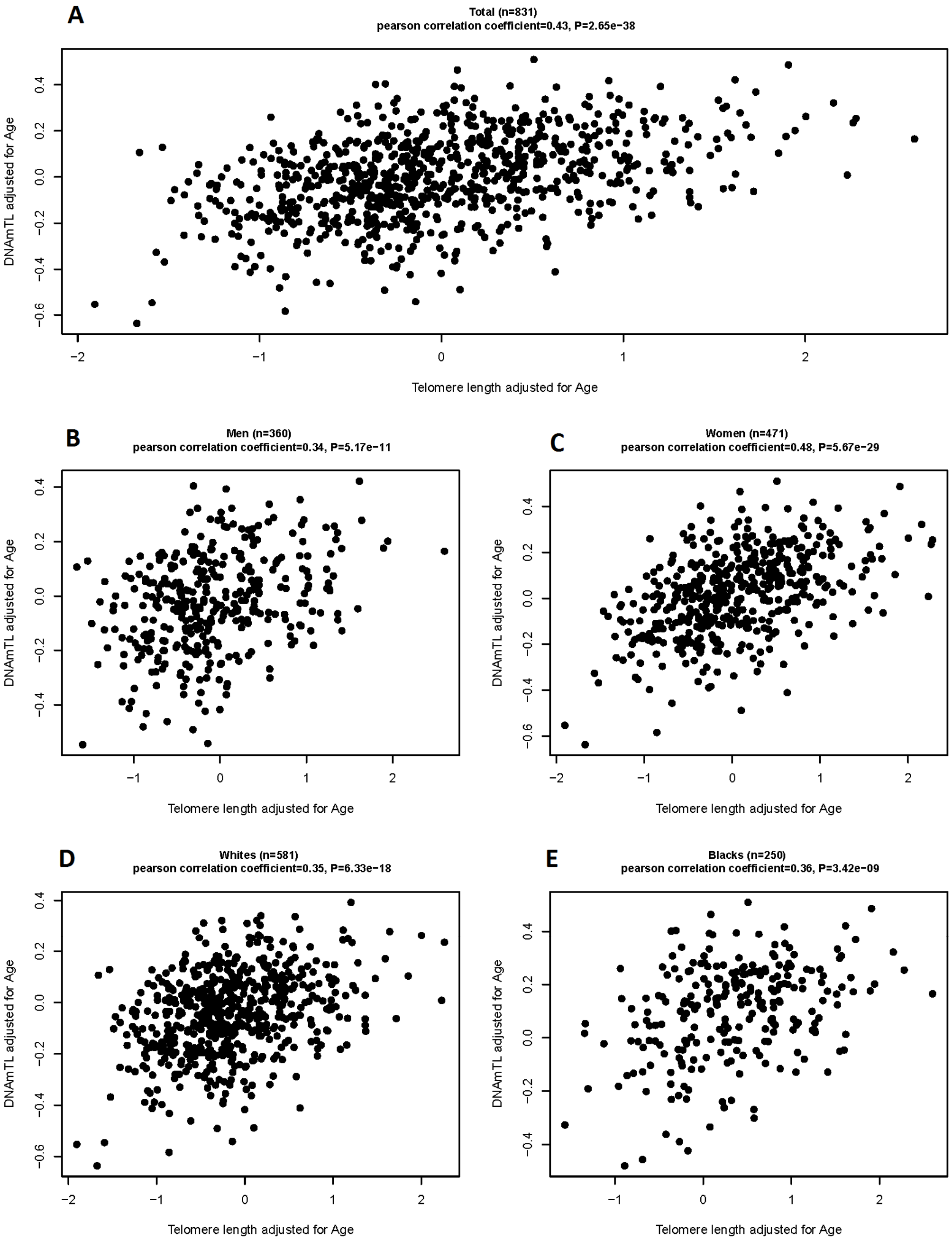

Supplementary Figure 3. Application of DNAmTL in adipose tissues.

We applied DNAmTL to publicly available adipose methylation array data from n-648 female participants of the Twins UK study {23, 24]. (**A**) Scatter plot between DNAmTL (y-axis, in units of kilobase) and chronological age reveals a significant negative Pearson correlation coefficient (r= -0.42). (**B**) Scatterplot between DNAmTL (y-axis) and TRF-based TL in n=21 individuals. (**C**) Age-adjusted DNAmTL (y-axis) versus body mass index. (**D**) Mean age-adjusted DNAmTL versus (current) smoking status (Kruskal Wallis test). DNAmTL did not differ beween former smokers and never smokers, which is why we pooled these two categories. The data are available in ArrayExpress (data identifier EMTAB1866).

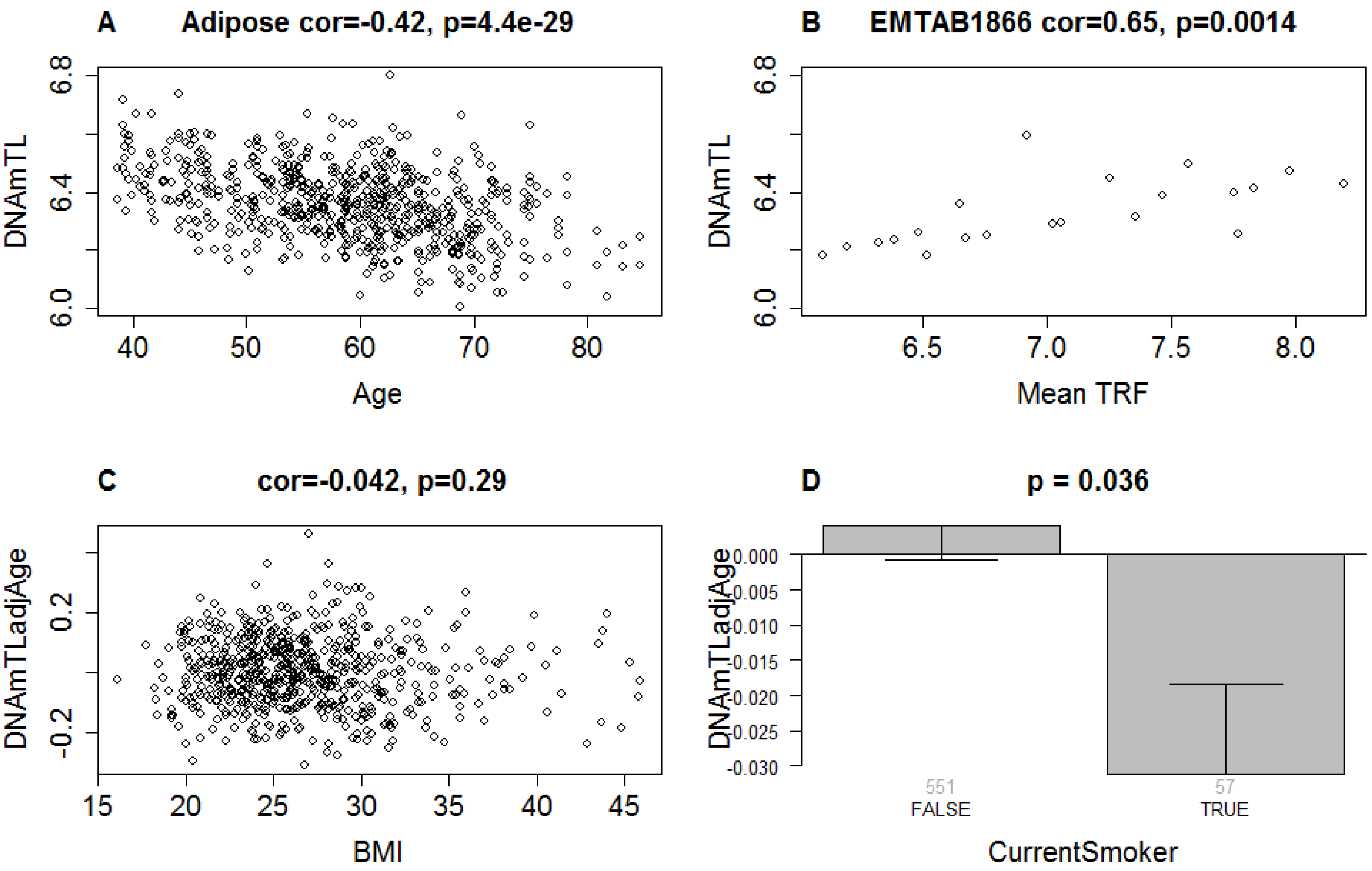

Supplementary Figure 4. DNAm biomarkers across sorted blood cell types.

We estimated DNAmTL (panel **A**), in contrast with DNAmAge [29] (panel **B**), and DNAmAgeSkinBlood [32] (panel **C**) across different blood cell types from 6 men aged from 27 and 32 years old. At each panel, we present Kruskal-Wallis P value for examining whether the distributions of the test DNAm biomarker are different across blood cell types.

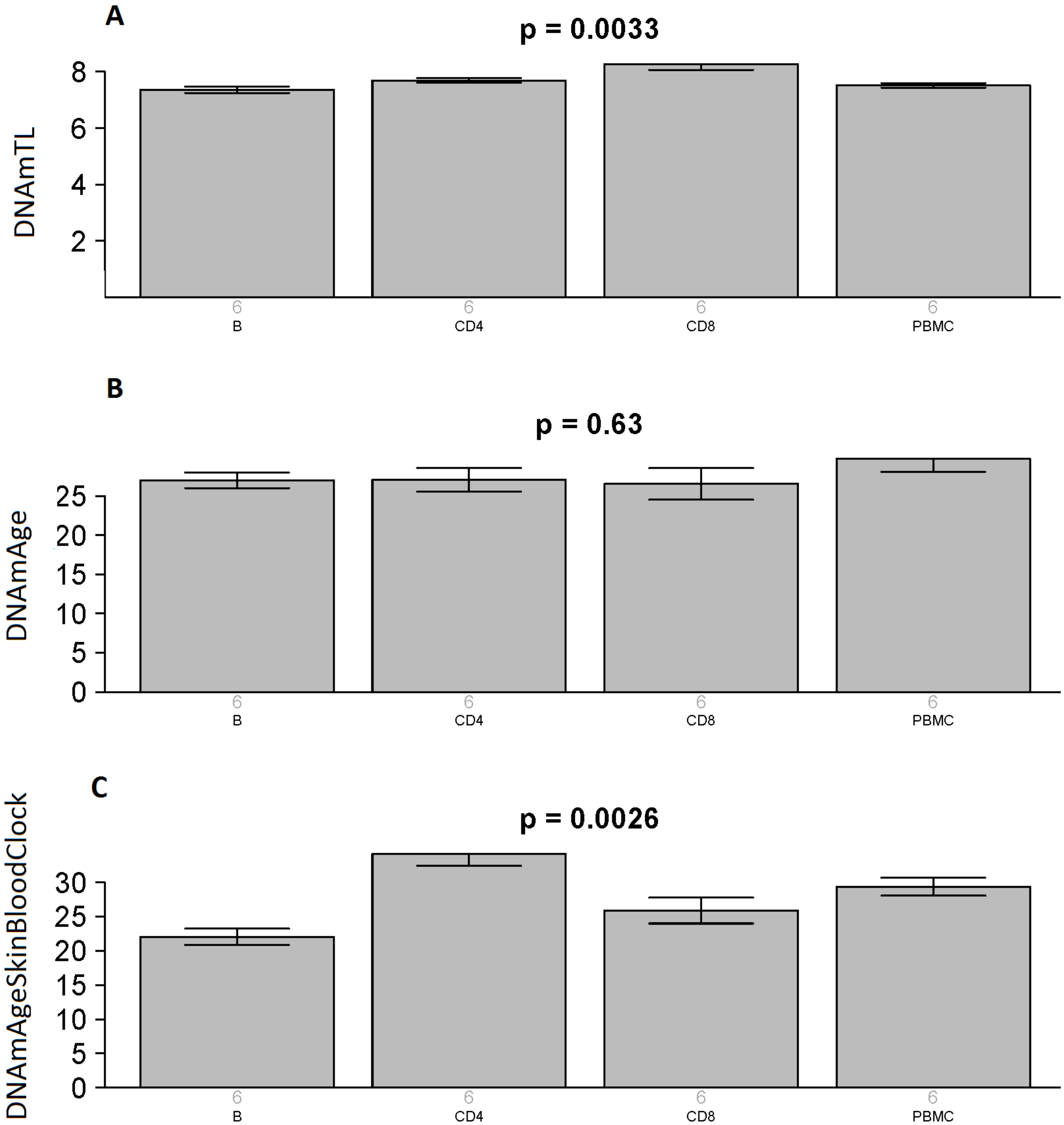

Supplementary Figure 5. DNAm TL vesus qPCR/southern plot TL in leukocytes.

We present the correlation analysis of DNAm TL (in units of kilobase [kb]) with panel (**A**) quantitative polymerase chain reaction (qPCR) based measured LTL (in units of kb) and with panel (**B**) southern plot based LTL, using 794 women from UK Twins study. Of the 794 women, 779 (98%) were available with qPCR TL and 346 (44%) were available with southern plot TL measures.

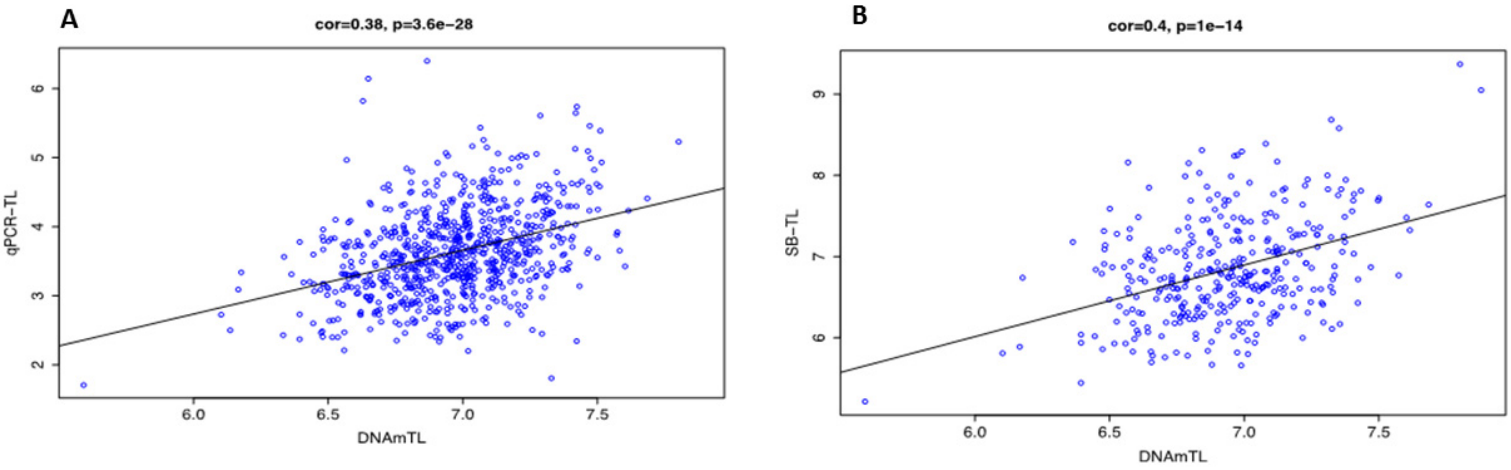

Supplementary Figure 6. Application of DNAmTL in children.

We applied DNAmTL to publicly available array data from n=24 children who were younger than 13 years [25]. (**A**) Scatter plot of DNAmTL versus chronological age. (**B**) Age-adjusted DNAmTL versus gender.

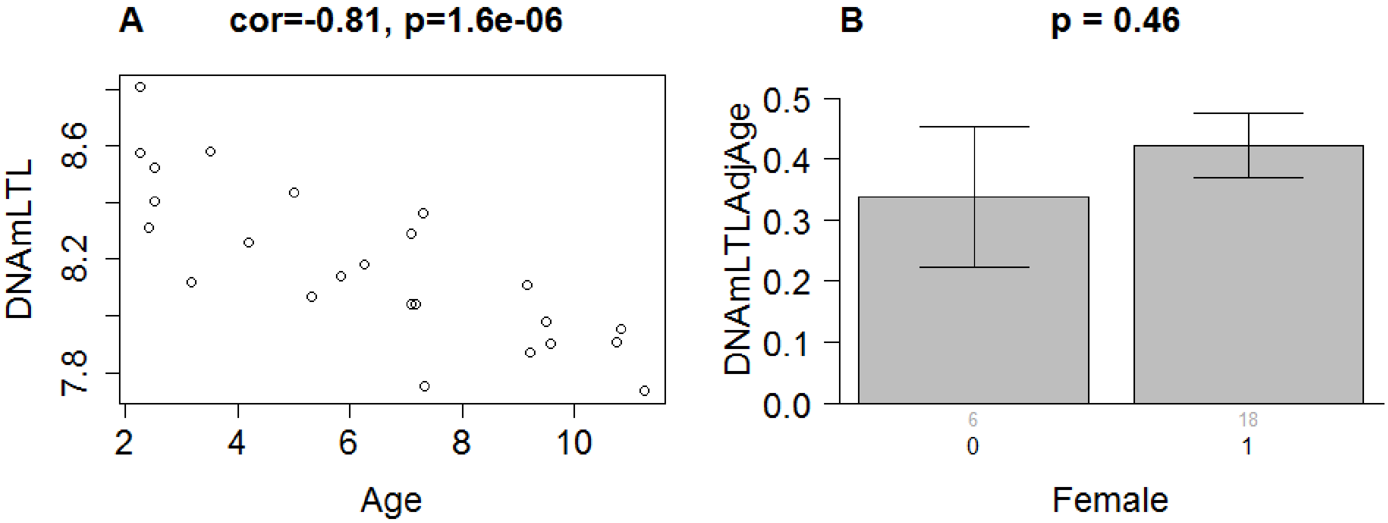

Supplementary Figure 7. Application of DNAmTL on liver tissues.

We applied DNAmTL publicly available liver methylation array data from n=85 individuals (Gene Expression Omnibus GSE48325) [24, 33] . (**A**) Scatter plot between DNAmTL (y-axis) and chronological age and Pearson correlation test. (**B**) Scatter plot between DNAmTL and body mass index. (**C**) Mean Age-adjusted DNAmTL versus gender reveals that females have higher values of DNAmTLadjAge (Kruskal Wallis test).

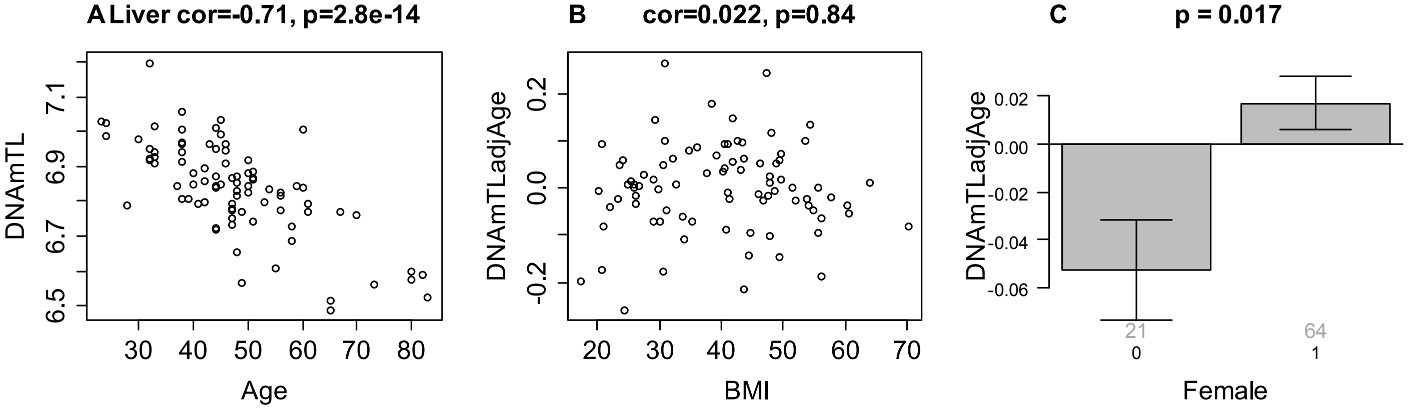

Supplementary Figure 8. Application of DNAmTL on monocytes.

DNAmTL applied to publicly available DNA methylation data from n=1,202 purified monocytes samples from the Multi-Ethnic Study of Atherosclerosis (MESA) [34]. We checked (**A**) the correlation between DNAmTL and chronological age and (**B**) the association of age-adjusted DNAmTL with ethnicity. The data can be downloaded from Gene Expression Omnibus (GSE56047).

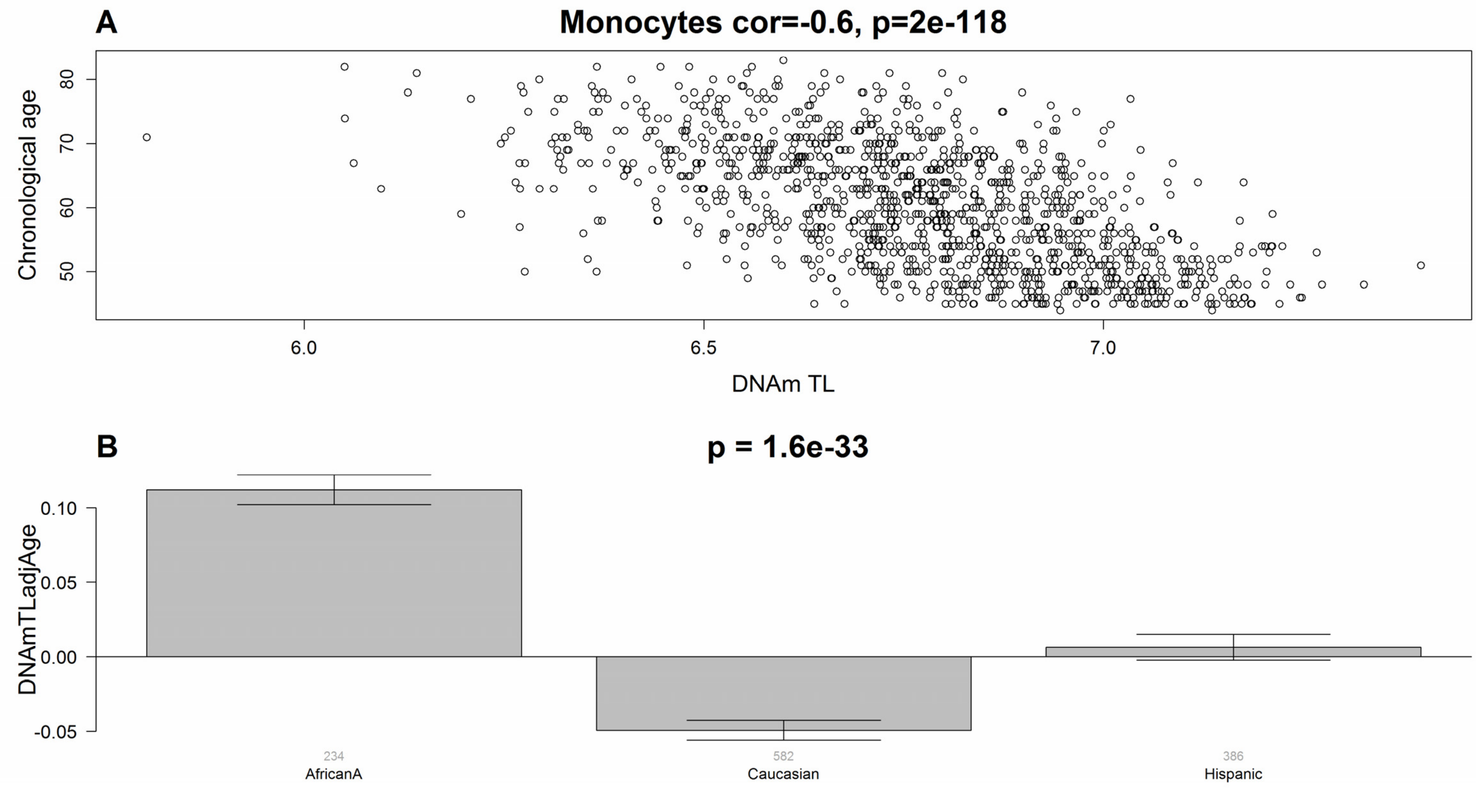

Supplementary Figure 9. Correlation analysis of chronological age versus DNAmTL.

We present the scatter plots of DNA methylation based leukocyte telomere length (DNAmTL, in units of kilobase, y-axis) versus chronological age (x-axis, in units of year) for each validation dataset. The InChianti dataset was analyzed stratified based on baseline and follow-up visit. The weak correlation estimates in the Lothian *Birth* Cohort study (panels **G** & **H**) reflect that all subjects had similar ages (within 3 years). At each validation dataset, DNA methylation was profiled in blood tissues.

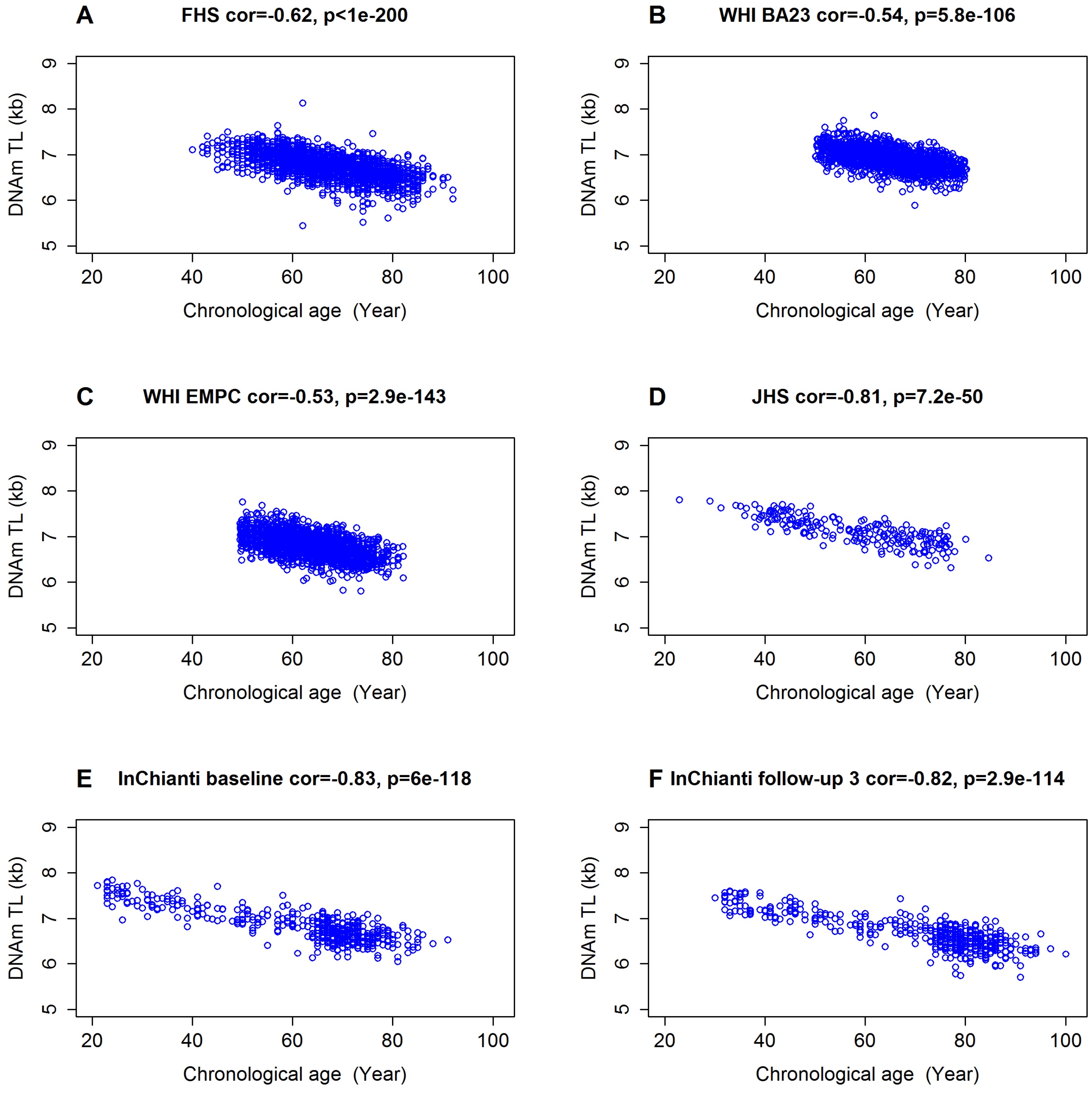

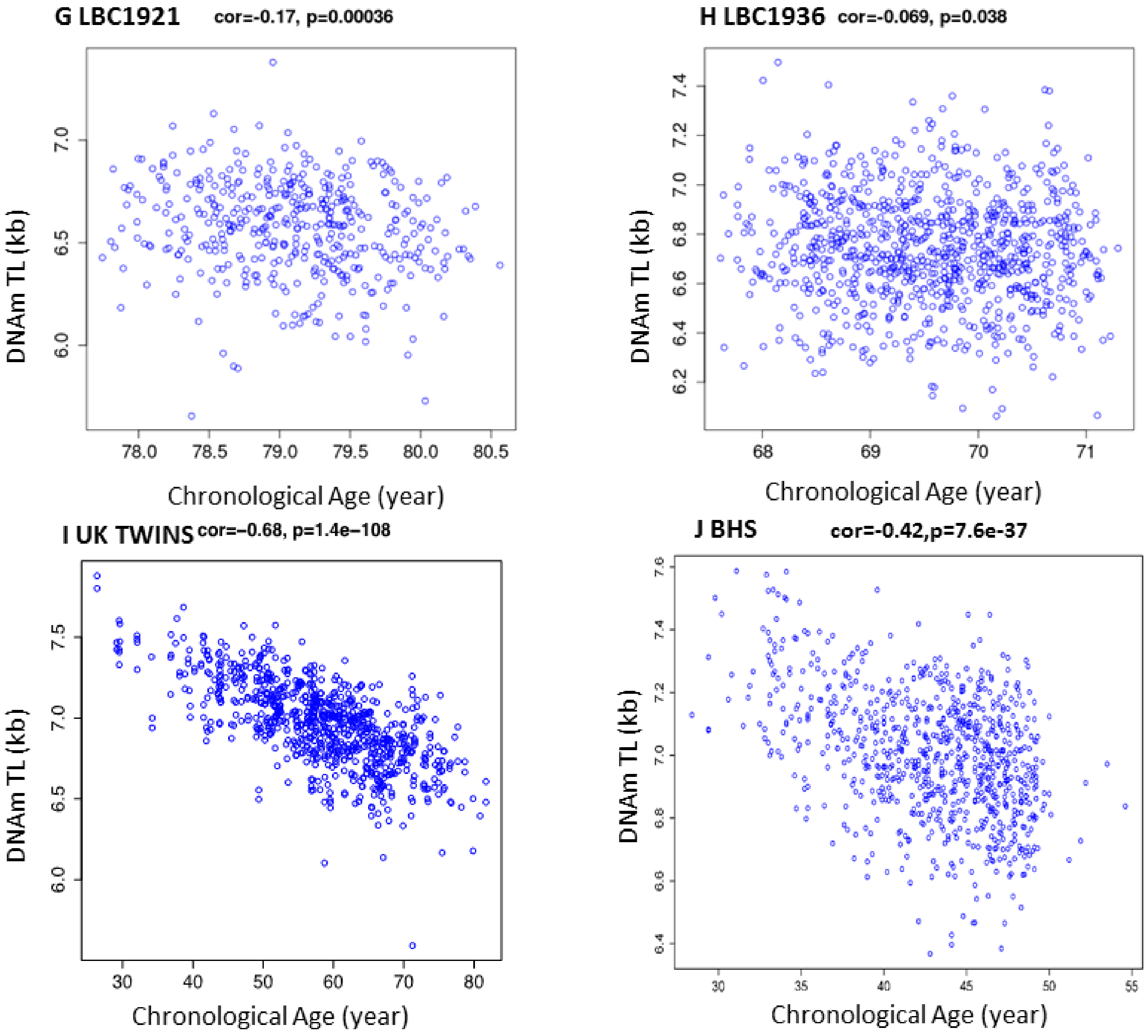
G LBC1921
cor=-0.17, p=0.00036
DNAm TL (kb)
Chronological Age (year)
H LBC1936
cor=-0.069, p=0.038
DNAm TL (kb)
Chronological Age (year)
I UK TWINS
cor=-0.68, p=1.4e-108
DNAm TL (kb)
Chronological Age (year)
J BHS
cor=-0.42,p=7.6e-37
DNAm TL (kb)
Chronological Age (year)

Supplementary Figure 10. Meta analysis forest plots for associations with age-related conditions.

We present a meta analysis forest plot for combining the effect sizes of age-adjusted DNAmTL (DNAmTLadjAge) associated with age-related conditions: (**A**) age at menopause (effect size is beta slope corresponding to the age), (**B**) the odds ratio associated with any cancer, (**C**) the hazard associated with time-to-cancer, (**D**) the P value based on Stouffer's method that combined the P values of odds ratios associated with disease free, (**E**) the P value based on Stouffer's method that combined the P values of beta slopes associated with physical function, (**F**) the P value based on Stouffer's method that combined the P values of beta slopes associated with number of age-related conditions, (**G**) the odds ratio associated with hypertension, (**H**) the odds ratio associated with dyslipidemia, and (**H**) the odds ratio associated with type 2 diabetes. All but one (panel **C**) age-related conditions were aligned with the time of blood draw.

All the analyses were adjusted for gender and adjusted for intra pedigree correlation and batch effects as needed. Each panel report a meta analysis forest plot for combining the effect sizes (if based on fixed effect meta analysis) or the P values (if based on the Stouffer's method).

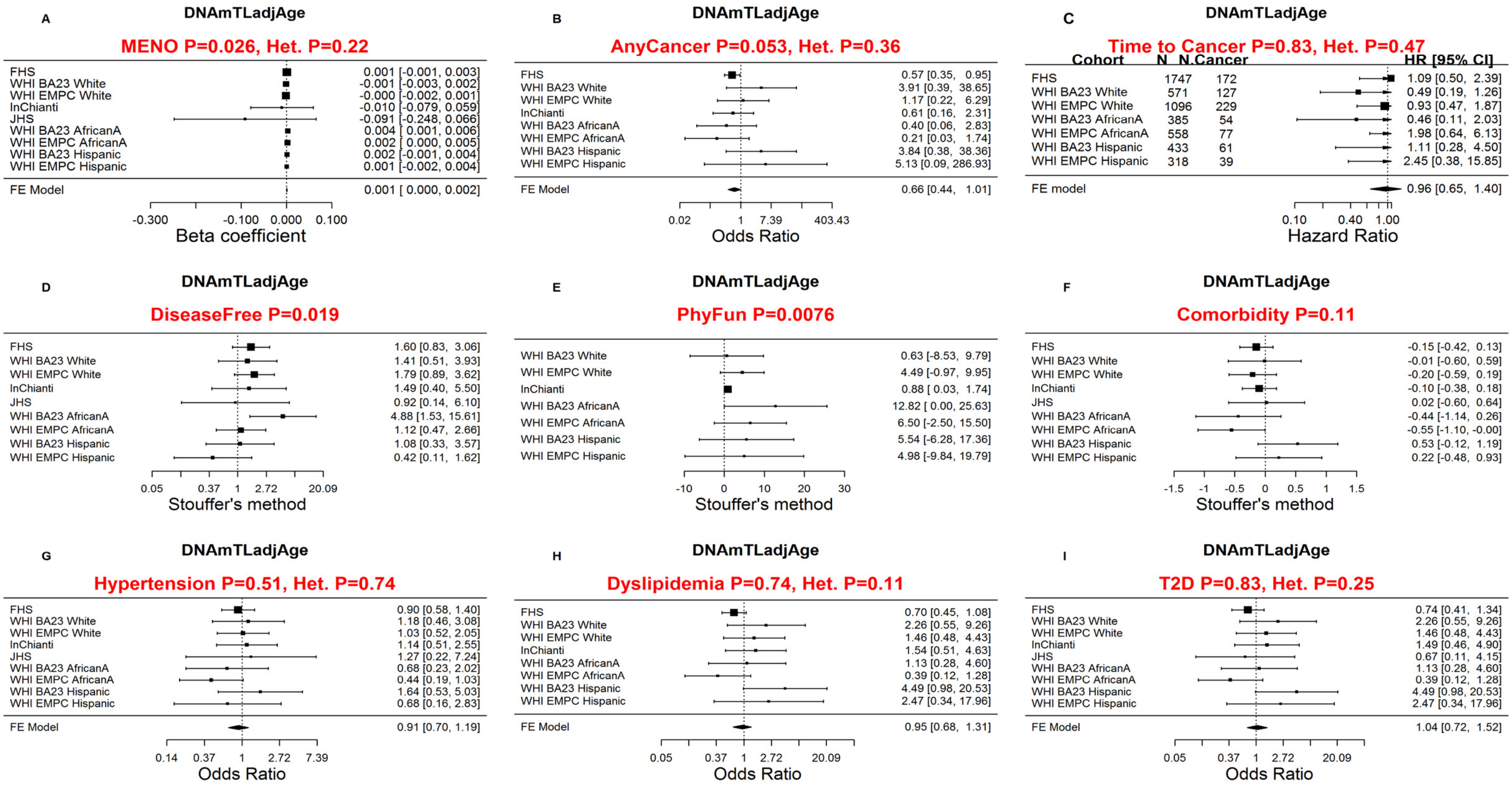

Supplementary Figure 11. Marginal correlation analysis of lifestyle factors and biomarkers versus age-adjusted DNAmTL in the FHS.

The rows of the table correspond to the associations of DNAmTLadjAge with diet, clinical biomarkers and measurements (e.g. Hemoglobin A1C), education and lifestyle factors. Using the FHS cohort, we analyzed females and males, respectively. The results were combined via fixed effect models weighted by inverse variance. We display the results from the meta analysis (All, in panel **A**), the analysis stratified by gender as listed in panel **B**. The columns report the available sample size (n) in the FHS cohort, the robust correlation coefficient (bicor, based on the biweight midcorrelation coefficient [35]), and a p-value based on a linear effects model that also adjusts for the pedigree structure (implemented in the R package "nlme"). The bicor correlation coefficients is color coded (blue to red) across its range of [-1, 1]. P-values are color-coded in green (light to dark green scale).

(A)

	Variable	N	Bicor	P	P.mixed
All	log2(1+Red meat)	2174	0.01	7.7E-01	8.4E-01
	log2(1+Poultry)	2174	-0.02	4.4E-01	7.8E-01
	log2(1+Fish)	2174	0.01	7.7E-01	8.4E-01
	log2(1+Dairy)	2157	0.06	4.3E-03	1.8E-02
	log2(1+Whole grains)	2163	0.02	4.7E-01	5.4E-01
	log2(Fruits)	2150	0.02	2.8E-01	3.6E-01
	log2(Vegetables)	2163	0.03	1.4E-01	2.3E-01
	log(OMEGA3)	2151	0.06	4.0E-03	1.3E-02
	A1C	2355	0.00	9.8E-01	1.2E-01
	log2(C-reactive protein)	2347	-0.11	3.1E-07	3.1E-07
	log2(Glucose)	2354	-0.04	7.3E-02	1.7E-02
	log2(Triglyceride)	2354	-0.06	5.3E-03	1.4E-02
	Total cholesterol	2354	0.04	3.4E-02	1.6E-02
	HDL cholesterol	2353	0.03	2.0E-01	3.5E-01
	log2(Creatinine)	2347	0.02	2.4E-01	9.1E-01
	log2(Urine Creatinine)	2319	-0.03	1.6E-01	1.9E-01
	Systolic blood pressure	2356	-0.02	4.6E-01	5.4E-01
	Diastolic blood pressure	2355	0.06	4.9E-03	7.2E-03
	log2(Waist / hip ratio)	2324	-0.04	3.3E-02	6.6E-02
	BMI	2348	-0.03	1.6E-01	3.7E-01
	Education	2029	0.04	5.1E-02	2.2E-02
	Current smoker	2356	-0.12	6.7E-09	6.9E-07
	log2(1+Alcohol)	2353	-0.03	2.1E-01	3.4E-01
	log(Vitamin E)	2142	-0.02	4.4E-01	1.6E-01

(B) Stratified by gender

	Variable	N	Bicor	P	P.mixed
Female	log2(1+Red meat)	1175	0.02	4.0E-01	5.7E-01
	log2(1+Poultry)	1175	0.00	9.1E-01	8.9E-01
	log2(1+Fish)	1175	0.02	4.0E-01	5.7E-01
	log2(1+Dairy)	1165	0.09	3.1E-03	2.0E-02
	log2(1+Whole grains)	1169	0.01	7.7E-01	7.4E-01
	log2(Fruits)	1165	0.02	4.7E-01	7.9E-01
	log2(Vegetables)	1170	0.01	7.2E-01	9.6E-01
	log(OMEGA3)	1159	0.05	1.1E-01	2.8E-01
	A1C	1265	0.03	3.7E-01	9.7E-01
	log2(C-reactive protein)	1259	-0.09	1.6E-03	3.8E-03
	log2(Glucose)	1265	-0.01	6.5E-01	1.6E-01
	log2(Triglyceride)	1265	-0.07	1.6E-02	3.1E-02
	Total cholesterol	1265	0.07	1.9E-02	2.5E-02
	HDL cholesterol	1264	0.02	4.7E-01	7.8E-01
	log2(Creatinine)	1259	0.01	8.4E-01	3.9E-01
	log2(Urine Creatinine)	1239	-0.01	7.7E-01	8.7E-01
	Systolic blood pressure	1266	-0.03	2.3E-01	1.5E-01
	Diastolic blood pressure	1265	0.05	8.5E-02	7.0E-02
	log2(Waist / hip ratio)	1248	-0.05	6.6E-02	1.0E-01
	BMI	1264	-0.04	1.8E-01	4.5E-01
	Education	1094	0.07	1.7E-02	3.8E-03
	Current smoker	1266	-0.12	3.3E-05	1.3E-03
	log2(1+Alcohol)	1264	-0.02	5.4E-01	7.7E-01
	log(VitaminE)	1161	-0.02	5.3E-01	1.5E-01
Male	log2(1+Red meat)	999	-0.02	6.3E-01	7.2E-01
	log2(1+Poultry)	999	-0.03	3.1E-01	5.4E-01
	log2(1+Fish)	999	-0.02	6.3E-01	7.2E-01
	log2(1+Dairy)	992	0.03	3.2E-01	3.4E-01
	log2(1+Whole grains)	994	0.02	4.4E-01	5.7E-01
	log2(Fruits)	985	0.03	4.2E-01	2.9E-01
	log2(Vegetables)	993	0.06	7.6E-02	7.0E-02
	log(OMEGA3)	992	0.08	1.2E-02	1.4E-02
	A1C	1090	-0.03	3.2E-01	5.9E-03
	log2(C-reactive protein)	1088	-0.12	4.1E-05	1.7E-05
	log2(Glucose)	1089	-0.07	3.2E-02	3.8E-02
	log2(Triglyceride)	1089	-0.05	1.4E-01	2.1E-01
	Total cholesterol	1089	0.02	5.5E-01	2.6E-01
	HDL cholesterol	1089	0.03	2.8E-01	3.2E-01
	log2(Creatinine)	1088	0.05	1.3E-01	4.4E-01
	log2(Urine Creatinine)	1080	-0.05	8.1E-02	5.5E-02
	Systolic blood pressure	1090	0.01	8.4E-01	5.2E-01
	Diastolic blood pressure	1090	0.07	2.3E-02	4.5E-02
	log2(Waist / hip ratio)	1076	-0.04	2.5E-01	2.9E-01
	BMI	1084	-0.02	5.3E-01	6.0E-01
	Education	935	0.01	7.8E-01	9.5E-01
	Current smoker	1090	-0.12	5.3E-05	1.7E-04
	log2(1+Alcohol)	1089	-0.04	2.4E-01	2.0E-01
	log(VitaminE)	981	-0.01	6.5E-01	6.1E-01

Supplementary Figure 12. Marginal correlation analysis of lifestyle factors and biomarkers versus age-adjusted DNAmTL in the WHI stratified by ethnicity.

The rows of the table correspond to the associations of DNAmTLadjAge with diet, clinical biomarkers and measurements (e.g. C-reactive protein), education and lifestyle factors. Using the WHI cohort, we perform bicor correlation analysis for each ethnic group, respectively. The results were combined via fixed effect models weighted by inverse variance. We display the results from the meta analysis (All), and the analysis stratified by ethnic groups: White (N up to 1635), African American (N up to 924), and Hispanic (N up to 719). The columns report the available sample size (N) in the WHI cohort, the robust correlation coefficient and P value (bicor, based on the biweight midcorrelation coefficient [35]. The bicor correlation coefficients is color coded (blue to red) across its range of [-1, 1]. P-values are color-coded in green (light to dark green scale).

		ALL			White			AfricanA			Hispanic		
**Category**	**Variable**	**N**	**bicor**	**P**	**N**	**bicor**	**P**	**N**	**bicor**	**P**	**N**	**bicor**	**P**
**Diet**	log2(Total energy)	2789	0.00	9.5E-01	1377	0.01	6.3E-01	790	-0.05	1.6E-01	622	0.04	3.3E-01
	Carbohydrate	2789	0.04	2.1E-02	1377	0.03	3.2E-01	790	0.05	1.8E-01	622	0.08	6.1E-02
	Protein	2789	0.01	6.9E-01	1377	0.02	4.3E-01	790	0.00	9.3E-01	622	-0.01	8.3E-01
	Fat	2789	-0.04	3.7E-02	1377	-0.03	3.0E-01	790	-0.01	7.6E-01	622	-0.10	1.1E-02
	log2(1+Red meat)	2789	-0.03	6.8E-02	1377	-0.03	2.5E-01	790	-0.05	1.4E-01	622	-0.02	6.1E-01
	log2(1+Poultry)	2789	-0.02	3.5E-01	1377	-0.02	4.0E-01	790	-0.04	2.1E-01	622	0.03	4.9E-01
	log2(1+Fish)	2789	0.05	4.7E-03	1377	0.05	9.3E-02	790	0.04	2.6E-01	622	0.09	2.8E-02
	log2(1+Dairy)	2789	0.01	5.0E-01	1377	0.03	2.8E-01	790	-0.03	4.5E-01	622	0.03	4.9E-01
	log2(1+Whole grains)	2789	0.01	4.5E-01	1377	0.00	8.7E-01	790	0.06	1.0E-01	622	0.00	9.9E-01
	log2(1+Nuts)	2789	0.03	1.0E-01	1377	0.06	2.0E-02	790	-0.01	7.9E-01	622	0.01	7.9E-01
	log2(Fruits)	2789	0.07	3.3E-04	1377	0.10	1.8E-04	790	0.00	9.4E-01	622	0.08	5.5E-02
	log2(Vegetables)	2789	0.03	9.7E-02	1377	0.02	4.8E-01	790	0.03	3.7E-01	622	0.06	1.5E-01
**Dietary Biomarkers**	Retinol	2020	-0.02	4.2E-01	1099	-0.04	1.6E-01	575	-0.01	8.9E-01	346	0.04	4.8E-01
	Mean carotenoids	2019	0.10	1.5E-05	1099	0.11	1.4E-04	574	0.11	8.5E-03	346	0.01	8.1E-01
	Lycopene	2020	0.04	9.5E-02	1099	0.05	1.1E-01	575	0.05	2.1E-01	346	-0.02	6.9E-01
	log2(alpha-Carotene)	2020	0.08	3.9E-04	1099	0.08	7.0E-03	575	0.11	6.5E-03	346	0.01	8.1E-01
	log2(beta-Carotene)	2019	0.07	9.5E-04	1099	0.08	9.7E-03	574	0.09	2.6E-02	346	0.03	6.2E-01
	log2(Lutein+Zeaxanthin)	2020	0.06	3.5E-03	1099	0.08	6.5E-03	575	0.05	2.1E-01	346	0.03	5.6E-01
	log2(beta-Cryptoxanthin)	2020	0.10	3.8E-06	1099	0.14	6.8E-06	575	0.09	3.2E-02	346	0.02	7.2E-01
	log2(alpha-Tocopherol)	2020	0.05	3.2E-02	1099	0.07	1.3E-02	575	0.00	9.7E-01	346	0.04	4.3E-01
	log2(gamma-Tocopherol)	2020	-0.06	1.1E-02	1099	-0.08	9.3E-03	575	-0.04	4.0E-01	346	-0.02	6.6E-01
**Measurements**	log2(C-reactive protein)	2036	-0.06	4.4E-03	898	-0.10	3.7E-03	622	-0.02	6.3E-01	516	-0.06	2.0E-01
	log2(Insulin)	3175	-0.06	3.5E-04	1579	-0.05	3.2E-02	896	-0.05	1.6E-01	700	-0.11	5.2E-03
	log2(Glucose)	3259	-0.01	5.3E-01	1631	0.02	4.4E-01	913	-0.02	4.8E-01	715	-0.06	9.0E-02
	log2(Triglyceride)	3263	-0.04	2.4E-02	1635	-0.03	3.1E-01	913	-0.07	2.7E-02	715	-0.03	4.5E-01
	Total cholesterol	3263	0.00	7.8E-01	1635	0.00	9.1E-01	913	-0.01	7.9E-01	715	0.04	2.9E-01
	LDL cholesterol	3215	0.00	8.6E-01	1606	0.00	9.2E-01	908	-0.01	8.2E-01	701	0.03	4.3E-01
	HDL cholesterol	3260	0.06	1.6E-03	1632	0.04	1.5E-01	913	0.08	1.6E-02	715	0.07	6.6E-02
	log2(Creatinine)	1985	-0.03	2.1E-01	871	-0.05	1.5E-01	612	-0.06	1.4E-01	502	0.05	3.1E-01
	Systolic blood pressure	3283	0.00	8.6E-01	1640	0.01	6.7E-01	924	-0.04	2.5E-01	719	0.01	7.7E-01
	Diastolic blood pressure	3284	0.01	4.2E-01	1641	0.03	1.8E-01	924	-0.02	6.5E-01	719	0.01	8.4E-01
	log2(Waist / hip ratio)	3142	-0.04	3.0E-02	1552	-0.02	3.5E-01	887	-0.05	1.6E-01	703	-0.06	1.0E-01
	BMI	3257	-0.03	1.0E-01	1630	-0.03	2.4E-01	915	-0.03	4.2E-01	712	-0.03	4.3E-01
**Life style**	Education	3255	0.08	1.6E-05	1632	0.10	1.1E-04	915	0.04	2.7E-01	708	0.08	3.4E-02
	Income	3186	0.07	3.1E-05	1597	0.10	6.5E-05	896	0.00	9.6E-01	693	0.11	3.7E-03
	log2(1+Exercise)	3061	0.04	2.4E-02	1479	0.04	1.8E-01	893	0.03	3.7E-01	689	0.07	7.9E-02
	Current smoker	3235	-0.09	3.7E-07	1620	-0.10	1.0E-04	905	-0.14	2.6E-05	710	-0.01	8.4E-01
	log2(1+Alcohol)	2789	0.02	3.0E-01	1377	0.02	4.7E-01	790	-0.01	8.0E-01	622	0.06	1.6E-01

Supplementary Figure 13. Age-adjusted DNAm TL significantly associated with smoking status.

We present association analysis of age-adjusted DNAm TL with smoking status (current versus never/former smokers), using Bogalusa Heart Study (BHS, N=831) cohort. Our analysis showed that current smokers were associated with significant shorter age-adjusted DNAm TL in the whole samples (panel **A**) and in each subgroup stratified by gender (panels **B** & **C**) or ethnicity (panels **D** & **E**).

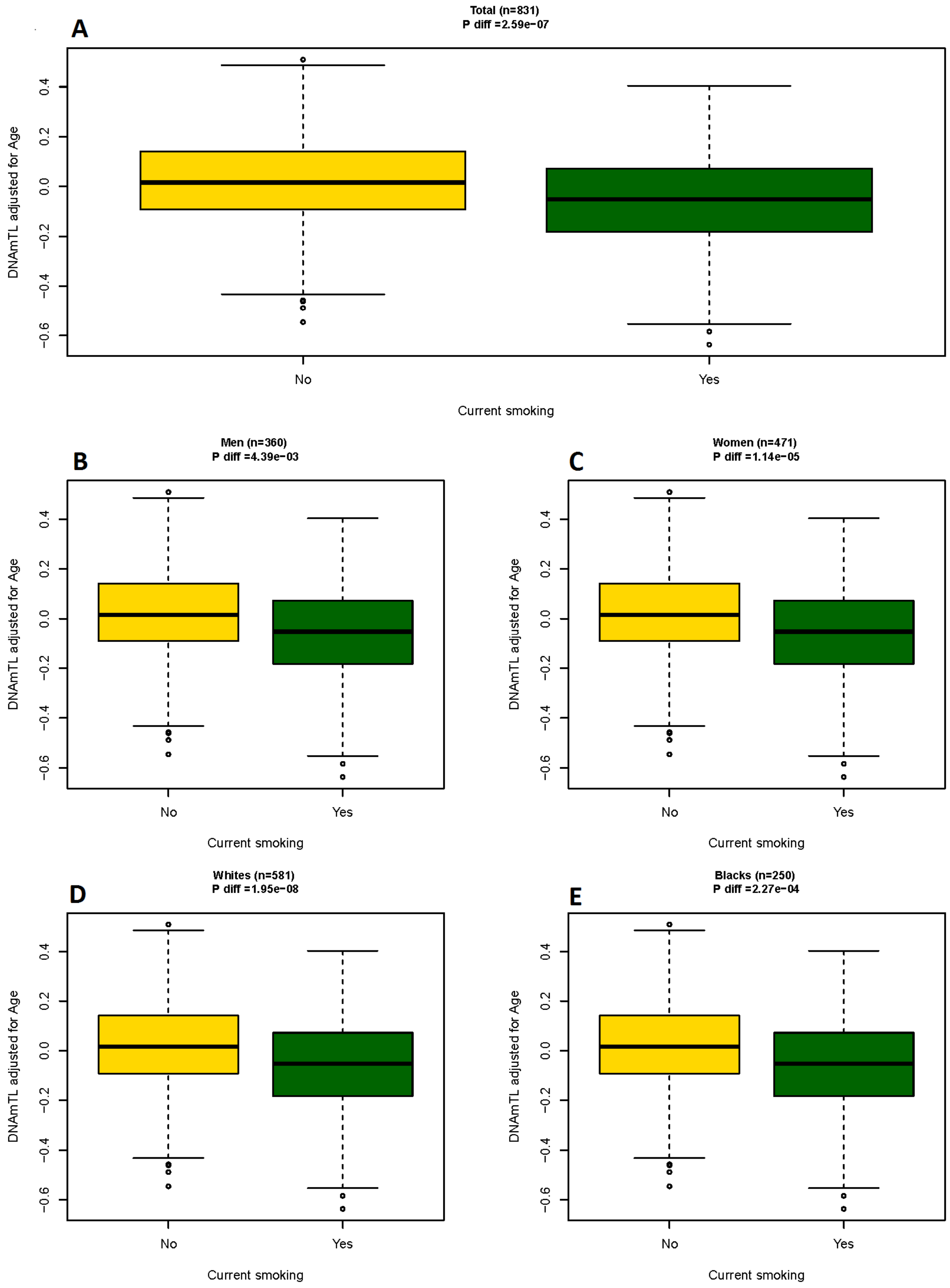

Supplementary Figure 14. Age-adjusted LTL not affected smoking status.

We present association analysis of age-adjusted LTL with smoking status (current versus never/former smokers), using Bogalusa Heart Study (BHS, N=831) cohort. Our analysis showed that current smokers were not associated with significant shorter age-adjusted DNAm TL in the whole samples (panel **A)** and in each subgroup stratified by gender (panels **B** & **C**) or ethnicity (panels **D** & **E**).

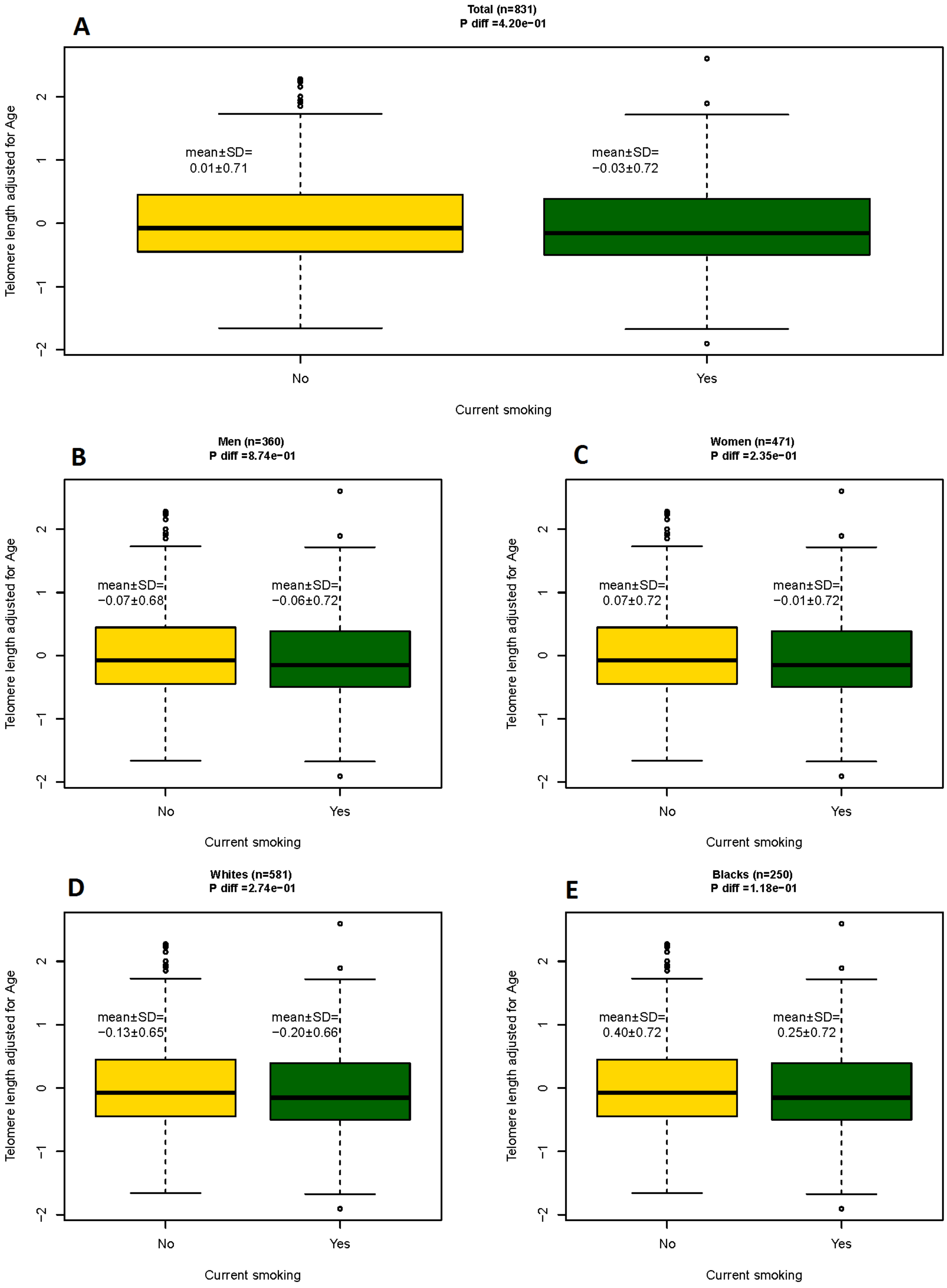

Supplementary Figure 15. Impact of OMEGA3 on age-adjusted leukocyte telomere length measures.

We investigated the impact of OMEGA-3 FATTY-ACIDS supplement on age-adjusted DNAmTL (DNAmTLadjAge) and age-adjusted LTL (LTLadjAge), respectively. The variable of OMEGA-3 intake was log transformed, log (OMEGA-3+1), to improve its normality. Panel **A** presents the robust correlation analysis using the entire FHS cohort (N=2147) and Panel **B** presents the robust correlation analysis based on the 878 individuals whom were available with LTL measures based on terminal restriction fragmentation (TRF) method.

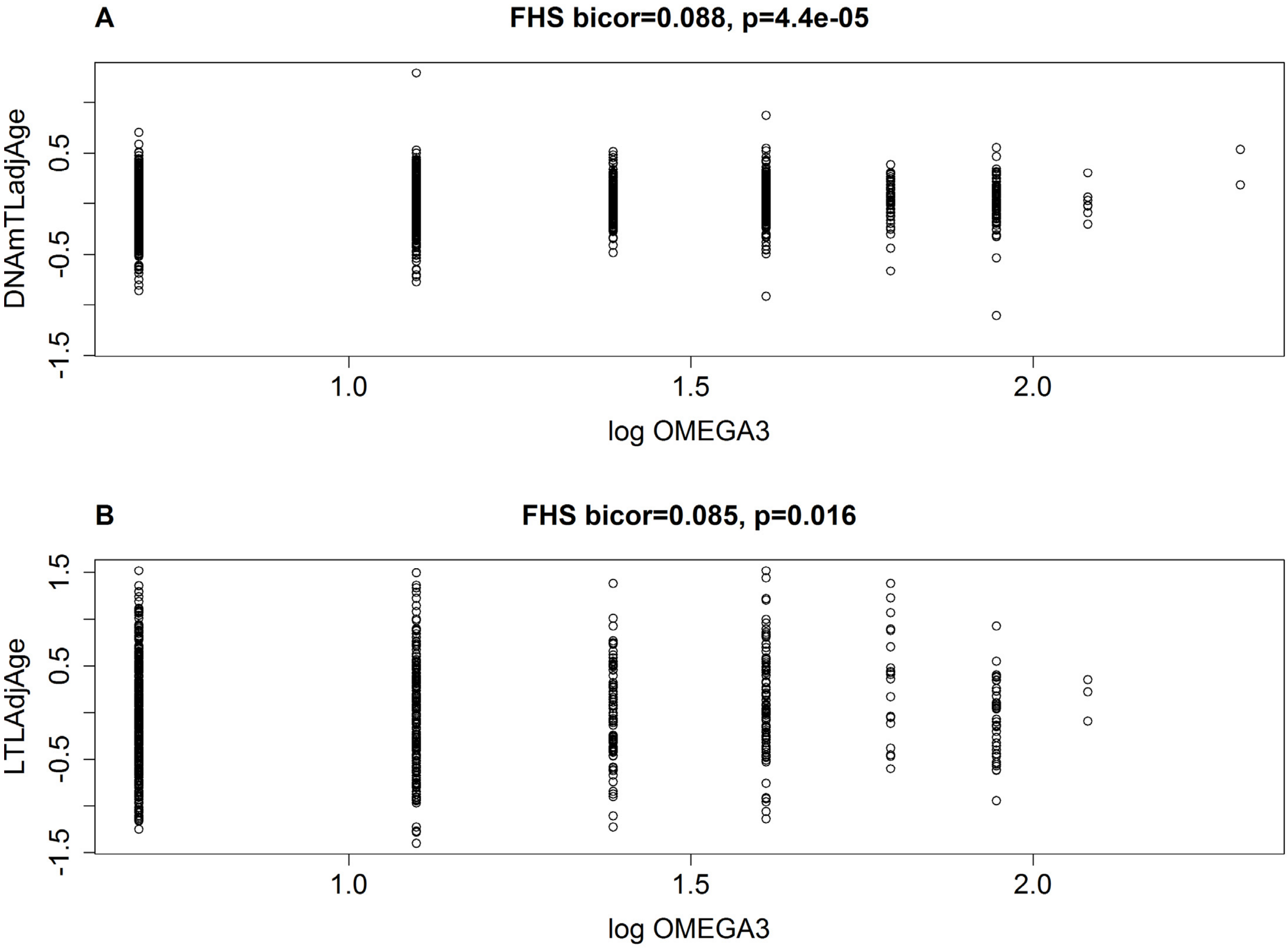

Supplementary Figure 16. Measures of blood cell composition versus leucocyte telomere length measures.

We present the bar plots to depict the associations of age-adjusted leucocyte telomere length (LTL) and its surrogate age-adjusted DNAmTL with 10 imputed measures of blood cell counts, respectively. The height of each bar corresponding to the statistical significance level (meta analysis p-value) of an association test between the blood cell measure and the age-adjusted biomarker. More precisely, the y-axis presents minus logarithm (base 10) transformed meta P values. The numbers displayed on top of each bar are the meta analysis estimates of the correlation coefficients. The association analysis is *not* confounded by chronological age because we used age adjusted biomarkers. Panels **A** and **B** present the fixed effects meta analysis (N=3,134) performed across two training dataset: WHI BA23 (N=718) and JHS (N=1538), and one test dataset: FHS (N=878). Sensitivity analysis was performed on the test dataset only (FHS) to demonstrate the results not biased by the training process.Abbreviations for cell counts are listed in the following: nature killer (NK), monocyte (MONO) and granulocyte (Gran), CD8pCD28nCD45Ran (CD8.ex for exhausted cytotoxic T cells), and plasma blast (PB). The blood cell counts were imputed based on DNA methylation levels as described in [28, 36] and the **Supplementary Methods section** (above).

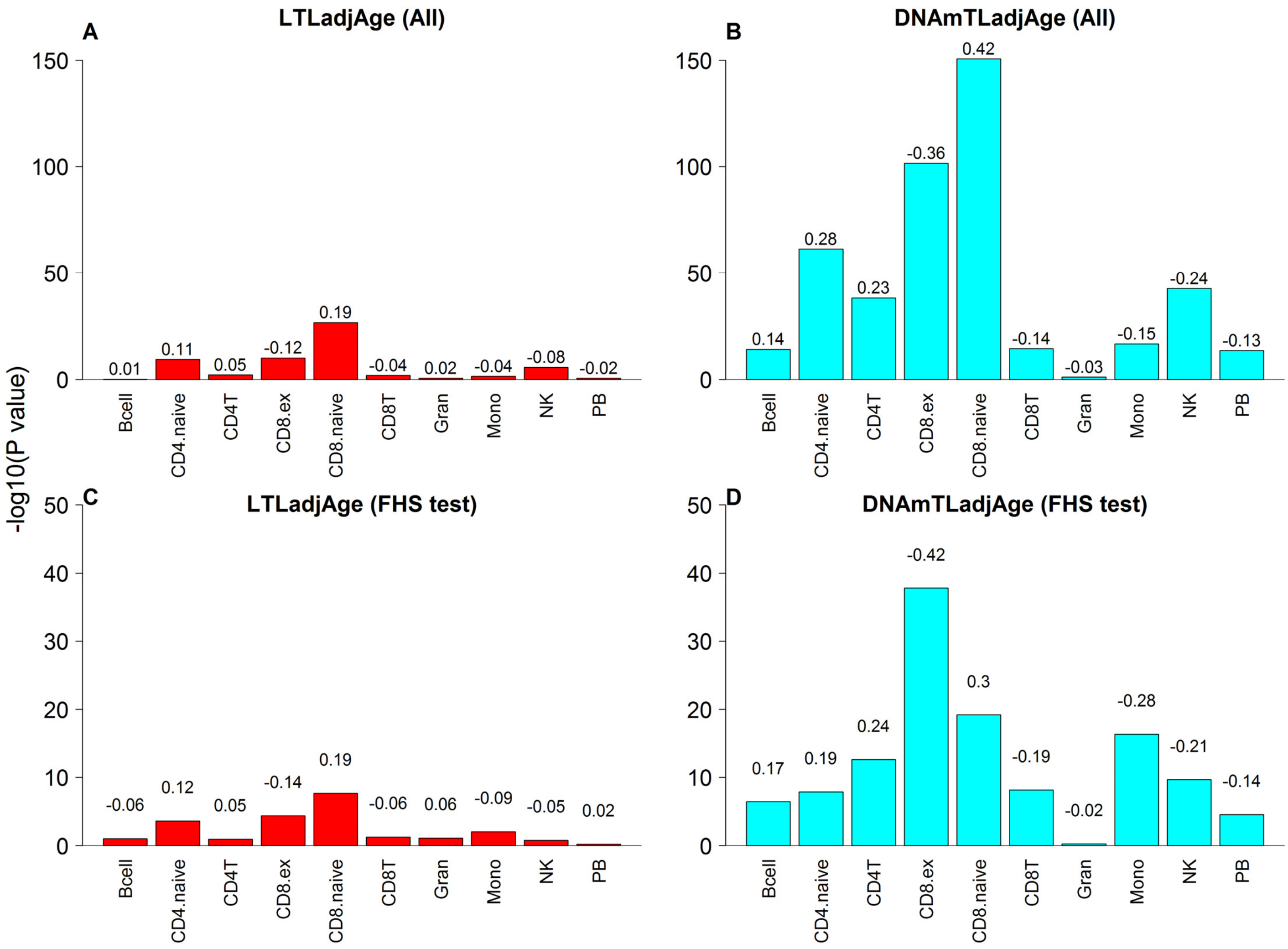

Supplementary Figure 17. Heat map of pairwise correlations of DNAm based biomarkers.

The heat map color-codes the pairwise Pearson correlations of DNAmTLadjAge and four epigenetic age acceleration measures: AgeAccelGrim [37], age-adjusted DNAm PhenoAge (AgeAccelPheno) [38], age-adjusted DNAm age based on Hannum et al. (AgeAccelHannum) [39], and age adjusted DNAm age based on Horvath (AgeAccel) [29], performed using the Framingham Heart Study (N=2356). The figure also includes an estimator of mortality risk, mortality.res, which can be interpreted as a measure of "excess" mortality risk compared to the baseline risk in the test data. Formally, mortality.res is defined as the deviance residual from a Cox regression model for time-to-death due to all-cause mortality. The shades of color (blue, white, and red) visualize correlation values from -1 to 1. Each square reports the Pearson correlation coefficient.

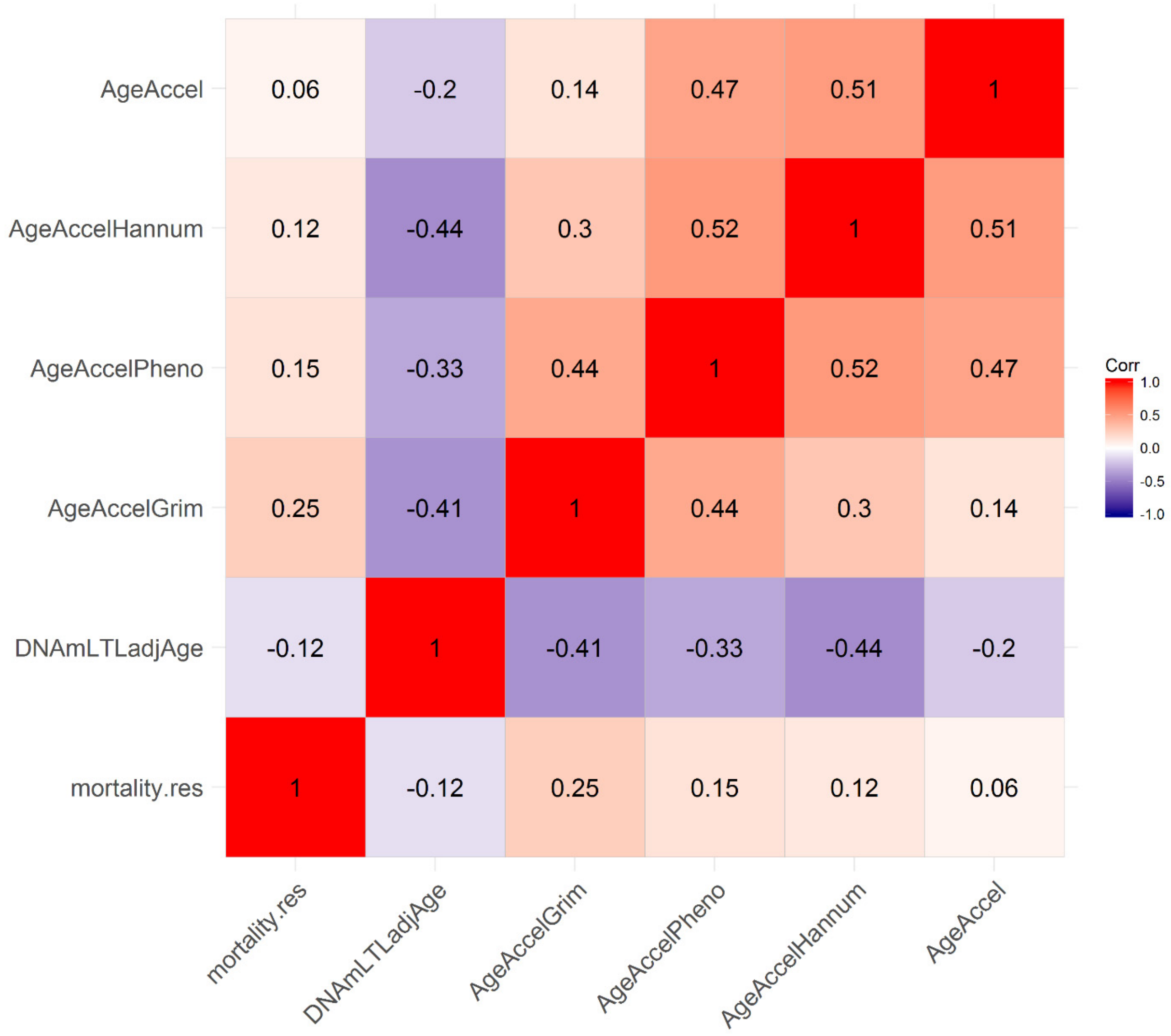

Supplementary Figure 18. Comparing DNAmTLadjAge and epigenetic measures of age acceleration in predicting life and health span.

We compare age-adjusted DNAmTL (DNAmTLadjAge) with four well-known epigenetic measures of age acceleration: 1) age-adjusted DNAm GrimAge based on Lu et al. [37], (AgeAccelGrim), 2) age-adjusted DNAm PhenoAge (AgeAccelPheno) [38], 3) age-adjusted DNAm age based on Hannum et al. (AgeAccelHannum) [39], and 4) age adjusted DNAm age based on Horvath (AgeAccelerationResidual) [29], for their associations with life and health span. Three survival outcomes, time-to-death (panels A-E), time-to-coronary heart disease (CHD, panels F-J), and time-to congestive heart failure (CHF, panels K-O), were evaluated across each available validation dataset, stratified by race/ethnicity. Cox regression analysis was performed to predict the hazards of DNAmTLadjAge and each epigenetic measures of age acceleration variables, adjusted for age, gender and adjusted for intra pedigree correlation and batch effects as needed. Each row presents the summary statistic at a (stratified) study dataset and reports sample size (N), number of events, P value, hazard ratio and a 95% confidence interval resulting from a Cox regression model. Panels (**A**-**O**) are based on all the available validation datasets. As parts of the FHS cohort were used to train the DNAm GrimAge, we performed sensitive analysis that removed the entire FHS cohort and observed similar patterns (Panels).

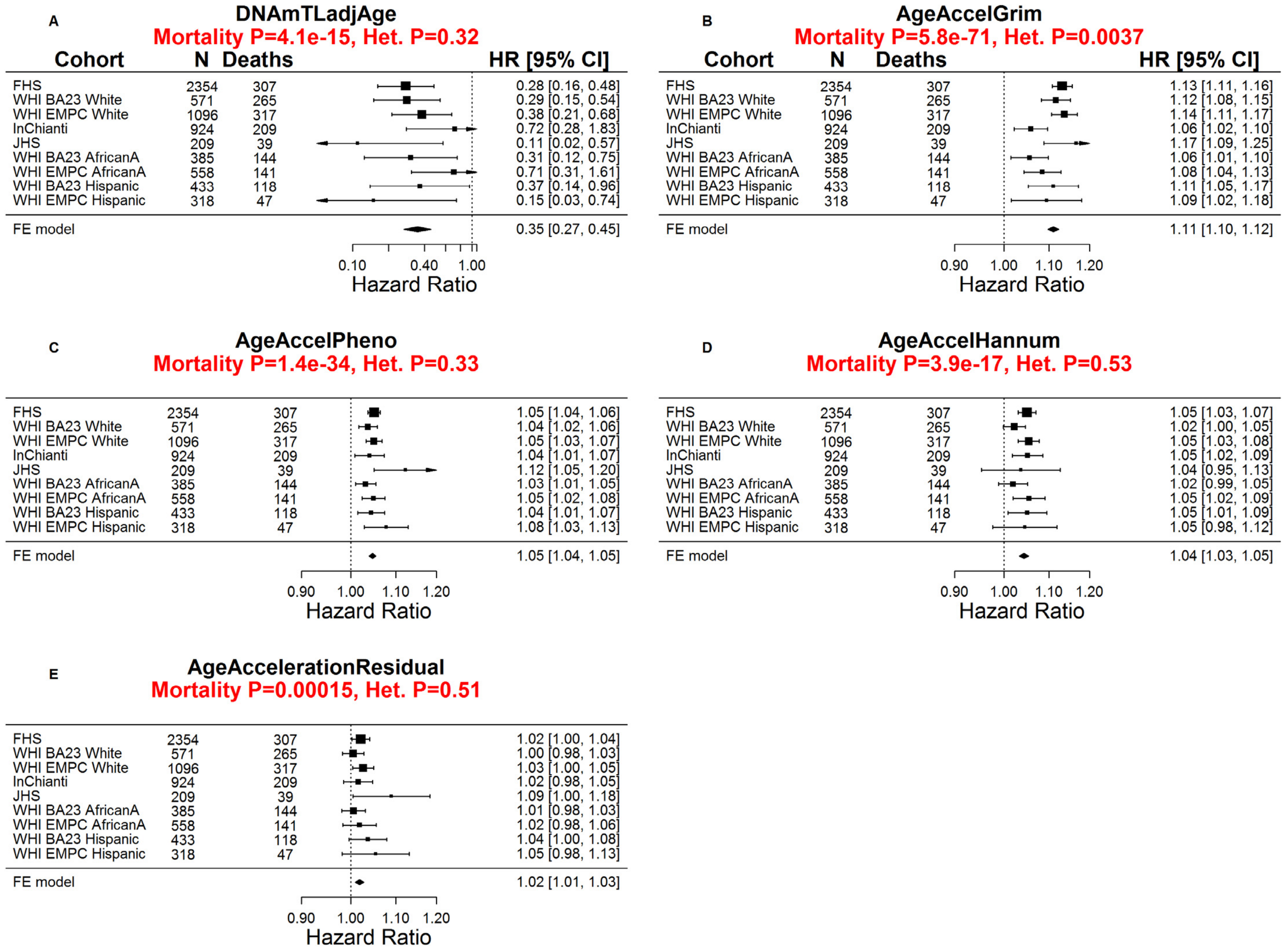

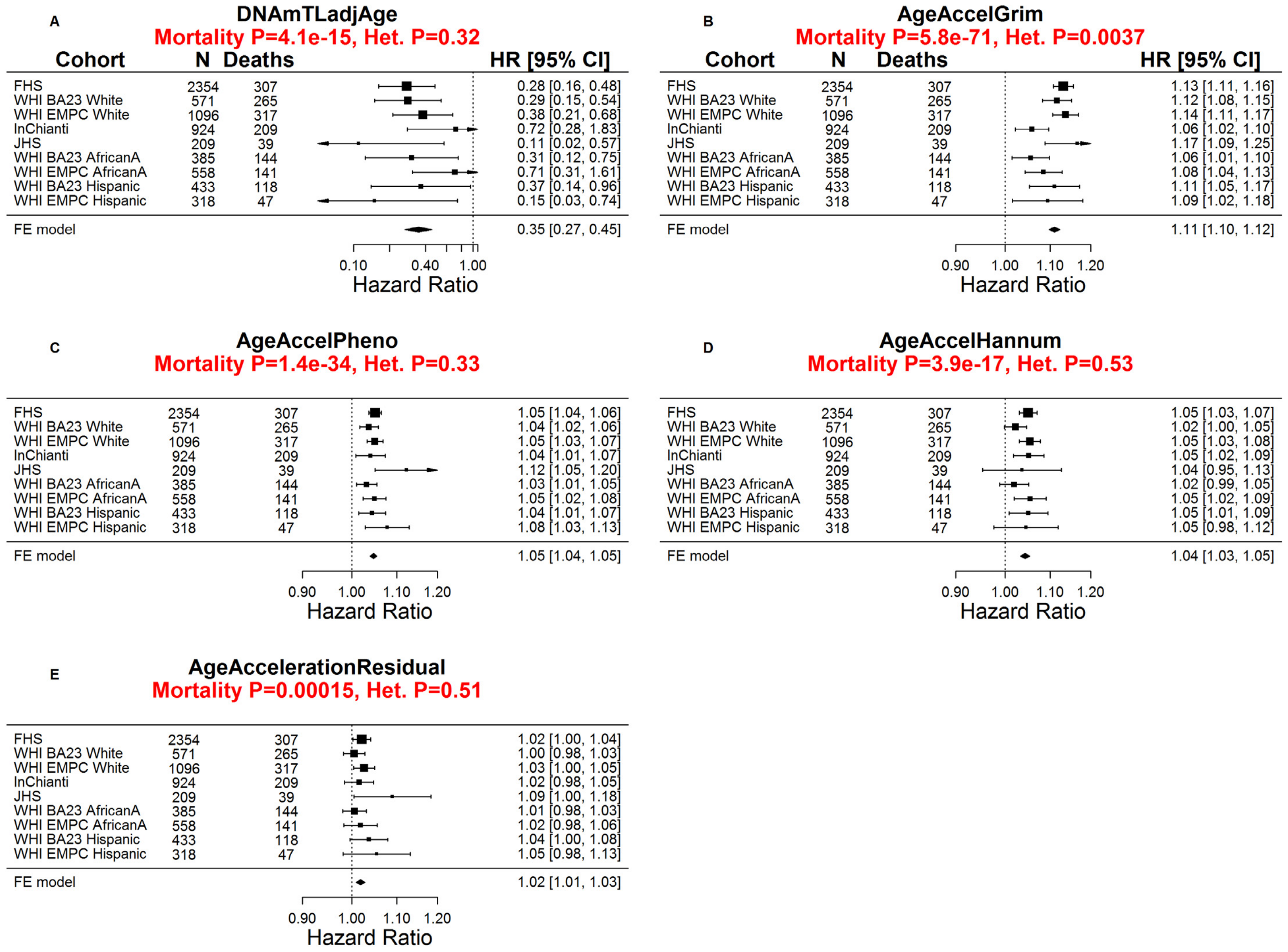
A
DNAmTLadjAge
Mortality P=4.1e-15, Het. P=0.32
Cohort N Deaths HR [95% CI]
FHS 2354 307 0.28 [0.16, 0.48]
WHI BA23 White 571 265 0.29 [0.15, 0.54]
WHI EMPC White 1096 317 0.38 [0.21, 0.68]
InChianti 924 209 0.72 [0.28, 1.83]
JHS 209 39 0.11 [0.02, 0.57]
WHI BA23 AfricanA 385 144 0.31 [0.12, 0.75]
WHI EMPC AfricanA 558 141 0.71 [0.31, 1.61]
WHI BA23 Hispanic 433 118 0.37 [0.14, 0.96]
WHI EMPC Hispanic 318 47 0.15 [0.03, 0.74]
FE model 0.35 [0.27, 0.45]
0.10 0.40 1.00
Hazard Ratio
B
AgeAccelGrim
Mortality P=5.8e-71, Het. P=0.0037
Cohort N Deaths HR [95% CI]
FHS 2354 307 1.13 [1.11, 1.16]
WHI BA23 White 571 265 1.12 [1.08, 1.15]
WHI EMPC White 1096 317 1.14 [1.11, 1.17]
InChianti 924 209 1.06 [1.02, 1.10]
JHS 209 39 1.17 [1.09, 1.25]
WHI BA23 AfricanA 385 144 1.06 [1.01, 1.10]
WHI EMPC AfricanA 558 141 1.08 [1.04, 1.13]
WHI BA23 Hispanic 433 118 1.11 [1.05, 1.17]
WHI EMPC Hispanic 318 47 1.09 [1.02, 1.18]
FE model 1.11 [1.10, 1.12]
0.90 1.00 1.10 1.20
Hazard Ratio
C
AgeAccelPheno
Mortality P=1.4e-34, Het. P=0.33
FHS 2354 307 1.05 [1.04, 1.06]
WHI BA23 White 571 265 1.04 [1.02, 1.06]
WHI EMPC White 1096 317 1.05 [1.03, 1.07]
InChianti 924 209 1.04 [1.01, 1.07]
JHS 209 39 1.12 [1.05, 1.20]
WHI BA23 AfricanA 385 144 1.03 [1.01, 1.05]
WHI EMPC AfricanA 558 141 1.05 [1.02, 1.08]
WHI BA23 Hispanic 433 118 1.04 [1.01, 1.07]
WHI EMPC Hispanic 318 47 1.08 [1.03, 1.13]
FE model 1.05 [1.04, 1.05]
0.90 1.00 1.10 1.20
Hazard Ratio
D
AgeAccelHannum
Mortality P=3.9e-17, Het. P=0.53
FHS 2354 307 1.05 [1.03, 1.07]
WHI BA23 White 571 265 1.02 [1.00, 1.05]
WHI EMPC White 1096 317 1.05 [1.03, 1.08]
InChianti 924 209 1.05 [1.02, 1.09]
JHS 209 39 1.04 [0.95, 1.13]
WHI BA23 AfricanA 385 144 1.02 [0.99, 1.05]
WHI EMPC AfricanA 558 141 1.05 [1.02, 1.09]
WHI BA23 Hispanic 433 118 1.05 [1.01, 1.09]
WHI EMPC Hispanic 318 47 1.05 [0.98, 1.12]
FE model 1.04 [1.03, 1.05]
0.90 1.00 1.10 1.20
Hazard Ratio
E
AgeAccelerationResidual
Mortality P=0.00015, Het. P=0.51
FHS 2354 307 1.02 [1.00, 1.04]
WHI BA23 White 571 265 1.00 [0.98, 1.03]
WHI EMPC White 1096 317 1.03 [1.00, 1.05]
InChianti 924 209 1.02 [0.98, 1.05]
JHS 209 39 1.09 [1.00, 1.18]
WHI BA23 AfricanA 385 144 1.01 [0.98, 1.03]
WHI EMPC AfricanA 558 141 1.02 [0.98, 1.06]
WHI BA23 Hispanic 433 118 1.04 [1.00, 1.08]
WHI EMPC Hispanic 318 47 1.05 [0.98, 1.13]
FE model 1.02 [1.01, 1.03]
0.90 1.00 1.10 1.20
Hazard Ratio

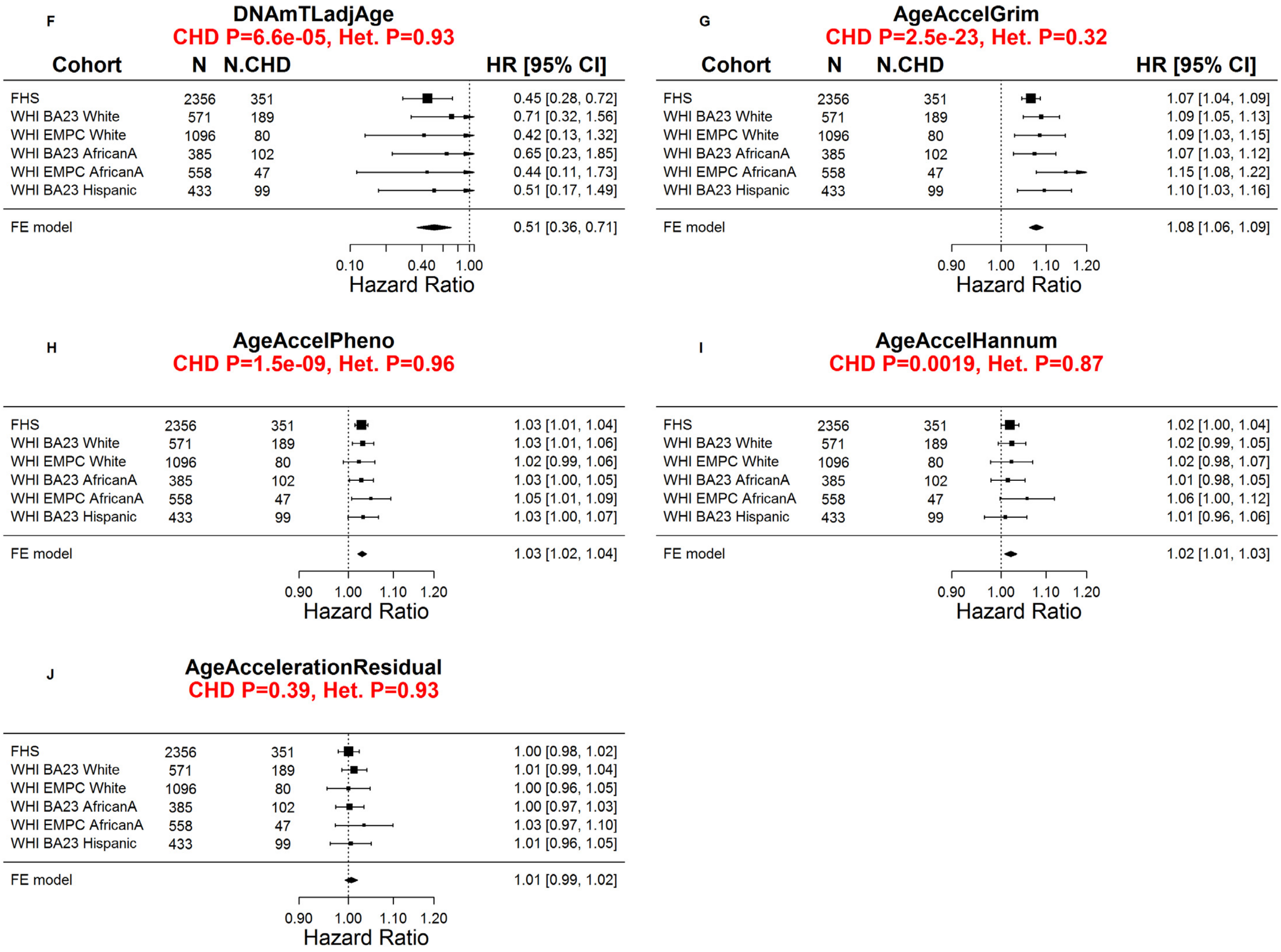
F
DNAmTLadjAge
CHD P=6.6e-05, Het. P=0.93
Cohort N N.CHD HR [95% CI]
FHS 2356 351 0.45 [0.28, 0.72]
WHI BA23 White 571 189 0.71 [0.32, 1.56]
WHI EMPC White 1096 80 0.42 [0.13, 1.32]
WHI BA23 AfricanA 385 102 0.65 [0.23, 1.85]
WHI EMPC AfricanA 558 47 0.44 [0.11, 1.73]
WHI BA23 Hispanic 433 99 0.51 [0.17, 1.49]
FE model 0.51 [0.36, 0.71]
0.10 0.40 1.00
Hazard Ratio
G
AgeAccelGrim
CHD P=2.5e-23, Het. P=0.32
Cohort N N.CHD HR [95% CI]
FHS 2356 351 1.07 [1.04, 1.09]
WHI BA23 White 571 189 1.09 [1.05, 1.13]
WHI EMPC White 1096 80 1.09 [1.03, 1.15]
WHI BA23 AfricanA 385 102 1.07 [1.03, 1.12]
WHI EMPC AfricanA 558 47 1.15 [1.08, 1.22]
WHI BA23 Hispanic 433 99 1.10 [1.03, 1.16]
FE model 1.08 [1.06, 1.09]
0.90 1.00 1.10 1.20
Hazard Ratio
H
AgeAccelPheno
CHD P=1.5e-09, Het. P=0.96
FHS 2356 351 1.03 [1.01, 1.04]
WHI BA23 White 571 189 1.03 [1.01, 1.06]
WHI EMPC White 1096 80 1.02 [0.99, 1.06]
WHI BA23 AfricanA 385 102 1.03 [1.00, 1.05]
WHI EMPC AfricanA 558 47 1.05 [1.01, 1.09]
WHI BA23 Hispanic 433 99 1.03 [1.00, 1.07]
FE model 1.03 [1.02, 1.04]
0.90 1.00 1.10 1.20
Hazard Ratio
I
AgeAccelHannum
CHD P=0.0019, Het. P=0.87
FHS 2356 351 1.02 [1.00, 1.04]
WHI BA23 White 571 189 1.02 [0.99, 1.05]
WHI EMPC White 1096 80 1.02 [0.98, 1.07]
WHI BA23 AfricanA 385 102 1.01 [0.98, 1.05]
WHI EMPC AfricanA 558 47 1.06 [1.00, 1.12]
WHI BA23 Hispanic 433 99 1.01 [0.96, 1.06]
FE model 1.02 [1.01, 1.03]
0.90 1.00 1.10 1.20
Hazard Ratio
J
AgeAccelerationResidual
CHD P=0.39, Het. P=0.93
FHS 2356 351 1.00 [0.98, 1.02]
WHI BA23 White 571 189 1.01 [0.99, 1.04]
WHI EMPC White 1096 80 1.00 [0.96, 1.05]
WHI BA23 AfricanA 385 102 1.00 [0.97, 1.03]
WHI EMPC AfricanA 558 47 1.03 [0.97, 1.10]
WHI BA23 Hispanic 433 99 1.01 [0.96, 1.05]
FE model 1.01 [0.99, 1.02]
0.90 1.00 1.10 1.20
Hazard Ratio

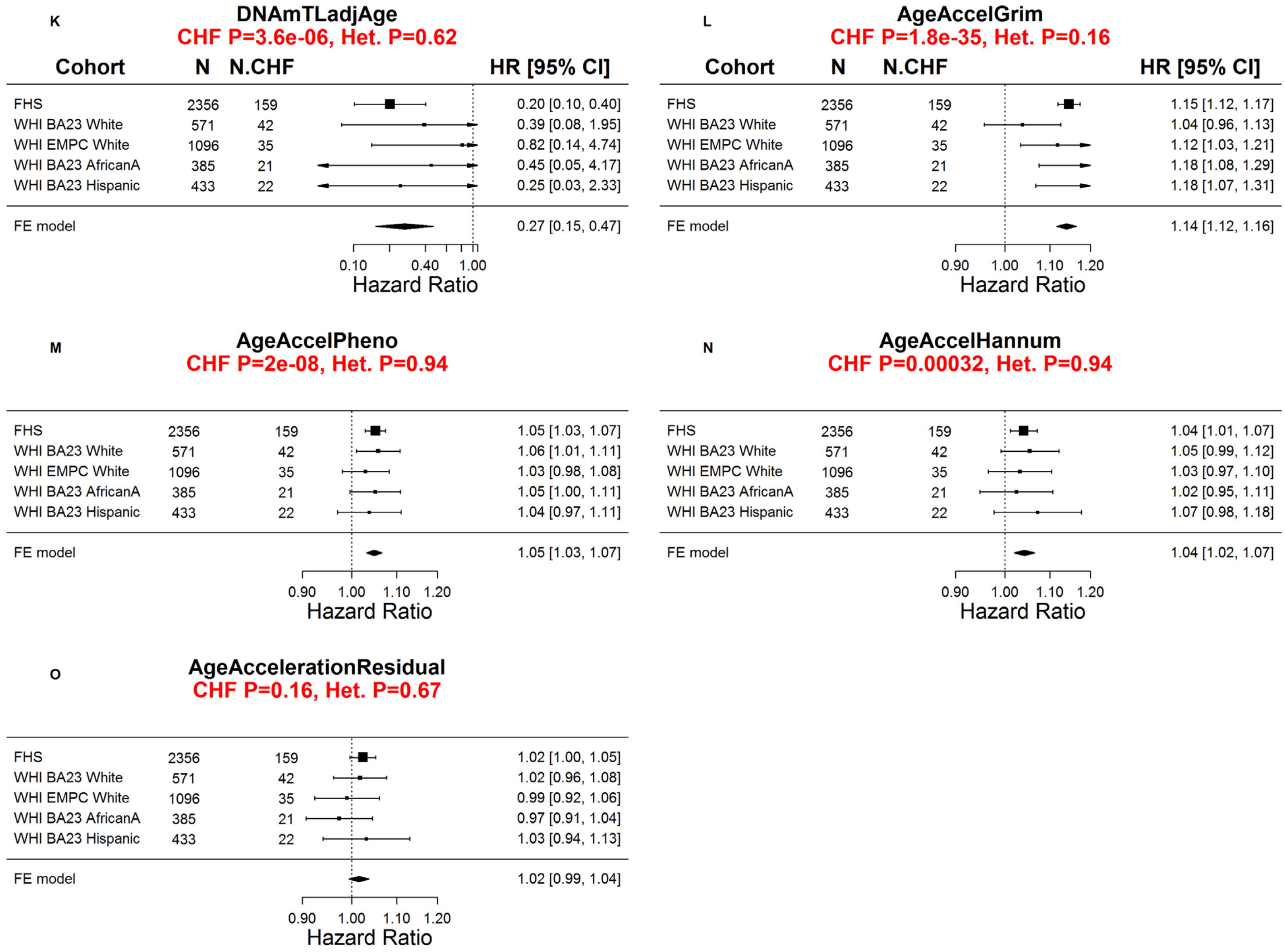
K
DNAmTLadjAge
CHF P=3.6e-06, Het. P=0.62
Cohort N N.CHF HR [95% CI]
FHS 2356 159 0.20 [0.10, 0.40]
WHI BA23 White 571 42 0.39 [0.08, 1.95]
WHI EMPC White 1096 35 0.82 [0.14, 4.74]
WHI BA23 AfricanA 385 21 0.45 [0.05, 4.17]
WHI BA23 Hispanic 433 22 0.25 [0.03, 2.33]
FE model 0.27 [0.15, 0.47]
0.10 0.40 1.00
Hazard Ratio
L
AgeAccelGrim
CHF P=1.8e-35, Het. P=0.16
Cohort N N.CHF HR [95% CI]
FHS 2356 159 1.15 [1.12, 1.17]
WHI BA23 White 571 42 1.04 [0.96, 1.13]
WHI EMPC White 1096 35 1.12 [1.03, 1.21]
WHI BA23 AfricanA 385 21 1.18 [1.08, 1.29]
WHI BA23 Hispanic 433 22 1.18 [1.07, 1.31]
FE model 1.14 [1.12, 1.16]
0.90 1.00 1.10 1.20
Hazard Ratio
M
AgeAccelPheno
CHF P=2e-08, Het. P=0.94
FHS 2356 159 1.05 [1.03, 1.07]
WHI BA23 White 571 42 1.06 [1.01, 1.11]
WHI EMPC White 1096 35 1.03 [0.98, 1.08]
WHI BA23 AfricanA 385 21 1.05 [1.00, 1.11]
WHI BA23 Hispanic 433 22 1.04 [0.97, 1.11]
FE model 1.05 [1.03, 1.07]
0.90 1.00 1.10 1.20
Hazard Ratio
N
AgeAccelHannum
CHF P=0.00032, Het. P=0.94
FHS 2356 159 1.04 [1.01, 1.07]
WHI BA23 White 571 42 1.05 [0.99, 1.12]
WHI EMPC White 1096 35 1.03 [0.97, 1.10]
WHI BA23 AfricanA 385 21 1.02 [0.95, 1.11]
WHI BA23 Hispanic 433 22 1.07 [0.98, 1.18]
FE model 1.04 [1.02, 1.07]
0.90 1.00 1.10 1.20
Hazard Ratio
O
AgeAccelerationResidual
CHF P=0.16, Het. P=0.67
FHS 2356 159 1.02 [1.00, 1.05]
WHI BA23 White 571 42 1.02 [0.96, 1.08]
WHI EMPC White 1096 35 0.99 [0.92, 1.06]
WHI BA23 AfricanA 385 21 0.97 [0.91, 1.04]
WHI BA23 Hispanic 433 22 1.03 [0.94, 1.13]
FE model 1.02 [0.99, 1.04]
0.90 1.00 1.10 1.20
Hazard Ratio

Sensititivy analayis

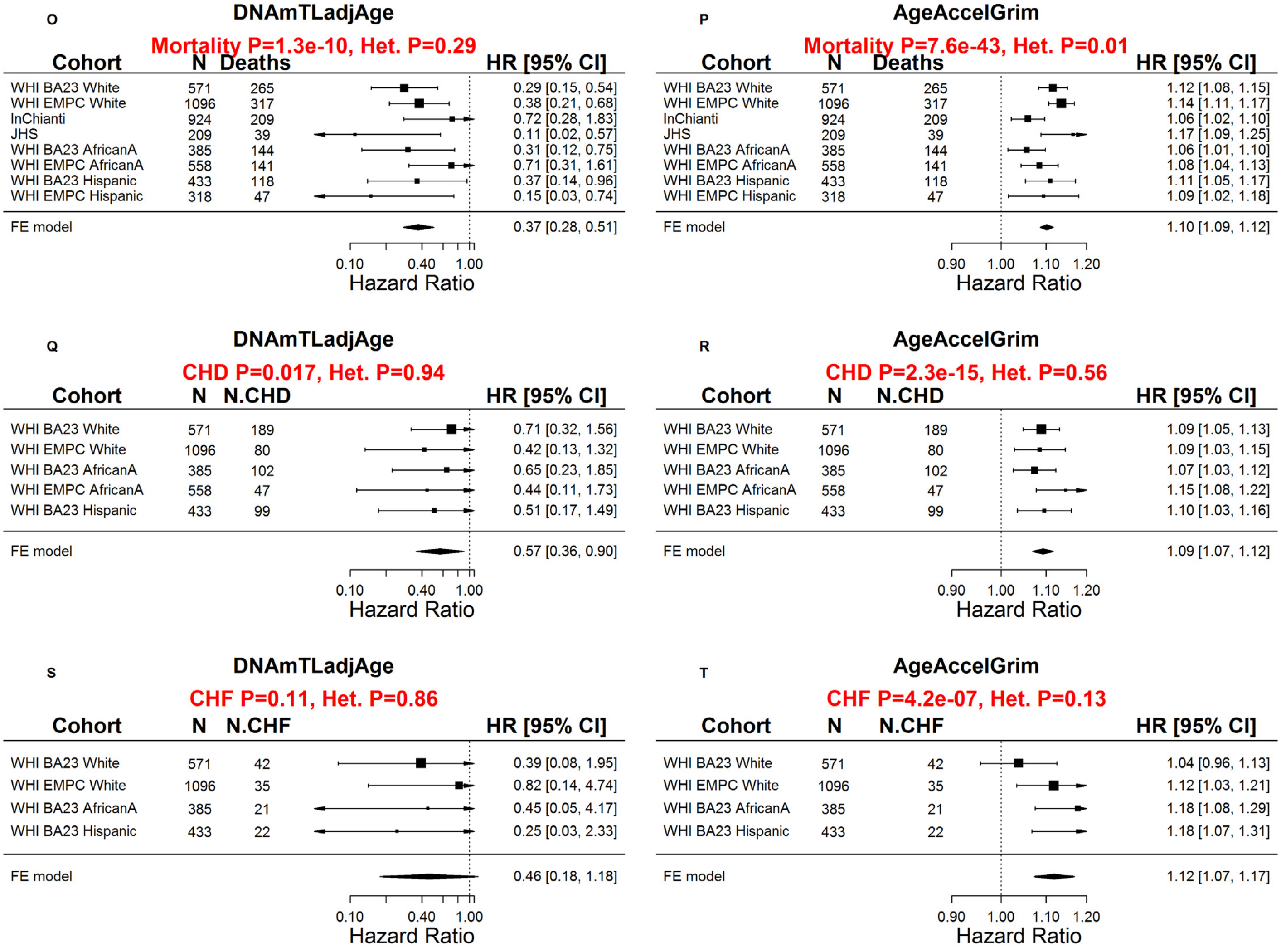

Supplementary Table 1: Direct comparison of DNAmTLadjAge with LTLadjAge for predicting time-to-death in WHI and JHS test data.

We compare age-adjusted DNAmTL (DNAmTLadjAge) with age-adjusted TRF-based leukocyte telomere length (LTLadjAge) for their associations with mortality, using the WHI BA23 test (N=100) and JHS test (N=100) data. The analysis using the WHI BA23 data was stratified by race/ethnicity. Cox regression analysis was performed to predict the hazards of age-adjusted telomere length variables, adjusted for age and gender. Hazard ratios (HR) correspond to one kilobase longer of age-adjusted telomere length.

**Test Data**	**Race**	**Variable**	**Sample Size**	**Number of deaths**	**HR**	**P value**
WHI BA23	AfricanA	DNAmTLadjAge	51	20	0.022	**1.86E-02**
		LTLAdjAge	51	20	1.070	8.78E-01
	European	DNAmTLadjAge	49	20	0.499	5.03E-01
		LTLAdjAge	49	20	0.547	2.34E-01
JHS	AfricanA	DNAmTLadjAge	100	13	0.006	**4.31E-03**
		LTLAdjAge	100	13	0.781	6.02E-01

AfricanA=African American.
P values < 0.05 marked in bold.

Supplementary Table 2: Overview of the cohorts used in the validation analysis

The table summarizes the characteristics of 9,345 individuals from a total of nine independent cohorts across seven studies that were used in our validation analysis. For example, up to two longitudinal measurements were available for each of the 484 individuals from the InChianti cohort.

			Smoking status			
**Cohort**	**N**	**Age**	**Never**	**Former**	**Current**	**Years of Follow-up**
FHS*	2356	66.4±8.97 [40, 92]	9%	51%	40%	7.9+/-1.69 [7.4;8.9]
WHI BA23	1389	65.1±7.25 [50.1, 80.2]	9%	35%	54%	16.6+/-4.87 [14.7;19.9]
WHI EMPC	1972	63.3±7.03 [49.5, 82.0]	52%	38%	9%	18±4.02 [17.9,20.1]
JHS	209	58.2±12.89 [22.9, 84.6]	13%	21%	66%	11.7+/-2.74 [11.1;13.4]
InChianti**	924 (484)	67±16.64 [21, 100]	57%	29%	14%	5.4±4.84 [0.1,9.3]
Twins UK	794	58.23±10.01 [19, 79]	59%	31%	10%	8.9±3.46 [7.0,8.5]
LBC 1921	436	79.1 ±0.6 [77.7, 80.6]	45%	48%	7%	10.0±4.99 [5.9, 14.4]
LBC 1936	906	69.6 ±0.8 [67.6, 71.3]	47%	42%	11%	11.0±2.75 [11.0, 12.7]
BHS	831	42.85±4.60 [28.4, 54.6],	NA	NA	24%	--

-- denotes not applicable; NA denotes not available
Age is presented in the format of mean ± SD [range]
Years of follow-up are presented in the format of mean ±SD [25th, 75th].
*The distribution of age is based on exam 8.
**The statistics are based on the number of 924 observations across 484 individuals.

Supplementary Table 3: Stratification analysis of time-to-death predicted by age-adjusted DNAmTL

Meta analysis for combining hazard ratios (HR) predicting time-to-death based on age-adjusted DNAmTL (DNAmTLadjAge), stratified by different subgroup. At each subgroup of each study set, the analysis was further stratified by racial group that we limited the analysis with number of events ≥20. We list the following columns: group for stratification, stratified level, sample size, number of events, number of data combined in the meta analysis, HR and P values. The categories associated with BMI ranges are a) normal 18.5 -25 (normal), b) 25 to 30 (over), and c) >30 (obese). The categories I to IV associated with education attainment (EDU) are I) less than high school, II) high school degree, III) some college, and IV) college degree and above. The other abbreviations are HYPT (hypertension) and T2D (Type 2 diabetes). Each HR corresponds to one kilobase longer of age-adjusted DNAmTL. The P values < 0.05 are marked in bold.

**Strata**	**Level**	**N**	**Death**	**Number of data**	**HR**	**P**
**Age**	<65years	2884	380	7	0.26	**1.2E-07**
	>=65years	3510	1163	9	0.41	**1.4E-08**
**BMI**	normal	1594	398	7	0.31	**4.4E-05**
	over	2386	493	7	0.37	**1.1E-05**
	obese	2378	599	8	0.46	**7.0E-04**
**EDU**	I	899	276	5	0.42	**2.0E-02**
	II	903	237	3	0.30	**9.3E-05**
	III	1819	475	7	0.48	**3.0E-03**
	IV	1796	343	6	0.26	**7.1E-06**
**HYPT**	No	3717	702	8	0.33	**3.3E-08**
	Yes	2873	820	8	0.42	**1.2E-06**
**T2D**	No	5638	1107	9	0.40	**2.6E-08**
	Yes	690	204	6	0.31	**1.6E-03**
**Cancer**	No	5726	1309	8	0.39	**7.3E-11**
	Yes	675	147	2	0.27	**1.3E-03**

Supplementary Table 4: Stratification analysis of time-to-CHF predicted by age-adjusted DNAmTL

Meta analysis for combining hazard ratios (HR) predicting time-to-CHF based on age-adjusted DNAmTL (DNAmTLadjAge), stratified by different subgroup. At each subgroup of each study set, the analysis was further stratified by racial group that we limited the analysis with number of events ≥20. We list the following columns: group for stratification, stratified level, sample size, number of events, number of data combined in the meta analysis, HR and P values. The categories associated with BMI ranges are a) normal 18.5 -25 (normal), b) 25 to 30 (over), and c) >30 (obese). The categories I to IV associated with education attainment (EDU) are I) less than high school, II) high school degree, III) some college, and IV) college degree and above. The other abbreviations are HYPT (hypertension) and T2D (Type 2 diabetes). Each HR corresponds to one kilobase longer of age-adjusted DNAmTL. The P values < 0.05 are marked in bold.

**Strata**	**Level**	**N**	**N.CHF**	**Number of data**	**HR**	**P**
**Age**	<65years	1064	24	1	0.31	1.8E-01
	>=65years	2232	192	3	0.22	**3.4E-06**
**BMI**	normal	637	31	1	0.33	2.0E-01
	over	956	56	1	0.12	**1.9E-04**
	obese	1369	115	3	0.33	**7.0E-03**
**EDU**	I	--	--	--	--	--
	II	524	43	1	0.19	**3.2E-04**
	III	767	51	2	0.17	**2.0E-02**
	IV	885	46	1	0.09	**2.1E-04**
**HYPT**	No	1123	26	1	0.13	**2.6E-03**
	Yes	1446	158	2	0.23	**5.4E-05**
**T2D**	No	3095	136	2	0.19	**4.0E-06**
	Yes	233	47	1	0.24	6.0E-02
**Cancer**	No	3661	197	4	0.29	**8.0E-04**
	Yes	609	51	1	0.14	**5.0E-05**

--: not available due to number of cases <20 at each subgroup of each study set.

Supplementary Table 5: Stratification analysis of time-to-CHD predicted by age-adjusted DNAmTL

Meta analysis for combining hazard ratios (HR) predicting time-to-CHD based on age-adjusted DNAmTL (DNAmTLadjAge), stratified by different subgroup. At each subgroup of each study set, the analysis was further stratified by racial group that we limited the analysis with number of events ≥20. We list the following columns: group for stratification, stratified level, sample size, number of events, number of data combined in the meta analysis, HR and P values. The categories associated with BMI ranges are a) normal 18.5 -25 (normal), b) 25 to 30 (over), and c) >30 (obese). The categories I to IV associated with education attainment (EDU) are I) less than high school, II) high school degree, III) some college, and IV) college degree and above. The other abbreviations are HYPT (hypertension) and T2D (Type 2 diabetes). Each HR corresponds to one kilobase longer of age-adjusted DNAmTL. The P values < 0.05 are marked in bold.

**Strata**	**Level**	**N**	**N.CHD**	**Number of data**	**HR**	**P**
**Age**	<65years	2657	300	6	0.56	**4.2E-02**
	>=65years	2704	561	6	0.49	**6.5E-04**
**BMI**	normal	1168	154	4	0.34	**7.8E-03**
	over	1690	257	4	0.66	1.7E-01
	obese	2055	392	6	0.50	**8.4E-03**
**EDU**	I	196	52	2	0.54	4.1E-01
	II	660	145	2	0.62	1.9E-01
	III	1719	287	6	0.60	1.3E-01
	IV	1169	170	3	0.44	**2.2E-02**
**HYPT**	No	2624	291	5	0.34	**1.9E-04**
	Yes	2459	554	6	0.68	6.0E-02
**T2D**	No	4373	560	6	0.51	**1.3E-03**
	Yes	318	108	2	0.50	1.8E-01
**Cancer**	No	4587	707	6	0.45	**4.1E-05**
	Yes	609	107	1	0.69	3.4E-01

Supplementary Table 6. Hypergeometric tests of the 140 DNAm TL CpGs enriched with telomere regions

The table below presents enrichment analysis for evaluating the overlap between the 140 CpGs comprising our DNAm TL model and the CpGs nearby telomere regions from 3 mega base (Mb) at each chromosome tail. We list the following columns: region threshold, the number of CpGs located nearby telomeres (**N_{CpG-TL}**), the proportion of **N_{CpG-TL}** divided by total number of whole genome CpGs (N=4,53,093) present in both 450k and Epic array, and hypergeometric P values. We also performed sensitivity analysis based on a variety of regions thresholded at 2, 4, 6 and 8 Mb respectively.

Region (Mb)	N_{CpG-TL}	Proportion	$N_{overlap}$	Hypergeometric P
2	50230	0.11	26	5.9E-03
**3**	**67523**	**0.15**	**32**	**8.1E-03**
4	81835	0.18	34	3.9E-02
6	101312	0.22	44	8.3E-03
8	117269	0.26	48	1.7E-02
10	132541	0.29	52	2.7E-02

Supplementary Table 7: GWAS of DNAmTLadjAge on Leukocyte telomeres associated loci

The table relates genome-wide significant associated SNPs of leukocyte telomere length (LTL) to age-adjusted DNAmTL (DNAmTLadjAge). We presented 14 SNPs associations across 10 distinct susceptibility loci associated with LTL from three large-scale studies: (I) meta-analysis association of LTL in chromosome 5 *TERT* only (N=53,724)[40], (II) a genome-wide meta-analysis of LTL (N=37,684)[41], and (III) a genome-wide meta-analysis of LTL (N=26,089)[42]. Each row presents a genome-wide significant locus associated with LTL in a given study, except chromosome 16 *MPHOSPH6* and chromosome 20 *BCL2L1* just slightly below genome-wide significance and highlighted by the corresponding studies as major findings. The listed markers are the leading SNPs with the most significant P values associated with LTL at a given study and locus, sorted by chromosome and position. The table presents SNP associations for DNAmTLadjAge and age-adjusted LTL (LTLadjAge) respectively, using FHS cohort (N=811). The association analysis was based on linear mixed analysis with the random covariance matrix determined by Kinship coefficients for pedigree structure, adjusted for gender and three principle components as fixed effects.

					**DNAmTLadjAge**		**LTLadjAge**	
**Study**	**SNP**	**bp**	**Gene**	**A1**	**β**	**P**	**β**	**P**
II	rs11125529	54475866	ACYP2	A	1.94E-02	1.67E-01	7.52E-02	5.60E-02
III	rs6772228	58376019	PXK	A	-2.05E-02	3.40E-01	-6.97E-02	2.47E-01
II	rs10936599	169492101	TERC	T	-8.97E-03	4.22E-01	-7.46E-02	**1.69E-02**
III	rs1317082	169497585	TERC	G	-9.02E-03	4.19E-01	-7.39E-02	**1.79E-02**
II	rs7675998	164007820	NAF1	G	4.50E-03	7.11E-01	7.06E-02	**3.83E-02**
III	rs7726159	1282319	TERT	A	2.44E-03	8.44E-01	1.03E-01	**3.14E-03**
I	rs7705526	1285974	TERT	A	-5.02E-03	6.89E-01	9.45E-02	**7.03E-03**
II	rs2736100	1286516	TERT	A	1.59E-03	8.91E-01	-7.08E-02	**2.95E-02**
III	rs2487999	105659826	OBFC1	C	-1.92E-02	2.28E-01	-1.46E-01	**1.01E-03**
II	rs9420907	105676465	OBFC1	A	-9.09E-03	4.99E-01	-1.18E-01	**1.67E-03**
II	rs2967374	82209861	MPHOSPH6	G	3.80E-03	7.37E-01	-4.47E-02	1.59E-01
II	rs8105767	22215441	ZNF208	G	5.85E-03	5.80E-01	4.85E-02	1.02E-01
III	rs6060627	30262159	BCL2L1	T	-3.20E-03	7.52E-01	2.22E-02	4.35E-01
II	rs755017	62421622	RTEL1	G	-1.49E-02	3.13E-01	-1.86E-02	6.54E-01

P values < 0.05 marked in bold.
bp based on Hg19 assembly.

Supplementary references

1 Dawber, T. R., Meadors, G. F. & Moore, F. E., Jr. Epidemiological approaches to heart disease: the Framingham Study. *Am J Public Health Nations Health* **41**, 279-281 (1951).

2 Kannel, W. B., Feinleib, M., McNamara, P. M., Garrison, R. J. & Castelli, W. P. An investigation of coronary heart disease in families. The Framingham offspring study. *Am J Epidemiol* **110**, 281-290 (1979).

3 Aryee, M. J. *et al.* Minfi: a flexible and comprehensive Bioconductor package for the analysis of Infinium DNA methylation microarrays. *Bioinformatics* **30**, 1363-1369, doi:10.1093/bioinformatics/btu049 (2014).

4 Anonymous, A. Design of the Women's Health Initiative clinical trial and observational study. The Women's Health Initiative Study Group. *Control Clin Trials* **19**, 61-109 (1998).

5 Anderson, G. *et al.* Implementation of the women's health initiative study design. *Ann Epidemiol* **13**, S5-17 (2003).

6 Whitsel, E. *Epigenetic Mechanisms of PM-Mediated CVD Risk (WHI-EMPC; R01-ES020836)*, <https://projectreporter.nih.gov/project_info_description.cfm?aid=9054857&icde=40451534&ddparam=&ddvalue=&ddsub=&cr=1&csb=default&cs=ASC&pball=> (2016).

7 Teschendorff, A. E. *et al.* A beta-mixture quantile normalization method for correcting probe design bias in Illumina Infinium 450 k DNA methylation data. *Bioinformatics* **29**, 189-196, doi:10.1093/bioinformatics/bts680 (2013).

8 Johnson, W. E., Rabinovic, A. & Li, C. Adjusting batch effects in microarray expression data using Empirical Bayes methods. *Biostatistics* **8**, 118-127 (2007).

9 Patterson, R. E. *et al.* Measurement characteristics of the Women's Health Initiative food frequency questionnaire. *Annals of epidemiology* **9**, 178-187 (1999).

10 Taylor, H. A., Jr. *et al.* Toward resolution of cardiovascular health disparities in African Americans: design and methods of the Jackson Heart Study. *Ethn Dis* **15**, S6-4-17 (2005).

11 Moore, A. Z. *et al.* Change in Epigenome-Wide DNA Methylation Over 9 Years and Subsequent Mortality: Results From the InCHIANTI Study. *The journals of gerontology. Series A, Biological sciences and medical sciences*, doi:10.1093/gerona/glv118 (2015).

12 Deary, I. J., Gow, A. J., Pattie, A. & Starr, J. M. Cohort profile: the Lothian Birth Cohorts of 1921 and 1936. *Int J Epidemiol* **41**, 1576-1584, doi:10.1093/ije/dyr197 (2012).

13 Taylor, A. M., Pattie, A. & Deary, I. J. Cohort Profile Update: The Lothian Birth Cohorts of 1921 and 1936. *Int J Epidemiol* **47**, 1042-1042r, doi:10.1093/ije/dyy022 (2018).

14 Martin-Ruiz, C. *et al.* Stochastic variation in telomere shortening rate causes heterogeneity of human fibroblast replicative life span. *J Biol Chem* **279**, 17826-17833, doi:10.1074/jbc.M311980200 (2004).

15 Harris, S. E. *et al.* Longitudinal telomere length shortening and cognitive and physical decline in later life: The Lothian Birth Cohorts 1936 and 1921. *Mech Ageing Dev* **154**, 43-48, doi:10.1016/j.mad.2016.02.004 (2016).

16 Shah, S. *et al.* Genetic and environmental exposures constrain epigenetic drift over the human life course. *Genome Res* **24**, 1725-1733, doi:10.1101/gr.176933.114 (2014).

17 Triche, T. J., Weisenberger, D. J., Van Den Berg, D., Laird, P. W. & Siegmund, K. D. Low-level processing of Illumina Infinium DNA Methylation BeadArrays. *Nucleic acids research* **41**, e90-e90, doi:10.1093/nar/gkt090 (2013).

18 Jia, X., Craig, L. C., Aucott, L. S., Milne, A. C. & McNeill, G. Repeatability and validity of a food frequency questionnaire in free-living older people in relation to cognitive function. *J Nutr Health Aging* **12**, 735-741 (2008).

19 Moayyeri, A., Hammond, C. J., Hart, D. J. & Spector, T. D. The UK Adult Twin Registry (TwinsUK Resource). *Twin Res Hum Genet* **16**, 144-149, doi:10.1017/thg.2012.89 (2013).

20 Berenson, G. S. & Bogalusa Heart Study, I. Bogalusa Heart Study: a long-term community study of a rural biracial (Black/White) population. *Am J Med Sci* **322**, 293-300 (2001).

21 Aviv, A. *et al.* Leukocyte telomere dynamics: longitudinal findings among young adults in the Bogalusa Heart Study. *Am J Epidemiol* **169**, 323-329, doi:10.1093/aje/kwn338 (2009).

22 Pidsley, R. *et al.* A data-driven approach to preprocessing Illumina 450K methylation array data. *BMC Genomics* **14**, 293, doi:10.1186/1471-2164-14-293 (2013).

23 Grundberg, E. *et al.* Global Analysis of DNA Methylation Variation in Adipose Tissue from Twins Reveals Links to Disease-Associated Variants in Distal Regulatory Elements. *The American Journal of Human Genetics* **93**, 876-890, doi:10.1016/j.ajhg.2013.10.004 (2013).

24 Horvath, S. *et al.* Obesity accelerates epigenetic aging of human liver. *Proceedings of the National Academy of Sciences of the United States of America* **111**, 15538-15543, doi:10.1073/pnas.1412759111 (2014).

25 Walker, R. F. *et al.* Epigenetic age analysis of children who seem to evade aging. *Aging* **7**, 334-339, doi:10.18632/aging.100744 (2015).

26 Ahrens, M. *et al.* DNA methylation analysis in nonalcoholic fatty liver disease suggests distinct disease-specific and remodeling signatures after bariatric surgery. *Cell Metab* **18**, 296-302, doi:10.1016/j.cmet.2013.07.004 (2013).

27 Reynolds, L. M. *et al.* Age-related variations in the methylome associated with gene expression in human monocytes and T cells. *Nat Commun* **5**, 5366, doi:10.1038/ncomms6366 (2014).
28 Houseman, E. *et al.* DNA methylation arrays as surrogate measures of cell mixture distribution. *BMC bioinformatics* **13**, 86 (2012).
29 Horvath, S. DNA methylation age of human tissues and cell types. *Genome biology* **14**, R115, doi:10.1186/gb-2013-14-10-r115 (2013).
30 Horvath, S. & Levine, A. J. HIV-1 Infection Accelerates Age According to the Epigenetic Clock. *J Infect Dis* **212**, 1563-1573, doi:10.1093/infdis/jiv277 (2015).
31 Horvath, S. *et al.* An epigenetic clock analysis of race/ethnicity, sex, and coronary heart disease. *Genome biology* **17**, 171, doi:10.1186/s13059-016-1030-0 (2016).
32 Horvath, S. *et al.* Epigenetic clock for skin and blood cells applied to Hutchinson Gilford Progeria Syndrome and ex vivo studies. *Aging* **10**, 1758-1775, doi:10.18632/aging.101508 (2018).
33 Ahrens, M. *et al.* DNA Methylation Analysis in Nonalcoholic Fatty Liver Disease Suggests Distinct Disease-Specific and Remodeling Signatures after Bariatric Surgery. *Cell Metabolism* **18**, 296-302, doi:10.1016/j.cmet.2013.07.004 (2013).
34 Reynolds, L. M. *et al.* Age-related variations in the methylome associated with gene expression in human monocytes and T cells. *Nat Commun* **5**, 5366, doi:10.1038/ncomms6366 (2014).
35 Langfelder, P. & Horvath, S. WGCNA: an R package for weighted correlation network analysis. *BMC bioinformatics* **9**, 559, doi:10.1186/1471-2105-9-559 (2008).
36 Horvath, S. & Levine, A. J. HIV-1 infection accelerates age according to the epigenetic clock. *J Infect Dis*, doi:10.1093/infdis/jiv277 (2015).
37 Lu, A. T., Quach, A., Ferrucci, L., Assimes, T. & Horvath, S. DNA methylation GrimAge strongly predicts lifespan and healthspan. *accepted by Aging* (2019).
38 Levine, M. E. *et al.* An epigenetic biomarker of aging for lifespan and healthspan. *Aging* **10**, 573-591, doi:10.18632/aging.101414 (2018).
39 Hannum, G. *et al.* Genome-wide methylation profiles reveal quantitative views of human aging rates. *Molecular cell* **49**, 359-367, doi:10.1016/j.molcel.2012.10.016 (2013).
40 Bojesen, S. E. *et al.* Multiple independent variants at the TERT locus are associated with telomere length and risks of breast and ovarian cancer. *Nature genetics* **45**, 371-384, 384e371-372, doi:10.1038/ng.2566 (2013).
41 Codd, V. *et al.* Identification of seven loci affecting mean telomere length and their association with disease. *Nature genetics* **45**, 422-427, 427e421-422, doi:10.1038/ng.2528 (2013).
42 Pooley, K. A. *et al.* A genome-wide association scan (GWAS) for mean telomere length within the COGS project: identified loci show little association with hormone-related cancer risk. *Human molecular genetics* **22**, 5056-5064, doi:10.1093/hmg/ddt355 (2013).
