# Supplementary material for: DNA methylation-based estimator of telomere length
Source: Aging (Albany NY). 2019 Aug 18;11(16):5895–923. doi: 10.18632/aging.102173 (PMC6738410; doi:10.18632/aging.102173)
Supplement: Supplementary Tables [file aging-11-102173-s009.pdf]

SUPPLEMENTARY TABLES

Supplementary Table 1. Direct comparison of DNAmTLadjAge with LTLadjAge for predicting time-to-death in WHI and JHS test data.

We compare age-adjusted DNAmTL (DNAmTLadjAge) with age-adjusted TRF-based leukocyte telomere length (LTLadjAge) for their associations with mortality, using the WHI BA23 test (N=100) and JHS test (N=100) data. The analysis using the WHI BA23 data was stratified by race/ethnicity. Cox regression analysis was performed to predict the hazards of age-adjusted telomere length variables, adjusted for age and gender. Hazard ratios (HR) correspond to one kilobase longer of age-adjusted telomere length.

Test Data	Race	Variable	Sample Size	Number of deaths	HR	P value
WHI BA23	AfricanA	DNAmTLadjAge	51	20	0.022	**1.86E-02**
		LTLAdjAge	51	20	1.070	8.78E-01
	European	DNAmTLadjAge	49	20	0.499	5.03E-01
		LTLAdjAge	49	20	0.547	2.34E-01
JHS	AfricanA	DNAmTLadjAge	100	13	0.006	**4.31E-03**
		LTLAdjAge	100	13	0.781	6.02E-01

AfricanA=African American.
P values < 0.05 marked in bold.

Supplementary Table 2. Overview of the cohorts used in the validation analysis.

The table summarizes the characteristics of 9,345 individuals from a total of nine independent cohorts across seven studies that were used in our validation analysis. For example, up to two longitudinal measurements were available for each of the 484 individuals from the InChianti cohort.

			Smoking status			
Cohort	N	Age	Never	Former	Current	Years of Follow-up
FHS*	2356	66.4±8.97 [40, 92]	9%	51%	40%	7.9+/-1.69 [7.4;8.9]
WHI BA23	1389	65.1±7.25 [50.1, 80.2]	9%	35%	54%	16.6+/-4.87 [14.7;19.9]
WHI EMPC	1972	63.3±7.03 [49.5, 82.0]	52%	38%	9%	18±4.02 [17.9,20.1]
JHS	209	58.2±12.89 [22.9, 84.6]	13%	21%	66%	11.7+/-2.74 [11.1;13.4]
InChianti**	924 (484)	67±16.64 [21, 100]	57%	29%	14%	5.4±4.84 [0.1,9.3]
Twins UK	794	58.23±10.01 [19, 79]	59%	31%	10%	8.9±3.46 [7.0,8.5]
LBC 1921	436	79.1 ±0.6 [77.7, 80.6]	45%	48%	7%	10.0±4.99 [5.9, 14.4]
LBC 1936	906	69.6 ±0.8 [67.6, 71.3]	47%	42%	11%	11.0±2.75 [11.0, 12.7]
BHS	831	42.85±4.60 [28.4, 54.6],	NA	NA	24%	--

-- denotes not applicable; NA denotes not available
Age is presented in the format of mean ± SD [range]
Years of follow-up are presented in the format of mean ±SD [25th, 75th].
*The distribution of age is based on exam 8.
**The statistics are based on the number of 924 observations across 484 individuals.

Supplementary Table 3. Stratification analysis of time-to-death predicted by age-adjusted DNAmTL.

Meta analysis for combining hazard ratios (HR) predicting time-to-death based on age-adjusted DNAmTL (DNAmTLadjAge), stratified by different subgroup. At each subgroup of each study set, the analysis was further stratified by racial group that we limited the analysis with number of events ≥20. We list the following columns: group for stratification, stratified level, sample size, number of events, number of data combined in the meta analysis, HR and P values. The categories associated with BMI ranges are a) normal 18.5 -25 (normal), b) 25 to 30 (over), and c) >30 (obese). The categories I to IV associated with education attainment (EDU) are I) less than high school, II) high school degree, III) some college, and IV) college degree and above. The other abbreviations are HYPT (hypertension) and T2D (Type 2 diabetes). Each HR corresponds to one kilobase longer of age-adjusted DNAmTL. The P values < 0.05 are marked in bold.

Strata	Level	N	Death	Number of data	HR	P
**Age**	<65years	2884	380	7	0.26	**1.2E-07**
	>=65years	3510	1163	9	0.41	**1.4E-08**
**BMI**	normal	1594	398	7	0.31	**4.4E-05**
	over	2386	493	7	0.37	**1.1E-05**
	obese	2378	599	8	0.46	**7.0E-04**
**EDU**	I	899	276	5	0.42	**2.0E-02**
	II	903	237	3	0.30	**9.3E-05**
	III	1819	475	7	0.48	**3.0E-03**
	IV	1796	343	6	0.26	**7.1E-06**
**HYPT**	No	3717	702	8	0.33	**3.3E-08**
	Yes	2873	820	8	0.42	**1.2E-06**
**T2D**	No	5638	1107	9	0.40	**2.6E-08**
	Yes	690	204	6	0.31	**1.6E-03**
**Cancer**	No	5726	1309	8	0.39	**7.3E-11**
	Yes	675	147	2	0.27	**1.3E-03**

Supplementary Table 4. Stratification analysis of time-to-CHF predicted by age-adjusted DNAmTL.

Meta analysis for combining hazard ratios (HR) predicting time-to-CHF based on age-adjusted DNAmTL (DNAmTLadjAge), stratified by different subgroup. At each subgroup of each study set, the analysis was further stratified by racial group that we limited the analysis with number of events ≥20. We list the following columns: group for stratification, stratified level, sample size, number of events, number of data combined in the meta analysis, HR and P values. The categories associated with BMI ranges are a) normal 18.5 -25 (normal), b) 25 to 30 (over), and c) >30 (obese). The categories I to IV associated with education attainment (EDU) are I) less than high school, II) high school degree, III) some college, and IV) college degree and above. The other abbreviations are HYPT (hypertension) and T2D (Type 2 diabetes). Each HR corresponds to one kilobase longer of age-adjusted DNAmTL. The P values < 0.05 are marked in bold.

Strata	Level	N	N.CHF	Number of data	HR	P
**Age**	<65years	1064	24	1	0.31	1.8E-01
	>=65years	2232	192	3	0.22	**3.4E-06**
**BMI**	normal	637	31	1	0.33	2.0E-01
	over	956	56	1	0.12	**1.9E-04**
	obese	1369	115	3	0.33	**7.0E-03**
**EDU**	I	--	--	--	--	--
	II	524	43	1	0.19	**3.2E-04**
	III	767	51	2	0.17	**2.0E-02**
	IV	885	46	1	0.09	**2.1E-04**
**HYPT**	No	1123	26	1	0.13	**2.6E-03**
	Yes	1446	158	2	0.23	**5.4E-05**
**T2D**	No	3095	136	2	0.19	**4.0E-06**
	Yes	233	47	1	0.24	6.0E-02
**Cancer**	No	3661	197	4	0.29	**8.0E-04**
	Yes	609	51	1	0.14	**5.0E-05**

--: not available due to number of cases <20 at each subgroup of each study set.

Supplementary Table 5. Stratification analysis of time-to-CHD predicted by age-adjusted DNAmTL.

Meta analysis for combining hazard ratios (HR) predicting time-to-CHD based on age-adjusted DNAmTL (DNAmTLadjAge), stratified by different subgroup. At each subgroup of each study set, the analysis was further stratified by racial group that we limited the analysis with number of events ≥20. We list the following columns: group for stratification, stratified level, sample size, number of events, number of data combined in the meta analysis, HR and P values. The categories associated with BMI ranges are a) normal 18.5 -25 (normal), b) 25 to 30 (over), and c) >30 (obese). The categories I to IV associated with education attainment (EDU) are I) less than high school, II) high school degree, III) some college, and IV) college degree and above. The other abbreviations are HYPT (hypertension) and T2D (Type 2 diabetes). Each HR corresponds to one kilobase longer of age-adjusted DNAmTL. The P values < 0.05 are marked in bold.

Strata	Level	N	N.CHD	Number of data	HR	P
**Age**	<65years	2657	300	6	0.56	**4.2E-02**
	>=65years	2704	561	6	0.49	**6.5E-04**
**BMI**	normal	1168	154	4	0.34	**7.8E-03**
	over	1690	257	4	0.66	1.7E-01
	obese	2055	392	6	0.50	**8.4E-03**
**EDU**	I	196	52	2	0.54	4.1E-01
	II	660	145	2	0.62	1.9E-01
	III	1719	287	6	0.60	1.3E-01
	IV	1169	170	3	0.44	**2.2E-02**
**HYPT**	No	2624	291	5	0.34	**1.9E-04**
	Yes	2459	554	6	0.68	6.0E-02
**T2D**	No	4373	560	6	0.51	**1.3E-03**
	Yes	318	108	2	0.50	1.8E-01
**Cancer**	No	4587	707	6	0.45	**4.1E-05**
	Yes	609	107	1	0.69	3.4E-01

Supplementary Table 6. Hypergeometric tests of the 140 DNAm TL CpGs enriched with telomere regions

The table below presents enrichment analysis for evaluating the overlap between the 140 CpGs comprising our DNAm TL model and the CpGs nearby telomere regions from 3 mega base (Mb) at each chromosome tail. We list the following columns: region threshold, the number of CpGs located nearby telomeres ($N_{CpG\text{-}TL}$), the proportion of $N_{CpG\text{-}TL}$ divided by total number of whole genome CpGs (N=4,53,093) present in both 450k and Epic array, and hypergeometric P values. We also performed sensitivity analysis based on a variety of regions thresholded at 2, 4, 6 and 8 Mb respectively.

Region (Mb)	$N_{CpG\text{-}TL}$	Proportion	$N_{overlap}$	Hypergeometric P
2	50230	0.11	26	5.9E-03
**3**	**67523**	**0.15**	**32**	**8.1E-03**
4	81835	0.18	34	3.9E-02
6	101312	0.22	44	8.3E-03
8	117269	0.26	48	1.7E-02
10	132541	0.29	52	2.7E-02

Supplementary Table 7. GWAS of DNAmTLadjAge on Leukocyte telomeres associated loci.

The table relates genome-wide significant associated SNPs of leukocyte telomere length (LTL) to age-adjusted DNAmTL (DNAmTLadjAge). We presented 14 SNPs associations across 10 distinct susceptibility loci associated with LTL from three large-scale studies: (I) meta-analysis association of LTL in chromosome 5 *TERT* only (N=53,724) [40], (II) a genome-wide meta-analysis of LTL (N=37,684) [41], and (III) a genome-wide meta-analysis of LTL (N=26,089) [42]. Each row presents a genome-wide significant locus associated with LTL in a given study, except chromosome 16 *MPHOSPH6* and chromosome 20 *BCL2L1* just slightly below genome-wide significance and highlighted by the corresponding studies as major findings. The listed markers are the leading SNPs with the most significant P values associated with LTL at a given study and locus, sorted by chromosome and position. The table presents SNP associations for DNAmTLadjAge and age-adjusted LTL (LTLadjAge) respectively, using FHS cohort (N=811). The association analysis was based on linear mixed analysis with the random covariance matrix determined by Kinship coefficients for pedigree structure, adjusted for gender and three principle components as fixed effects.

					DNAmTLadjAge		LTLadjAge	
**Study**	**SNP**	**bp**	**Gene**	**A1**	**β**	**P**	**β**	**P**
II	rs11125529	54475866	ACYP2	A	1.94E-02	1.67E-01	7.52E-02	5.60E-02
III	rs6772228	58376019	PXK	A	-2.05E-02	3.40E-01	-6.97E-02	2.47E-01
II	rs10936599	169492101	TERC	T	-8.97E-03	4.22E-01	-7.46E-02	**1.69E-02**
III	rs1317082	169497585	TERC	G	-9.02E-03	4.19E-01	-7.39E-02	**1.79E-02**
II	rs7675998	164007820	NAF1	G	4.50E-03	7.11E-01	7.06E-02	**3.83E-02**
III	rs7726159	1282319	TERT	A	2.44E-03	8.44E-01	1.03E-01	**3.14E-03**
I	rs7705526	1285974	TERT	A	-5.02E-03	6.89E-01	9.45E-02	**7.03E-03**
II	rs2736100	1286516	TERT	A	1.59E-03	8.91E-01	-7.08E-02	**2.95E-02**
III	rs2487999	105659826	OBFC1	C	-1.92E-02	2.28E-01	-1.46E-01	**1.01E-03**
II	rs9420907	105676465	OBFC1	A	-9.09E-03	4.99E-01	-1.18E-01	**1.67E-03**
II	rs2967374	82209861	MPHOSPH6	G	3.80E-03	7.37E-01	-4.47E-02	1.59E-01
II	rs8105767	22215441	ZNF208	G	5.85E-03	5.80E-01	4.85E-02	1.02E-01
III	rs6060627	30262159	BCL2L1	T	-3.20E-03	7.52E-01	2.22E-02	4.35E-01
II	rs755017	62421622	RTEL1	G	-1.49E-02	3.13E-01	-1.86E-02	6.54E-01

P values < 0.05 marked in bold.
bp based on Hg19 assembly.
