# Supplementary material for: DNA methylation-based estimator of telomere length
Source: Aging (Albany NY). 2019 Aug 18;11(16):5895–923. doi: 10.18632/aging.102173 (PMC6738410; doi:10.18632/aging.102173)
Supplement: Supplementary References [file aging-11-102173-s007.pdf]

SUPPLEMENTARY REFERENCES

1. Dawber TR, Meadors GF, Moore FE Jr. Epidemiological approaches to heart disease: the Framingham Study. Am J Public Health Nations Health. 1951; 41:279–81. https://doi.org/10.2105/AJPH.41.3.279 PMID:14819398

2. Kannel WB, Feinleib M, McNamara PM, Garrison RJ, Castelli WP. An investigation of coronary heart disease in families. The Framingham offspring study. Am J Epidemiol. 1979; 110:281–90. https://doi.org/10.1093/oxfordjournals.aje.a112813 PMID:474565

3. Aryee MJ, Jaffe AE, Corrada-Bravo H, Ladd-Acosta C, Feinberg AP, Hansen KD, Irizarry RA. Minfi: a flexible and comprehensive Bioconductor package for the analysis of Infinium DNA methylation microarrays. Bioinformatics. 2014; 30:1363–69. https://doi.org/10.1093/bioinformatics/btu049 PMID:24478339

4. Anonymous A, and The Women's Health Initiative Study Group. Design of the Women's Health Initiative clinical trial and observational study. Control Clin Trials. 1998; 19:61–109. https://doi.org/10.1016/S0197-2456(97)00078-0 PMID:9492970

5. Anderson GL, Manson J, Wallace R, Lund B, Hall D, Davis S, Shumaker S, Wang CY, Stein E, Prentice RL. Implementation of the women's health initiative study design. Ann Epidemiol. 2003 (Suppl); 13:S5–17. https://doi.org/10.1016/S1047-2797(03)00043-7 PMID:14575938

6. Whitsel E. Epigenetic Mechanisms of PM-Mediated CVD Risk (WHI-EMPC; R01-ES020836). 2016 https://projectreporter.nih.gov/project_info_description.cfm?aid=9054857&icde=40451534&ddparam=&ddvalue=&ddsub=&cr=1&csb=default&cs=ASC&pball=

7. Teschendorff AE, Marabita F, Lechner M, Bartlett T, Tegner J, Gomez-Cabrero D, Beck S. A beta-mixture quantile normalization method for correcting probe design bias in Illumina Infinium 450 k DNA methylation data. Bioinformatics. 2013; 29:189–96. https://doi.org/10.1093/bioinformatics/bts680 PMID:23175756

8. Johnson WE, Li C, Rabinovic A. Adjusting batch effects in microarray expression data using empirical Bayes methods. Biostatistics. 2007; 8:118–27. https://doi.org/10.1093/biostatistics/kxj037 PMID:16632515

9. Patterson RE, Kristal AR, Tinker LF, Carter RA, Bolton MP, Agurs-Collins T. Measurement characteristics of the Women's Health Initiative food frequency questionnaire. Ann Epidemiol. 1999; 9:178–87. https://doi.org/10.1016/S1047-2797(98)00055-6 PMID:10192650

10. Taylor HA Jr, Wilson JG, Jones DW, Sarpong DF, Srinivasan A, Garrison RJ, Nelson C, Wyatt SB. Toward resolution of cardiovascular health disparities in African Americans: design and methods of the Jackson Heart Study. Ethn Dis. 2005 (Suppl 6); 15:S6–4, 17. PMID:16320381

11. Moore AZ, et al. Change in Epigenome-Wide DNA Methylation Over 9 Years and Subsequent Mortality: Results From the InCHIANTI Study. J Gerontol A Biol Sci Med Sci. 2015. 10.1093/gerona/glv118 PMID:26355017

12. Deary IJ, Gow AJ, Pattie A, Starr JM. Cohort profile: the Lothian Birth Cohorts of 1921 and 1936. Int J Epidemiol. 2012; 41:1576–84. https://doi.org/10.1093/ije/dyr197 PMID:22253310

13. Taylor AM, Pattie A, Deary IJ. Cohort Profile Update: The Lothian Birth Cohorts of 1921 and 1936. Int J Epidemiol. 2018; 47:1042–1042r. https://doi.org/10.1093/ije/dyy022 PMID:29546429

14. Martin-Ruiz C, Saretzki G, Petrie J, Ladhoff J, Jeyapalan J, Wei W, Sedivy J, von Zglinicki T. Stochastic variation in telomere shortening rate causes heterogeneity of human fibroblast replicative life span. J Biol Chem. 2004; 279:17826–33. https://doi.org/10.1074/jbc.M311980200 PMID:14963037

15. Harris SE, Marioni RE, Martin-Ruiz C, Pattie A, Gow AJ, Cox SR, Corley J, von Zglinicki T, Starr JM, Deary IJ. Longitudinal telomere length shortening and cognitive and physical decline in later life: The Lothian Birth Cohorts 1936 and 1921. Mech Ageing Dev. 2016; 154:43–48. https://doi.org/10.1016/j.mad.2016.02.004 PMID:26876762

16. Shah S, McRae AF, Marioni RE, Harris SE, Gibson J, Henders AK, Redmond P, Cox SR, Pattie A, Corley J, Murphy L, Martin NG, Montgomery GW, et al. Genetic and environmental exposures constrain epigenetic drift over the human life course. Genome Res. 2014; 24:1725–33. https://doi.org/10.1101/gr.176933.114 PMID:25249537

17. Triche TJ Jr, Weisenberger DJ, Van Den Berg D, Laird PW, Siegmund KD. Low-level processing of Illumina Infinium DNA Methylation BeadArrays. Nucleic Acids Res. 2013; 41:e90–90. https://doi.org/10.1093/nar/gkt090 PMID:23476028

18. Jia X, Craig LC, Aucott LS, Milne AC, McNeill G. Repeatability and validity of a food frequency questionnaire in free-living older people in relation to cognitive function. J Nutr Health Aging. 2008; 12:735–41. PMID:19043649

19. Moayyeri A, Hammond CJ, Hart DJ, Spector TD. The UK Adult Twin Registry (TwinsUK Resource). Twin Res Hum Genet. 2013; 16:144–49. https://doi.org/10.1017/thg.2012.89 PMID:23088889

20. Berenson GS, and Bogalusa Heart Study Investigators. Bogalusa Heart Study: a long-term community study of a rural biracial (Black/White) population. Am J Med Sci. 2001; 322:293–300. https://doi.org/10.1097/00000441-200111000-00007 PMID:11876192

21. Aviv A, Chen W, Gardner JP, Kimura M, Brimacombe M, Cao X, Srinivasan SR, Berenson GS. Leukocyte telomere dynamics: longitudinal findings among young adults in the Bogalusa Heart Study. Am J Epidemiol. 2009; 169:323–29. https://doi.org/10.1093/aje/kwn338 PMID:19056834

22. Pidsley R, Y Wong CC, Volta M, Lunnon K, Mill J, Schalkwyk LC. A data-driven approach to preprocessing Illumina 450K methylation array data. BMC Genomics. 2013; 14:293. https://doi.org/10.1186/1471-2164-14-293 PMID:23631413

23. Grundberg E, Meduri E, Sandling JK, Hedman AK, Keildson S, Buil A, Busche S, Yuan W, Nisbet J, Sekowska M, Wilk A, Barrett A, Small KS, et al, and Multiple Tissue Human Expression Resource Consortium. Global analysis of DNA methylation variation in adipose tissue from twins reveals links to disease-associated variants in distal regulatory elements. Am J Hum Genet. 2013; 93:876–90. https://doi.org/10.1016/j.ajhg.2013.10.004 PMID:24183450

24. Horvath S, Erhart W, Brosch M, Ammerpohl O, von Schönfels W, Ahrens M, Heits N, Bell JT, Tsai PC, Spector TD, Deloukas P, Siebert R, Sipos B, et al. Obesity accelerates epigenetic aging of human liver. Proc Natl Acad Sci USA. 2014; 111:15538–43. https://doi.org/10.1073/pnas.1412759111 PMID:25313081

25. Walker RF, Liu JS, Peters BA, Ritz BR, Wu T, Ophoff RA, Horvath S. Epigenetic age analysis of children who seem to evade aging. Aging (Albany NY). 2015; 7:334–39. https://doi.org/10.18632/aging.100744 PMID:25991677

26. Ahrens M, Ammerpohl O, von Schönfels W, Kolarova J, Bens S, Itzel T, Teufel A, Herrmann A, Brosch M, Hinrichsen H, Erhart W, Egberts J, Sipos B, et al. DNA methylation analysis in nonalcoholic fatty liver disease suggests distinct disease-specific and remodeling signatures after bariatric surgery. Cell Metab. 2013; 18:296–302. https://doi.org/10.1016/j.cmet.2013.07.004 PMID:23931760

27. Reynolds LM, Taylor JR, Ding J, Lohman K, Johnson C, Siscovick D, Burke G, Post W, Shea S, Jacobs DR Jr, Stunnenberg H, Kritchevsky SB, Hoeschele I, et al. Age-related variations in the methylome associated with gene expression in human monocytes and T cells. Nat Commun. 2014; 5:5366. https://doi.org/10.1038/ncomms6366 PMID:25404168

28. Houseman EA, Accomando WP, Koestler DC, Christensen BC, Marsit CJ, Nelson HH, Wiencke JK, Kelsey KT. DNA methylation arrays as surrogate measures of cell mixture distribution. BMC Bioinformatics. 2012; 13:86. https://doi.org/10.1186/1471-2105-13-86 PMID:22568884

29. Horvath S. DNA methylation age of human tissues and cell types. Genome Biol. 2013; 14:R115. https://doi.org/10.1186/gb-2013-14-10-r115 PMID:24138928

30. Horvath S, Levine AJ. HIV-1 Infection Accelerates Age According to the Epigenetic Clock. J Infect Dis. 2015; 212:1563–73. https://doi.org/10.1093/infdis/jiv277 PMID:25969563

31. Horvath S, Gurven M, Levine ME, Trumble BC, Kaplan H, Allayee H, Ritz BR, Chen B, Lu AT, Rickabaugh TM, Jamieson BD, Sun D, Li S, et al. An epigenetic clock analysis of race/ethnicity, sex, and coronary heart disease. Genome Biol. 2016; 17:171. https://doi.org/10.1186/s13059-016-1030-0 PMID:27511193

32. Horvath S, Oshima J, Martin GM, Lu AT, Quach A, Cohen H, Felton S, Matsuyama M, Lowe D, Kabacik S, Wilson JG, Reiner AP, Maierhofer A, et al. Epigenetic clock for skin and blood cells applied to Hutchinson Gilford Progeria Syndrome and *ex vivo* studies. Aging (Albany NY). 2018; 10:1758–75. https://doi.org/10.18632/aging.101508 PMID:30048243

33. Ahrens M, Ammerpohl O, von Schönfels W, Kolarova J, Bens S, Itzel T, Teufel A, Herrmann A, Brosch M, Hinrichsen H, Erhart W, Egberts J, Sipos B, et al. DNA methylation analysis in nonalcoholic fatty liver disease suggests distinct disease-specific and remodeling signatures after bariatric surgery. Cell Metab. 2013; 18:296–302. https://doi.org/10.1016/j.cmet.2013.07.004 PMID:23931760

34. Reynolds LM, Taylor JR, Ding J, Lohman K, Johnson C, Siscovick D, Burke G, Post W, Shea S, Jacobs DR Jr, Stunnenberg H, Kritchevsky SB, Hoeschele I, et al. Age-related variations in the methylome associated with gene expression in human monocytes and T cells. Nat Commun. 2014; 5:5366. https://doi.org/10.1038/ncomms6366 PMID:25404168

35. Langfelder P, Horvath S. WGCNA: an R package for weighted correlation network analysis. BMC Bioinformatics. 2008; 9:559. https://doi.org/10.1186/1471-2105-9-559 PMID:19114008

36. Horvath S, Levine AJ. HIV-1 infection accelerates age according to the epigenetic clock. J Infect Dis. 2015; 212:1563–73. https://doi.org/10.1093/infdis/jiv277 PMID:25969563

37. Lu AT, Quach A, Wilson JG, Reiner AP, Aviv A, Raj K, Hou L, Baccarelli AA, Li Y, Stewart JD, Whitsel EA, Assimes TL, Ferrucci L, Horvath S. DNA methylation GrimAge strongly predicts lifespan and healthspan. Aging (Albany NY). 2019; 11:303–27. https://doi.org/10.18632/aging.101684 PMID:30669119

38. Levine ME, Lu AT, Quach A, Chen BH, Assimes TL, Bandinelli S, Hou L, Baccarelli AA, Stewart JD, Li Y, Whitsel EA, Wilson JG, Reiner AP, et al. An epigenetic biomarker of aging for lifespan and healthspan. Aging (Albany NY). 2018; 10:573–91. https://doi.org/10.18632/aging.101414 PMID:29676998

39. Hannum G, Guinney J, Zhao L, Zhang L, Hughes G, Sadda S, Klotzle B, Bibikova M, Fan JB, Gao Y, Deconde R, Chen M, Rajapakse I, et al. Genome-wide methylation profiles reveal quantitative views of human aging rates. Mol Cell. 2013; 49:359–67. https://doi.org/10.1016/j.molcel.2012.10.016 PMID:23177740

40. Bojesen SE, Pooley KA, Johnatty SE, Beesley J, Michailidou K, Tyrer JP, Edwards SL, Pickett HA, Shen HC, Smart CE, Hillman KM, Mai PL, Lawrenson K, et al, and Australian Cancer Study, and Australian Ovarian Cancer Study, and Kathleen Cuningham Foundation Consortium for Research into Familial Breast Cancer (kConFab), and Gene Environment Interaction and Breast Cancer (GENICA), and Swedish Breast Cancer Study (SWE-BRCA), and Hereditary Breast and Ovarian Cancer Research Group Netherlands (HEBON), and Epidemiological study of BRCA1 & BRCA2 Mutation Carriers (EMBRACE), and Genetic Modifiers of Cancer Risk in BRCA1/2 Mutation Carriers (GEMO). Multiple independent variants at the TERT locus are associated with telomere length and risks of breast and ovarian cancer. Nat Genet. 2013; 45:371–84, e1–2. https://doi.org/10.1038/ng.2566 PMID:23535731

41. Codd V, Nelson CP, Albrecht E, Mangino M, Deelen J, Buxton JL, Hottenga JJ, Fischer K, Esko T, Surakka I, Broer L, Nyholt DR, Mateo Leach I, et al, and CARDIoGRAM consortium. Identification of seven loci affecting mean telomere length and their association with disease. Nat Genet. 2013; 45:422–27, e1–2. https://doi.org/10.1038/ng.2528 PMID:23535734

42. Pooley KA, Bojesen SE, Weischer M, Nielsen SF, Thompson D, Amin Al Olama A, Michailidou K, Tyrer JP, Benlloch S, Brown J, Audley T, Luben R, Khaw KT, et al. A genome-wide association scan (GWAS) for mean telomere length within the COGS project: identified loci show little association with hormone-related cancer risk. Hum Mol Genet. 2013; 22:5056–64. https://doi.org/10.1093/hmg/ddt355 PMID:23900074
